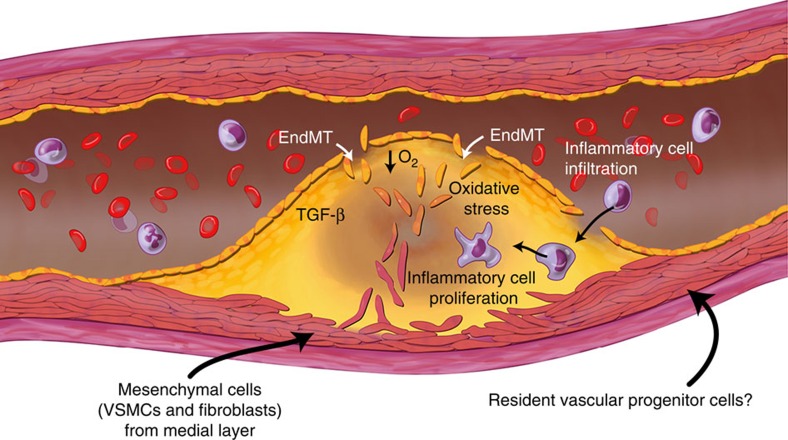# Endothelial to mesenchymal transition is common in atherosclerotic lesions and is associated with plaque instability

**DOI:** 10.1038/ncomms11853

**Published:** 2016-06-24

**Authors:** Solene M. Evrard, Laura Lecce, Katherine C. Michelis, Aya Nomura-Kitabayashi, Gaurav Pandey, K-Raman Purushothaman, Valentina d'Escamard, Jennifer R. Li, Lahouaria Hadri, Kenji Fujitani, Pedro R. Moreno, Ludovic Benard, Pauline Rimmele, Ariella Cohain, Brigham Mecham, Gwendalyn J. Randolph, Elizabeth G. Nabel, Roger Hajjar, Valentin Fuster, Manfred Boehm, Jason C. Kovacic

**Affiliations:** 1 The Zena and Michael A. Wiener Cardiovascular Institute, Icahn School of Medicine at Mount Sinai, New York, New York 10029, USA; 2 Department of Genetics and Genomic Sciences and Icahn Institute for Genomics and Multiscale Biology, Icahn School of Medicine at Mount Sinai, New York, New York 10029, USA; 3 Trialomics LLC, Seattle, Washington 98115, USA; 4 Department of Pathology and Immunology, Washington University, St Louis, Missouri 63110, USA; 5 Brigham and Women's Health Care, Boston, Massachusetts 02115, USA; 6 Marie-Josée and Henry R. Kravis Cardiovascular Health Center, Icahn School of Medicine at Mount Sinai, New York, New York 10029, USA; 7 Centro Nacional de Investigaciones Cardiovasculares (CNIC), 28029 Madrid, Spain; 8 Center for Molecular Medicine, National Heart, Lung, and Blood Institute, National Institutes of Health, Bethesda, Maryland, 20892, USA

## Abstract

Endothelial to mesenchymal transition (EndMT) plays a major role during development, and also contributes to several adult cardiovascular diseases. Importantly, mesenchymal cells including fibroblasts are prominent in atherosclerosis, with key functions including regulation of: inflammation, matrix and collagen production, and plaque structural integrity. However, little is known about the origins of atherosclerosis-associated fibroblasts. Here we show using endothelial-specific lineage-tracking that EndMT-derived fibroblast-like cells are common in atherosclerotic lesions, with EndMT-derived cells expressing a range of fibroblast-specific markers. *In vitro* modelling confirms that EndMT is driven by TGF-β signalling, oxidative stress and hypoxia; all hallmarks of atherosclerosis. ‘Transitioning' cells are readily detected in human plaques co-expressing endothelial and fibroblast/mesenchymal proteins, indicative of EndMT. The extent of EndMT correlates with an unstable plaque phenotype, which appears driven by altered collagen-MMP production in EndMT-derived cells. We conclude that EndMT contributes to atherosclerotic patho-biology and is associated with complex plaques that may be related to clinical events.

## Abstract (web summary)

Endothelial to mesenchymal transition (EndMT) is a crucial developmental process that also plays a role in the pathogenesis of some diseases. Here the authors show that EndMT contributes to the development of atherosclerosis in mice and humans, and is associated with complex human plaques that may be prone to rupture.

## Graphical abstract

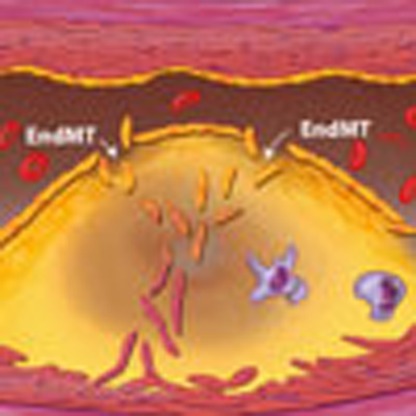

Epithelial to mesenchymal transition (EMT) and endothelial to mesenchymal transition (EndMT) are fundamental processes during development, including the cardiovascular system. Notably, only days after conception typically at the blastocyst stage, the trophoblast sends forward columns of epithelial cells that penetrate the maternal uterine wall, undergo EMT, and establish an early placental blood supply by invading the maternal decidual interstitium and vessels^1^. Thus, interestingly, there is a very early precedent for the involvement of EMT in vascular biology. Soon thereafter, EMT plays a pivotal role in germ layer specification, when cells from the primitive epiblast layer undergo EMT to give rise to mesoderm and endoderm. This central role for EMT continues throughout development, and genetic knockout mice harbouring mutations involving transforming growth factor-β (*Tgf-β*) and other central pathways governing EMT are often embryonic lethal (see reviews^2^,^3^,^4^). With specific respect to cardiac development, endocardial cells in the region of the forming atrioventricular canal undergo EndMT (a type of EMT involving endothelial cells) to give rise to mesenchymal cells that form the endocardial cushion tissue (precursor of the semilunar valves)^5^,^6^, while epicardial cells also undergo EMT to give rise to epicardium-derived cells that form smooth muscle cells, interstitial cardiac stromal cells and potentially a sub-population of endothelial cells (see reviews^2^,^4^).

In the adult, it has been increasingly appreciated that EMT and EndMT play a major role in chronic fibrosing-type injuries and diseases (see reviews^2^,^3^). In the cardiovascular system, this includes contributions to the pathologies of pulmonary hypertension^7^,^8^, transplant arteriopathy^9^,^10^, vascular malformations^11^, myocardial infarction^12^, vascular calcification,^13^ fibrodysplasia ossificans progressiva^14^ and a somewhat more controversial role^4^ in cardiac fibrosis^15^,^16^. In addition, we have shown that EndMT makes a major contribution to the vascular remodelling and neointimal formation that arises following vein graft transplantation into the arterial circulation^17^. This process is dependent on early activation of the Smad2/3-Snai2 signalling pathway, with antagonism of Tgf-β signalling resulting in decreased EndMT and reduced neointimal formation^17^. Recently, Chen *et al*.^18^ extended our understanding of EndMT and demonstrated that it promotes atherosclerosis progression, however, the principal mesenchymal cell type that is derived from EndMT in atherosclerosis was not defined by their study, nor was the extent of the contribution of EndMT-derived mesenchymal cells addressed.

Importantly, mesenchymal cells including fibroblasts are prominent in atherosclerosis, with fibroblast-specific proteins being highly expressed in advanced atherosclerotic lesions^19^,^20^. Key functions for fibroblasts in atherosclerosis include regulation of: inflammation^19^,^21^, extra-cellular matrix and collagen production^19^, and plaque structural integrity^19^,^22^. However, unlike other cell types in atherosclerotic plaques, essentially nothing is known about the origins of atherosclerosis-associated fibroblasts. Therefore, based on the fact that atherosclerosis is an archetypal example of a TGF-β-mediated fibrosing vascular disease of adults^23^,^24^, we elected to investigate whether EndMT contributes to atherosclerotic plaque formation. We identified that EndMT-derived fibroblast-like cells are common in intimal atherosclerotic plaques. These findings were corroborated using both *Cre-lox* endothelial lineage tracking in mice, and in human plaques by detecting cells co-expressing endothelial and fibroblast/mesenchymal proteins, indicative of EndMT. The number of transitioning cells was associated with an unstable and ruptured human plaque phenotype, which appears mechanistically driven by altered collagen-matrix metalloproteinase (MMP) production in EndMT-derived fibroblast-like cells. We conclude that EndMT contributes to atherosclerotic patho-biology and is associated with complex plaques that may be prone to rupture and cause clinical events.

## Results

### Endothelial lineage-tracking system in atherosclerotic mice

For endothelial lineage-tracking in the setting of EndMT, prior studies have typically used constitutively active systems, such as *Tie1Cre* or *Tie2Cre* mice^14^,^15^. While these models have robust *Cre* recombination in endothelial-derived cells, they suffer from the limitation that a majority of circulating leukocytes exhibit *Cre* recombination. In preliminary experiments we confirmed that >50% of circulating leukocytes in *Tie2Cre;R26RstopYfp* mice express Yfp (Supplementary Fig. 1a). Because atherosclerotic plaques involve a rich contribution from monocytes and other leukocytes, constitutively active endothelial lineage-tracking systems were inappropriate for studying EndMT in atherosclerosis.

**Figure 1:**
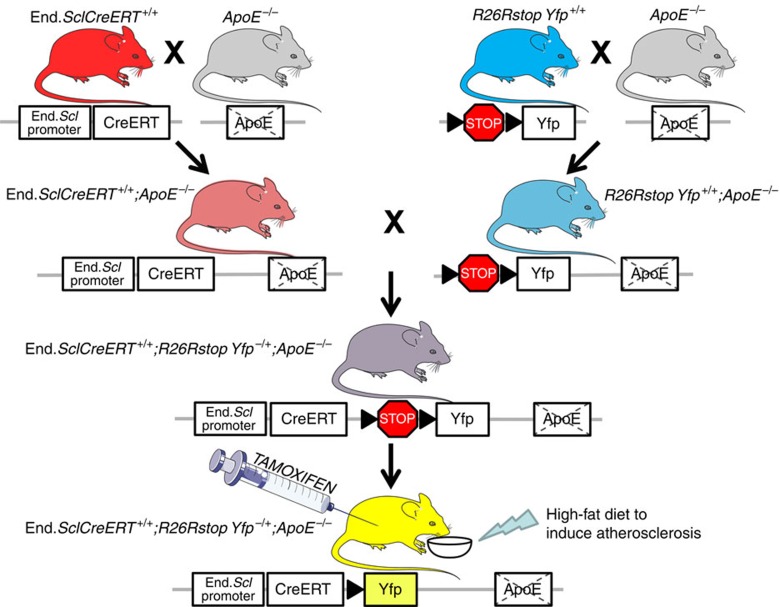
Breeding and generation of end.*SclCreER*^T^;*R26RstopYfp*;*ApoE*^*−/−*^ mice. This figure was created using Servier medical art.

We therefore created a tamoxifen-inducible endothelial lineage-tracking system; the end.*SclCreER*^T^;*R26RstopYfp*;*ApoE*^*−/−*^ mouse line (Fig. 1). The end.*SclCreER*^T^ line has been extensively validated and used to study EndMT^12^,^16^,^17^,^25^. In atherosclerosis-prone end.*SclCreER*^T^;*R26RstopYfp*;*ApoE*^*−/−*^ mice, endothelial-specific *Cre* expression is induced by tamoxifen administration to irrevocably activate the yellow fluorescence protein (*Yfp*) gene, resulting in continuous Yfp expression regardless of any change in cell phenotype^26^,^27^. As tamoxifen was administered beginning at 4 weeks of age (after the embryonic ‘hemogenic endothelial' period), *Cre* recombination in bone marrow cells and circulating leukocytes was avoided. To enhance plaque development, mice received a high-fat diet (HFD) from 6 weeks of age. Unlike constitutively active *Tie2Cre;R26RstopYfp* mice, by fluorescence-activated cell sorting (FACS) analysis of tamoxifen-induced end.*SclCreER*^T^;*R26RstopYfp* and end.*SclCreER*^T^;*R26RstopYfp*;*ApoE*^*−/−*^ mice we were unable to detect circulating CD45^+^Yfp^+^ cells (Supplementary Fig. 1b,c).

We first confirmed the sensitivity and specificity of this model for endothelial lineage-derived cell tracking. Immunofluorescence staining of Yfp and CD31 in the aortas of tamoxifen-induced end.*SclCreER*^T^;*R26RstopYfp*;*ApoE*^*−/−*^ mice after 8, 18 or 30 weeks of HFD revealed the expected pattern of Yfp expression by CD31^+^ endothelial cells in the presence of atherosclerotic lesions (Supplementary Fig. 2a–d). Consistent with prior EndMT studies using end.*SclCreER*^T^;*R26RstopYfp* mice^17^, FACS revealed that a mean of 30.7±12.5% of CD31^+^ endothelial cells in end.*SclCreER*^T^;*R26RstopYfp*;*ApoE*^*−/−*^ mice co-expressed Yfp after 8 weeks of HFD (*n*=5 mice) (Supplementary Fig. 2e). This FACS-derived data was used for all subsequent determinations of the proportion of cells undergoing EndMT. We confirmed these FACS data by immunofluorescence staining and blinded cell counting, with 53.2±12.2% of CD31^+^ endothelial cells in end.*SclCreER*^T^;*R26RstopYfp*;*ApoE*^*−/−*^ mice co-expressing Yfp after 8 weeks of HFD (*n*=5 mice). Consistent with the finding that CD45^+^ cells do not express Yfp in our model (Supplementary Fig. 1c), by immunofluorescence staining of aortic sections we were unable to detect any Yfp^+^ cells that co-expressed CD45 or CD68 (Supplementary Fig. 2f,g).

### EndMT gives rise to fibroblast-like cells in atherosclerosis

As a fundamental EndMT mediator, we sought to confirm Tgf-β expression in atherosclerotic plaques from end.*SclCreER*^T^;*R26RstopYfp*;*ApoE*^*−/−*^ mice. Consistent with its known role in atherosclerosis^23^,^24^, Tgf-β was identified within the intima and in developed plaques at all time-points and was expressed by both CD68^+^ macrophages and α-smooth muscle actin (αSma)^+^ cells (Supplementary Fig. 3a,b).

**Figure 2:**
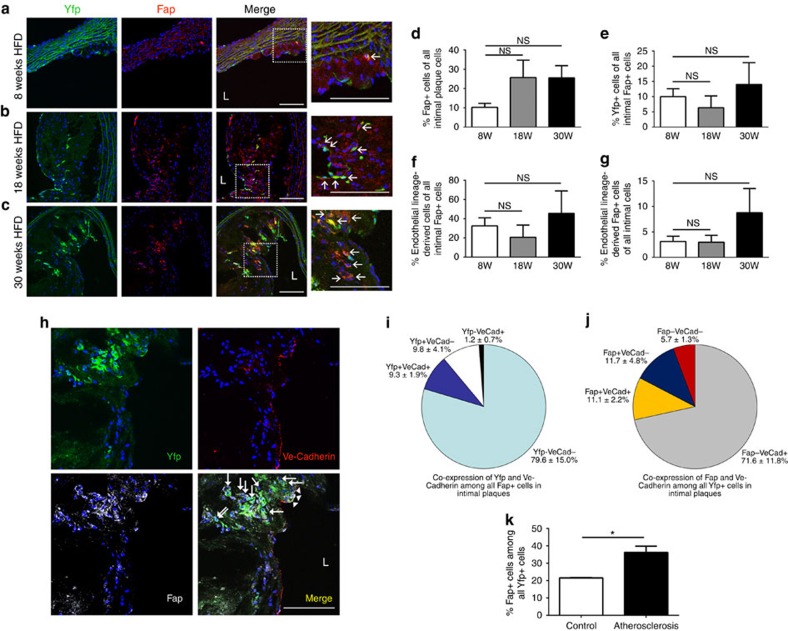
EndMT gives rise to fibroblast-like cells in intimal plaques. (**a–c**) Immunofluorescence confocal microscopy of thoracic aortic sections from tamoxifen-induced end.*SclCreER*^T^;*R26RstopYfp*;*ApoE*^*−/−*^ mice after (**a**) 8, (**b**) 18, (**c**) 30 weeks of HFD revealed Yfp^+^ cells co-expressing fibroblast-specific marker Fap within intimal plaques. L=lumen; scale bars, 100 μm. Insets at higher magnification as indicated. Arrows indicate co-positive cells. (**d**) Cell quantitation in intimal plaques revealed that after 8, 18 and 30 weeks of HFD, 10.2±2.1%, 25.7±9.0% and 25.6±6.3% (respectively) of cells expressed Fap, indicative of a fibroblast phenotype. (**e**) Quantitation (not accounting for the proportion of endothelial cells expressing Yfp) identified that at the same time-points, 10.0±2.6%, 6.4±3.9% and 14.0±7.2% (respectively) of intimal Fap^+^ cells co-expressed Yfp. (**f**) After accounting for the efficiency of our endothelial lineage tracking system (% of endothelial cells expressing Yfp), at the same time-points, we determined that 32.5±8.5%, 20.7±12.6% and 45.5±23.3% of intimal Fap^+^ cells were endothelial lineage-derived. (**g**) Again taking into account the efficiency of our endothelial lineage tracking system, at the same time-points, respectively, we determined that 3.1±1.0%, 3.0±1.4% and 8.8±4.7% of all intimal cells were endothelial lineage-derived Fap^+^ cells. For Fig. 1e–h staining was performed with at least four images evaluated from each of at least three spatially separated thoracic aortic sections per mouse (*n*=5 mice for 8 and 18 weeks; *n*=4 mice for 30 weeks). Data were averaged per animal then used for statistical analyses (one-way ANOVA). NS, not significant. (**h**) Four colour immunofluorescence confocal microscopy of thoracic aortic sections from tamoxifen-induced end.*SclCreER*^T^;*R26RstopYfp*;*ApoE*^*−/−*^ mice after 18 weeks HFD demonstrating Yfp^+^Fap^+^Ve-Cadherin^+^ (arrow heads) and Yfp^+^Fap^+^Ve-Cadherin^−^ cells (arrows). L=lumen; scale bars, 100 μm. Quantitation of Yfp^+^, Fap^+^ and Ve-Cadherin^+^ (VeCad) cells in intimal plaques as shown in **h** was performed (*n*=5 mice) to assess (**i**) the relative co-expression of Yfp and Ve-Cadherin among all Fap^+^ cells (**j**) the relative co-expression of Fap and Ve-Cadherin among all Yfp^+^ cells. (**k**) Fap expression was assessed among Yfp^+^ cells by FACS revealing that in control non-atherosclerotic end.*SclCreER*^T^;*R26RstopYfp* mice receiving chow diet, 21.5±0.2% of Yfp^+^ cells expressed Fap, while in tamoxifen-induced end.*SclCreER*^T^;*R26RstopYfp*;*ApoE*^*−/−*^ mice after 18 weeks of HFD, 36.2±3.6% of Yfp^+^ cells expressed Fap. All mice studied in panel **k** were of the same overall age (24 weeks). *P<0.05, *n*=3 per group.

To investigate whether EndMT may arise during atherosclerosis, thoracic aortic plaques from tamoxifen-induced end.*SclCreER*^T^;*R26RstopYfp*;*ApoE*^*−/−*^ mice were evaluated for cellular co-expression of Yfp and fibroblast marker proteins. We initially evaluated co-expression of the fibroblast-specific marker fibroblast activation protein (Fap), which is expressed by inflammatory- and cancer-associated stromal cells and fibroblasts^28^, and which is also expressed in atherosclerotic plaques^19^. Importantly, recent studies of genetically marked Fap^+^ cells confirmed a lack of Fap expression by endothelial cells^29^. Immunofluorescence confocal microscopy of aortic plaques revealed a significant number of endothelial lineage-derived Yfp^+^ cells co-expressing Fap (Fig. 2a–c), indicative of EndMT. Image reconstruction and z-stack analysis resolved Fap^+^Yfp^+^ co-positive cells at the single cell level, eliminating experimental artefact due to cellular overlay or superimposition (Supplementary Movie 1). We found that Fap^+^ fibroblasts constituted ∼25% of all intimal plaque cells in mature plaques after 18 or 30 weeks of HFD (Fig. 2d). After counting crude numbers of Fap^+^Yfp^+^ cells (Fig. 2e) and then correction for efficiency of Yfp expression among CD31^+^ cells in our murine model, we established that 32.5±8.5% of intimal plaque Fap^+^ cells were endothelial-derived after 8 weeks of HFD, while 45.5±23.3% of intimal Fap^+^ cells were endothelial-derived in mice with advanced atherosclerotic lesions after 30 weeks HFD (Fig. 2f). Overall, endothelial-derived Fap^+^ cells represented 3–9% of all intimal plaque cells (Fig. 2g). Differences between these groups in Fig. 2d–g were not significant, indicating a relatively constant proportional contribution of EndMT-derived cells to the intimal Fap^+^ fibroblast population. Four colour immunofluorescence confocal microscopy showed that particularly in the deeper aspect of intimal plaques (adjacent to the plaque ‘core'), Yfp^+^Fap^+^ endothelial lineage-derived cells could be identified that did not express the endothelial protein Ve-Cadherin, suggestive of endothelial transition towards a more mature fibroblast-like phenotype (Fig. 2h). Quantitation demonstrated that among the entire intimal Fap^+^ fibroblast population, 9.8±4.1% of cells were Yfp^+^ endothelial-derived cells that had lost Ve-Cadherin protein expression, while 9.3±1.9% were Yfp^+^Ve-Cadherin^+^ double positive cells, the latter likely indicative of cells transitioning from an endothelial to fibroblast-like phenotype (Fig. 2i). Conversely, among the entire Yfp^+^ endothelial-derived intimal cell population, 11.7±4.8% of cells were Fap^+^ but had lost Ve-Cadherin expression, while 11.1±2.2% were Fap^+^Ve-Cadherin^+^ double positive cells (Fig. 2j).

As fibroblasts were very rarely seen in the media (Fig. 2; Supplementary Fig. 4), we next investigated if Yfp^+^Fap^+^ endothelial lineage-derived fibroblast-like cells also exist in the adventitia during atherosclerosis (Supplementary Fig. 4a–c). In the adventitia, Fap^+^ fibroblasts constituted ∼30–35% of cells at all time-points (Supplementary Fig. 4d). After counting crude numbers of Yfp^+^Fap^+^ cells (Supplementary Fig. 4e), correction for efficiency of Yfp expression revealed that 7–16% of Fap^+^ adventitial fibroblast-like cells were derived from endothelial lineage cells (Supplementary Fig. 4f), with endothelial lineage-derived Fap^+^ cells representing 2–5% of all adventitial cells (Supplementary Fig. 4g).

We further confirmed the contribution of endothelial lineage-derived cells to Fap^+^ fibroblasts by performing FACS for Fap expression among Yfp^+^ cells. Interestingly we found that in 24 week old non-atherosclerotic end.*SclCreER*^T^;*R26RstopYfp* mice receiving chow diet, 21.5±0.2% of Yfp^+^ cells expressed Fap, while in age-matched tamoxifen-induced end.*SclCreER*^T^;*R26RstopYfp*;*ApoE*^*−/−*^ mice that received 18 weeks of HFD, 36.2±3.6% of Yfp^+^ cells expressed Fap (Fig. 2k). In distinction from our results presented so far (Fig. 2a–g; Supplementary Fig. 4) where we evaluated the proportion of Fap^+^ cells that were endothelial-derived, these data reflect the proportion of all endothelial and endothelial-derived cells that are potentially undergoing or have undergone EndMT and are expressing Fap.

These data suggested that a significant number of intimal and adventitial fibroblast-like cells in atherosclerotic lesions are derived via EndMT. These findings were confirmed using another marker, fibroblast-specific protein-1 (Fsp-1 or S100a4). While recent data has emerged suggesting Fsp-1 is not entirely specific for fibroblasts^30^, by immunofluorescence confocal microscopy, compared with Fap staining, we identified a similar number of Fsp-1^+^ cells that co-expressed Yfp (Supplementary Fig. 5; Supplementary Movie 2), supporting the occurrence of EndMT in atherosclerosis. As a third marker of fibroblasts and mesenchymal cells, immunofluorescence confocal microscopy performed using an anti-Vimentin antibody again confirmed a significant number of endothelial lineage-derived Yfp^+^ cells co-expressing this additional marker in atherosclerotic plaques (Supplementary Fig. 6).

We next sought to ascertain if endothelial lineage-derived cells might also give rise to myofibroblasts or vascular smooth muscle cells (VSMCs). While detectable, Yfp^+^ cells that also expressed the proteins αSma, Sm22α or smooth muscle myosin heavy chain (Smmhc) were less commonly observed than fibroblasts, with the proportional contribution of endothelial lineage-derived cells to αSma^+^ and Sm22α^+^ populations being ∼10-fold lower than to fibroblast-like cells (Supplementary Fig. 7).

### Oxidative stress promotes EndMT

**Figure 3:**
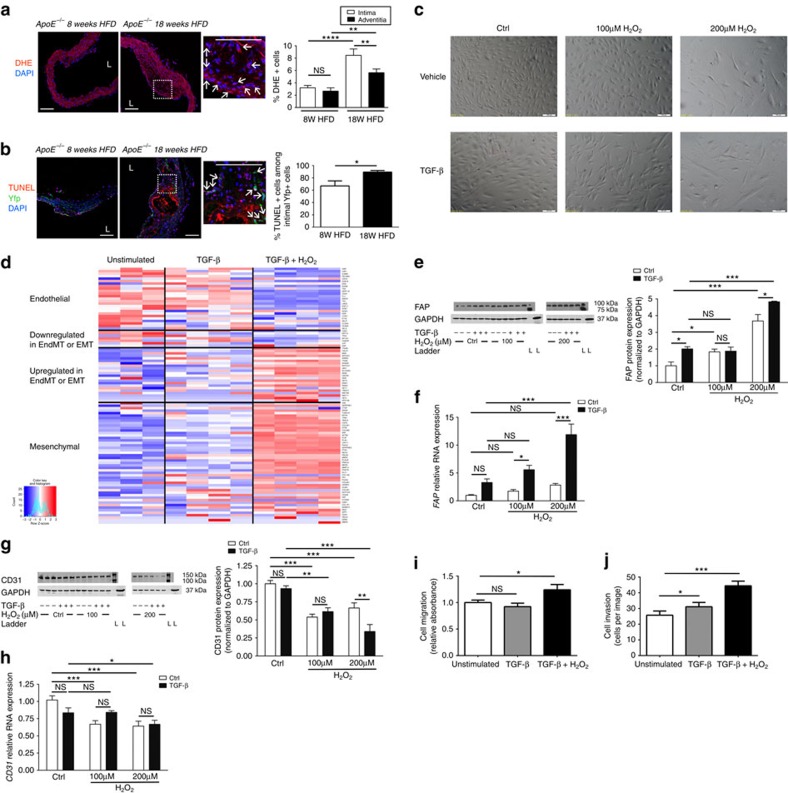
Oxidative stress promotes EndMT and has additive effects to TGF-β. (**a**) Representative dihydroethidium (DHE) microscopy images of thoracic aortic sections from tamoxifen-induced end.*SclCreER*^T^;*R26RstopYfp*;*ApoE*^*−/−*^ mice after 8 versus 18 weeks HFD demonstrating *in situ* superoxide production. Analysis by one-way ANOVA; overall *P*<0.0001. (**b**) TUNEL assay on atherosclerotic lesions from tamoxifen-induced end.*SclCreER*^T^;*R26RstopYfp*;*ApoE*^*−/−*^ mice after 8 versus 18 weeks HFD demonstrating apoptotic Yfp^+^ cells. For **a** and **b**: L=lumen; scale bars, 100 μm. Insets at higher magnification as indicated. Arrows indicate co-positive cells. Staining performed on at least three spatially separated thoracic aortic sections per mouse (*n*=5 mice per group). Control experiments from non-atherosclerotic mice shown in Supplementary Fig. 8. (**c**) Phase contrast microscopy of HUVECs grown in basal media (ctrl) or following exposure to TGF-β and/or H_2_O_2_ for 5 days. Scale bars, 100 μm. (**d**) Heatmap showing expression of endothelial genes (top), non-endothelial genes also downregulated in EndMT/EMT, genes known to be upregulated in EndMT/EMT and mesenchymal genes (bottom) for HUVECs exposed to control conditions (basal media—unstimulated), TGF-β or TGF-β plus 200 μM H_2_O_2_. Because there are no gene sets for ‘EndMT' or ‘fibroblast' in databases like GO, KEGG, Reactome or MSigDB, this gene list was compiled by extensive literature search (Supplementary Table 2). (**e**) FAP protein expression by western blot with quantification in HUVECs exposed to control (ctrl) conditions (unstimulated) or TGF-β, with and without H_2_O_2_ at 100 or 200 μM. FAP ladder (L) represents 100 kDa (upper) and 75 kDa (lower intense band). FAP expected molecular weight/size is 90 kDa. GAPDH ladder represents 37 kDa. GAPDH expected molecular weight/size is 37 kDa. (**f**) *FAP* RNA levels assessed by qRT-PCR under identical conditions. (**g**) CD31 protein by western blot with quantification. CD31 ladder (L) represents 150 kDa (upper) and 100 kDa (lower intense band). CD31 expected molecular weight/size is 130 kDa. GAPDH ladder represents 37 kDa. GAPDH expected molecular weight/size is 37 kDa. (**h**) *CD31* RNA levels assessed by qRT-PCR is presented for HUVECs under identical conditions. Data in **e**–**h** analysed by two-way ANOVA with complete results in Supplementary Table 3. (**i**) Cell migration for unstimulated HUVECs versus after treatment with TGF-β only or TGF-β+200 μM H_2_O_2_ for 5 days. Analysis by one-way ANOVA; overall *P*=0.015. (**j**) Cell invasion for unstimulated HUVECs versus after treatment with TGF-β only or TGF-β+200 μM H_2_O_2_ for 5 days. Analysis by one-way ANOVA; overall *P*<0.0001. *n*=6–9 for qRT-PCR and 3–6 for western blots. Migration and invasion determined from three independent experiments with each experiment having *n*=3. NS, not significant, *P<0.05, ^**^P<0.01, ^***^P<0.001, ^****^P<0.0001.

We next investigated the molecular mechanisms underlying endothelial lineage specification of fibroblast-like cells during EndMT. While its role in EndMT is essentially unknown, prior studies have documented that oxidative stress is an important factor during the related process of EMT^31^,^32^, and we speculated that this may be of relevance during EndMT in atherosclerotic plaques. We first identified that intimal plaques from end.*SclCreER*^T^;*R26RstopYfp*;*ApoE*^*−/−*^ mice exhibit marked oxidative stress, which was significantly greater in more advanced lesions (Fig. 3a). While oxidative stress was also observed in the adventitia, in vessels from mice with advanced atherosclerosis there was significantly less oxidative stress observed in the adventitia than in intimal plaques (Fig. 3a). We also performed TUNEL staining to assess for apoptosis, and found substantial intimal Yfp^+^ cell apoptosis in early plaques, that was significantly increased in more advanced intimal plaques (Fig. 3b). Control vessels from non-atherosclerotic mice did not show appreciable oxidative stress or cell apoptosis (Supplementary Fig. 8). We proceeded to evaluate the independent and combined effects of TGF-β (a known EndMT mediator) and/or hydrogen peroxide (H_2_O_2_) (an inducer of oxidative stress) on human umbilical vein endothelial cells (HUVECs). Exposure to TGF-β or H_2_O_2_ caused a dose-responsive change in morphology, with HUVECs progressively losing their cobblestone appearance and adopting a dispersed, spindle-shaped morphology, with these changes being further enhanced when TGF-β and H_2_O_2_ were applied together (Fig. 3c). When TGF-β alone was applied, at the dose used to induce EndMT there was no change in cell count, proliferation, apoptosis or death. However, consistent with the induction of cellular stress, H_2_O_2_ caused a dose-responsive decrease in cell number, with a reduction in cell proliferation and an increase in cell apoptosis and death (Supplementary Fig. 9; Supplementary Table 1). Global transcriptome analysis identified that the application of TGF-β alone to HUVECs caused the upregulation of a total 1,363 genes/transcription factors and downregulated 1,295, while the application of TGF-β plus 200 μM H_2_O_2_ upregulated 3,366 genes/transcription factors and downregulated 2,934 (significance analysis of microarrays-based differential expression^33^,^34^; all *P*<0.05 after correction for multiple comparisons). Among these, a broad range of EndMT/EMT-related and mesenchymal genes were upregulated by TGF-β alone (Fig. 3d; Supplementary Table 2). However, the combined application of TGF-β+H_2_O_2_ produced a greater, additive induction of EndMT with concurrent downregulation of endothelial gene expression (Fig. 3d; Supplementary Table 2). These findings were verified by quantitative real-time PCR (qRT-PCR) and western blot, with H_2_O_2_ activating EndMT in HUVECs in an incremental and dose-responsive manner that was significantly more effective than TGF-β alone (Fig. 3e–h; Supplementary Tables 3 and 4; Supplementary Fig. 10a,b). Consistent with the transition to a mesenchymal phenotype, HUVECs treated with TGF-β+H_2_O_2_ demonstrated enhanced migration and invasion as compared to unstimulated control HUVECs (Fig. 3i,j).

We then further investigated expression and activation of signalling pathways regulating EndMT in the vasculature^17^. Supporting our hypothesis that oxidative stress promotes EndMT in atherosclerotic plaques, TGF-β+H_2_O_2_ caused an additive and significant increase in the RNA expression level of TGF-β signalling pathway member and EndMT mediator *SNAI2* (Supplementary Fig. 10c). On the other hand, *SNAI1* was only increased by TGF-β, while *SMAD2* levels were not affected by either TGF-β or H_2_O_2_. Expression of *SMAD3* was increased by H_2_O_2_ but unexpectedly decreased by TGF-β (Supplementary Fig. 10d–f). Western blot analysis confirmed that H_2_O_2_ had an independent effect on SMAD3 protein levels in HUVECs, while there was no change in SMAD3 protein levels in response to TGF-β. Moreover, both H_2_O_2_ and TGF-β led to an increase in SMAD3 phosphorylation (Supplementary Fig. 10g; Supplementary Table 4).

Analysis of pathways that were enriched in upregulated genes indicated that at the doses used for EndMT induction, TGF-β alone did not activate any specific pathways in HUVECs (Supplementary Fig. 11a). However, TGF-β+H_2_O_2_ caused upregulation of a number of pathways (Supplementary Fig. 11b). Consistent with our finding that oxidative stress mediates EndMT, many of the pathways upregulated by TGF-β+H_2_O_2_ were related to fibrosis (for example, hepatic fibrosis / hepatic stellate cell activation), inflammation (for example, leukocyte extravasation signalling) or oxidative stress itself (for example, NRF2-mediated oxidative stress response). Interestingly, we found that several interferon-related pathways were upregulated, while the most significantly upregulated pathway was the protein ubiquitination pathway (Supplementary Fig. 11b). While we are unaware that ubiquitination or interferon signalling have been investigated during EndMT, both have been implicated in EMT^35^,^36^,^37^.

Because EndMT in the context of atherosclerosis requires that arterial (rather than venous) endothelial cells transition to a mesenchymal state, we evaluated the ability of human coronary artery endothelial cells (HCAECs) to undergo EndMT. Mirroring our results with HUVECs, H_2_O_2_ with or without TGF-β demonstrated an incremental effect on EndMT induction in HCAECs (Supplementary Fig. 12).

### Severe hypoxia promotes EndMT

**Figure 4:**
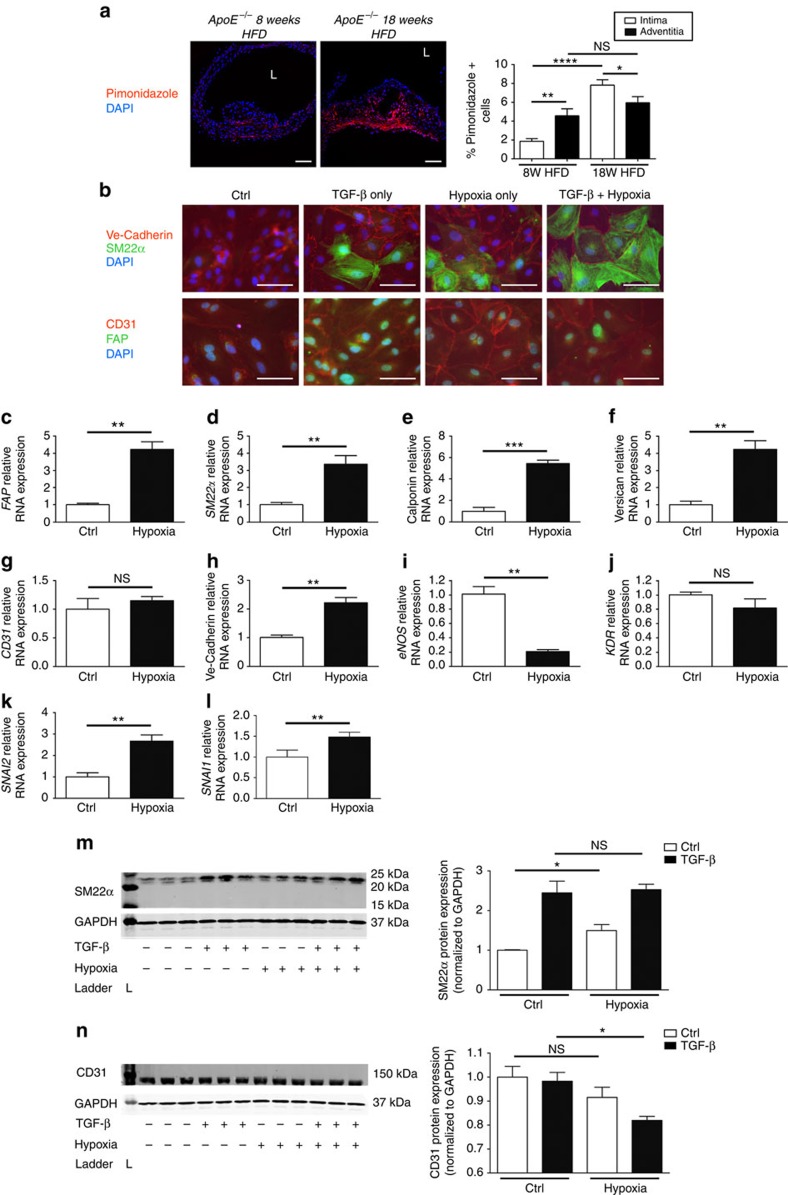
Hypoxia increases EndMT and is additive to the effect of TGF-β. (**a**) Representative pimonidazole staining demonstrating the extent of hypoxia in aortic sections from tamoxifen-induced end.*SclCreER*^T^;*R26RstopYfp*;*ApoE*^*−/−*^ mice fed 8 versus 18 weeks HFD. Staining in red is indicative of hypoxia with pO_2_ <10 mm Hg. Specific staining was performed using pimonidazole (seen in red) and DAPI (blue). L=lumen; scale bars, 100 μm. Staining was performed on at least three spatially separated thoracic aortic sections per mouse (*n*=5 mice per group). Analysis by one-way ANOVA; overall *P*<0.0001. (**b**) Immunostaining of HCAECs exposed to hypoxia and/or TGF-β for 5 days *in vitro*. Scale bars, 100 μm. (**c**–**f**) Relative RNA expression of mesenchymal genes *FAP*, *SM22α*, Calponin and Versican (respectively) in HCAECs was increased by hypoxia as assessed by qRT-PCR. (**g**–**j**) Relative RNA expression of endothelial genes *CD31*, Ve-Cadherin, *eNOS* and *KDR* assessed by qRT-PCR showed mixed effects in response to hypoxia. (**k**–**l**) Relative RNA expression of *SNAI2* and *SNAI1* in HCAECs was increased by hypoxia. (**m**) SM22α protein expression, assessed by western blot in HCAECs, was increased by hypoxia as indicated. SM22α ladder (L) represents 25 kDa (upper), 20 kDa (middle) and 15 kDa (lower). SM22α expected molecular weight/size is 23 kDa. GAPDH ladder represents 37 kDa. GAPDH expected molecular weight/size is 37 kDa. (**n**) CD31 protein expression, assessed by western blot in HCAECs. CD31 ladder (L) represents 150 kDa. CD31 expected molecular weight/size is 130 kDa. GAPDH ladder represents 37 kDa. GAPDH expected molecular weight/size is 37 kDa. As our intent was to evaluate the specific effect of hypoxia, and not interactions with TGF-β (which is provided as a reference), *t* testing was performed in this case. *n*=3 in all experiments. All experiments replicated >3 times. NS, not significant, *P<0.05, ^**^P<0.01, ^***^P<0.001, ^****^P<0.0001.

As another mediator of oxidative stress, hypoxia is a fundamental aspect of atherosclerotic biology. While it is known to promote EMT^38^, the effects of hypoxia on EndMT have not been extensively assessed. We initially verified that hypoxia was present in atherosclerotic plaques of HFD-fed end.*SclCreER*^T^;*R26RstopYfp*;*ApoE*^*−/−*^ mice, finding that with progressive atherosclerosis there is a marked increase in the percentage of hypoxic cells in intimal plaques (Fig. 4a). Furthermore we found that in mature plaques there are more hypoxic cells in intimal plaques than in the adventitia (Fig. 4a). We next proceeded to evaluate the effect of hypoxia on EndMT. Immunostaining of HCAECs exposed to hypoxia, TGF-β, or both hypoxia and TGF-β, indicated that EndMT arose under each of these conditions (Fig. 4b). Evaluation of RNA expression demonstrated that hypoxia caused a 3–6-fold increase in the expression of a range of fibroblast and mesenchymal markers (Fig. 4c–f). Interestingly, the effect of hypoxia on the expression of certain endothelial genes was borderline (Fig. 4g–j), and in a single case went in an unexpected direction (Fig. 4h), which we expect may be due to the fact that modest hypoxia is known to promote endothelial gene expression^39^. Therefore severe hypoxia, as was applied in this case and as is relevant to atherosclerosis, appears to promote mesenchymal gene expression while having a mixed effect on endothelial genes. Nevertheless, consistent with EndMT, both *SNAI2* and *SNAI1* were upregulated by hypoxia in HCAECs (Fig. 4k,l). Western blot analyses further verified these observations (Fig. 4m,n).

### EndMT occurs in human atherosclerotic plaques

**Figure 5:**
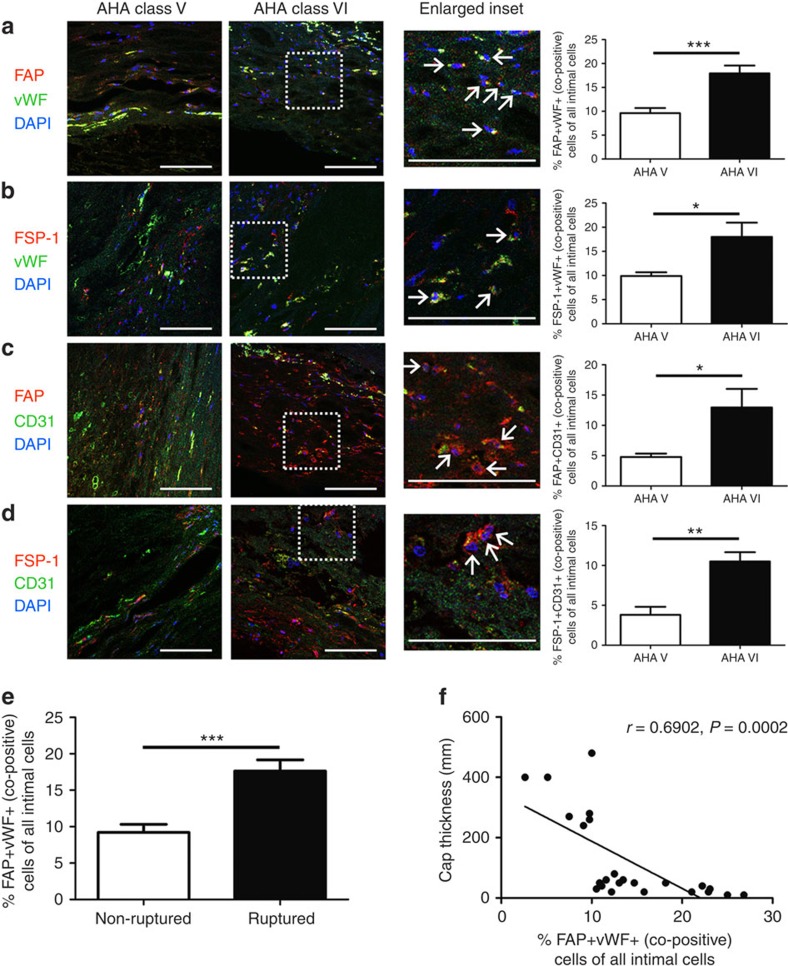
EndMT occurs in human atherosclerosis and is common in complex plaques. Plaques identified in human samples obtained at autopsy from the abdominal aorta were classified as AHA type V or VI according to standard criteria^40^. Staining was performed using various endothelial–mesenchymal marker combinations as indicated. The % of co-positive cells per × 20 field was expressed as a function of the total number of DAPI^+^ cells (per field) as follows: (**a**) FAP/vWF, (**b**) FSP-1/vWF, (**c**) FAP/CD31, (**d**) FSP-1/CD31. Scale bars, 100 μm. Insets are shown at higher magnification as indicated and arrows indicate co-positive cells. (**e**) Percentage of FAP^+^vWF^+^ co-positive cells presented according to ruptured versus non-ruptured plaque morphology. *P<0.05, ^**^P<0.01, ^***^P<0.001. (**f**) Scatter plot and regression line for aortic plaque cap thickness versus % FAP^+^vWF^+^ co-positive cells. *r*=0.6902, *P*=0.0002. For FAP/vWF, 24 randomly selected plaques from 16 patients were assessed (11 type V, 13 type VI), while for other combinations a minimum of 10 plaques from at least 6 different patients was randomly included per analysis.

*In vivo* lineage tracking of EndMT is not currently possible in humans. However, as we observed *in vitro* and in our end.*SclCreER*^T^;*R26RstopYfp*;*ApoE*^*−/−*^ model, cells undergoing EndMT can be identified at various stages of transition, including cells that simultaneously express endothelial and mesenchymal markers (Fig. 2h–j). Therefore, to determine if EndMT is operative in human atherosclerosis we evaluated and compared plaques classified as type V (fibrocalcific lesions including fibroatheroma) versus type VI (complicated lesions with vulnerable/unstable features)^40^ from human aortic post-mortem samples for cells that co-express endothelial and mesenchymal proteins. Using various combinations of markers, we identified cells that expressed both endothelial and fibroblast proteins, indicative of cells undergoing EndMT. Quantitation of cells expressing combinations of FAP/vWF, FSP-1/vWF, FAP/CD31 or FSP-1/CD31 revealed that in the order of 4–10% of intimal cells in type V lesions co-expressed endothelial and fibroblast proteins, compared with 10–18% of intimal cells in type VI lesions (Fig. 5a–d). Importantly, differences between type V and VI plaques were significant for each of the endothelial-fibroblast marker combinations tested, indicating that a greater proportion of cells are actively undergoing EndMT in complex and unstable human atherosclerotic plaques (Fig. 5a–d). We further identified that an increased proportion of FAP^+^vWF^+^ co-positive cells were present in ruptured versus non-ruptured plaques (Fig. 5e), and supporting this observation, found an inverse relationship between plaque fibrous cap thickness and % co-positive FAP^+^vWF^+^ cells within the plaque (Fig. 5f). The inverse relationship between plaque fibrous cap thickness and % co-positive FAP^+^vWF^+^ cells persisted when the analysis was limited to plaques with cap thickness of <100 μm (Supplementary Fig. 13). This observation indicates a direct relationship between the proportion of cells in human plaques undergoing EndMT and a rupture-prone plaque phenotype with thin fibrous cap^41^.

We also performed co-staining of AHA type V plaques (which are typically less inflammatory and with more VSMCs than type VI lesions)^22^ with endothelial and VSMC markers. Similar to our murine data, while ∼10-fold less common than cells co-expressing endothelial and fibroblast markers, we identified that cells co-expressing endothelial and VSMC markers constituted ∼1% of all intimal plaque cells in AHA type V human plaques (Supplementary Fig. 14).

### EndMT is associated with altered collagen-MMP production

**Figure 6:**
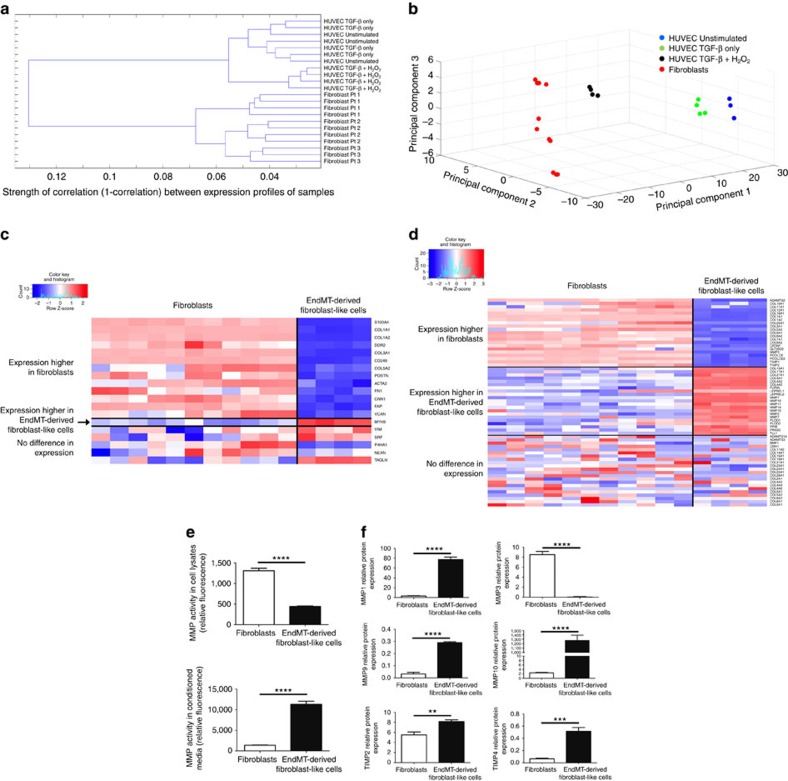
EndMT leads to a fibroblast-like phenotype with altered collagen-MMP balance. (**a**) Hierarchical cluster analysis based on global gene expression demonstrating that untreated and TGF-β-only-treated HUVECs are intermixed and clustered together, while HUVECs treated with TGF-β+H_2_O_2_ cluster separately. Human fibroblasts clustered separately again to all endothelial-derived cells (untreated, TGF-β-only- and TGF-β+H_2_O_2_-treated HUVECs). *x* axis (1-correlation) indicates strength of correlation. Each data point (leaf of dendrogram) represents a separate sample for those conditions. (**b**) Principal component analysis for HUVECs (untreated, TGF-β-only and TGF-β+H_2_O_2_ treated) and human fibroblasts based on genes presented in Fig. 3d and Supplementary Table 2. (**c**) Comparison of genes and transcription factors expressed by fibroblasts and EndMT-derived fibroblast-like cells (TGF-β+H_2_O_2_-treated HUVECs). Expression levels of 5 of 19 mesenchymal/fibroblast genes were statistically indistinguishable between these cell populations, while a single gene (*MYH9*) was more highly expressed in EndMT-derived fibroblast-like cells. (**d**) Comparison of expression of genes involved in extracellular matrix organization between human fibroblasts and EndMT-derived fibroblast-like cells demonstrating differing balance between collagen and MMP transcript production between these populations. (**e**) Global MMP protein activity assay, demonstrating 3-fold lower MMP activity in the cell lysates of EndMT-derived fibroblast-like cells but an 8.2-fold increase in MMP activity in the conditioned media from these cells, compared with human fibroblasts; ^****^P<0.0001; *n*=4 in all groups. (**f**) Conditioned media from EndMT-derived fibroblast-like cells exhibited greater protein levels of MMP1, 9 and 10 and TIMP2 and 4, compared with human fibroblast conditioned media. Conversely, fibroblast conditioned media had higher levels of MMP3. The levels of MMP2 and TIMP1 were not different between cell populations, while MMP8 and MMP13 were not present at detectable levels (not shown). ^**^P<0.01, ^***^P<0.001, ^****^P<0.0001; *n*=4 in all groups.

We sought a mechanistic explanation for our observation that an increased proportion of cells appeared to be undergoing EndMT in complex and unstable human atherosclerotic plaques and undertook to compare the molecular characteristics of EndMT-derived cells with established human fibroblast lines (Supplementary Fig. 15). For this, global transcriptome analysis was used to compare primary human fibroblasts with HUVECs, HUVECs treated with TGF-β only and HUVECs that were induced to undergo EndMT by TGF-β+H_2_O_2_ according to our *in vitro* model. Hierarchical clustering demonstrated that while unstimulated and TGF-β-only treated HUVECs cluster together and are intermixed on the resulting dendrogram, HUVECs treated with TGF-β+H_2_O_2_ cluster separately (Fig. 6a). However, human fibroblasts clustered separately again from all endothelial-derived cells (unstimulated, TGF-β-only and TGF-β+H_2_O_2_-treated HUVECs) (Fig. 6a). This suggests that cells that undergo EndMT acquire a gene expression pattern that is different from an endothelial cell or fibroblast, which we henceforth refer to as EndMT-derived fibroblast-like cells. To understand and visualize this difference in lower dimensions, we performed principal component analysis across all cell types using the same terms as Fig. 3d (genes and transcription factors associated with EndMT/EMT). Visualization of the first three principal components demonstrated that while HUVECs under unstimulated and TGF-β-treated conditions are quite similar, EndMT-derived fibroblast-like cells (TGF-β+H_2_O_2_-treated HUVECs) clustered between human fibroblasts and unstimulated HUVECs (Fig. 6b). These data indicate that the molecular gene expression pattern of EndMT-derived fibroblast-like cells differs markedly from unstimulated endothelial cells, and that it is intermediary with classical fibroblasts.

We next compared mesenchymal and fibroblast gene expression levels between human fibroblasts and EndMT-derived fibroblast-like cells (TGF-β+H_2_O_2_ treated HUVECs). Supportive of a fibroblast-like state of EndMT-derived cells, while human fibroblasts expressed higher levels of 13 of 19 mesenchymal/fibroblast genes, the expression levels of 5 of 19 mesenchymal/fibroblast genes were equivalent between these cell populations, and a single gene (*MYH9*) was more highly expressed in EndMT-derived fibroblast-like cells (Fig. 6c). To understand the mechanisms whereby EndMT-derived fibroblast-like cells are associated with a complex and unstable plaque phenotype, we compared gene ontology (GO) extracellular matrix terms between human fibroblasts and EndMT-derived fibroblast-like cells, revealing significant differences in extracellular matrix gene expression patterns between these populations (Fig. 6d). Compared with EndMT-derived fibroblast-like cells, human fibroblasts expressed higher levels of collagens (*collagen 1A1*, *1A2*, *3A1*, *5A2*, *6A1*, *6A2*, *7A1*, *8A2*, *10A1*, *11A1*, *12A1*, *16A1*, *22A1*), but lower levels of every *MMP* except *MMP3*. In contrast, we found the opposite pattern in EndMT-derived fibroblast-like cells, with significantly greater expression of numerous *MMPs* (*MMP 1*, *2*, *7*, *10*, *11*, *14*, *15*) but fewer collagens (*collagen 4A1*, *4A2*, *4A5*, *13A1*, *17A1*, *27A1*), as compared with human fibroblasts (Fig. 6d). These findings were confirmed at the protein level, with an 8.2-fold increase in MMP activity seen in conditioned media obtained from EndMT-derived fibroblast-like cells compared with human fibroblasts (Fig. 6e). Interestingly, there was 3-fold less MMP activity in the cell lysates of EndMT-derived fibroblast-like cells compared with human fibroblasts, suggesting that in addition to increased MMP gene transcription, the global increase in MMP activity seen in the conditioned media of EndMT-derived fibroblast-like cells is likely to also be due to increased MMP release (Fig. 6e). These findings were further confirmed by examining protein levels of specific MMPs, with conditioned media from EndMT-derived fibroblast-like cells showing significantly higher levels of MMPs 1, 9 and 10 as compared with human fibroblasts (Fig. 6f). Given that collagen is a hallmark of atherosclerotic plaque stability^42^,^43^, while MMPs are destabilizing^44^,^45^, this provides a molecular mechanism for our human observations, indicating that by altering the collagen-MMP balance EndMT-derived fibroblast-like cells may destabilize atherosclerotic lesions and enhance clinical disease progression towards a collagen-poor, rupture-prone plaque phenotype with thin fibrous cap.

## Discussion

In summary, we identified that EndMT-derived fibroblast-like cells make a significant contribution to atherosclerotic plaques, and that the molecular signature of these cells indicates an important role in plaque destabilization. These findings are consistent with numerous studies that have demonstrated a role for EndMT or EMT in other chronic adult fibrosing conditions^2^,^3^,^7^,^8^,^9^,^10^,^11^,^13^,^14^,^15^,^17^. While other cellular contributions to atherosclerosis have been controversial, the provisioning of cells by EndMT was anticipated^46^,^47^,^48^ but difficult to prove until the present time^18^. Recently, a single publication emerged which suggested that EndMT is modulated by FGF receptor 1 signalling, and that this process is important during atherosclerosis^18^. However, while an important contribution in its own right, that study did not address the mesenchymal phenotype of the EndMT-derived cells or quantitate the contribution of those cells to atherosclerotic lesions, nor did it address the role of oxidative stress and collagen-MMP balance.

Collectively, our *in vitro* and *in vivo* mouse data suggest that EndMT-derived cells are present in intimal plaques throughout atherosclerotic development and that this process is driven by TGF-β signalling, oxidative stress and hypoxia. While it is well known that apoptosis is abundant in atherosclerotic lesions^49^, it was notable that a substantial proportion of intimal Yfp^+^ cells in advanced plaques appeared to be undergoing apoptosis (Fig. 3b). Potentially, while increases in oxidative stress (Fig. 3a) and hypoxia (Fig. 4a) with athero-progression may promote EndMT, enhanced apoptosis may act as a compensatory mechanism to maintain a relatively constant proportion of intimal EndMT-derived cells in advanced lesions (Fig. 2).

The precise extent of EndMT varied somewhat depending on the conditions and markers studied. In mice, among all intimal cells, endothelial-derived Fap^+^ cells represented 3–9% cells (Fig. 2g), whereas endothelial-derived Fsp-1^+^ cells represented 3.0–4.5% of cells and endothelial-derived Vimentin^+^ cells represented 10–26% of cells (Supplementary Figs 5g and 6g, respectively). On the other hand, in humans we found that 4–18% of cells in intimal plaques co-expressed endothelial and fibroblast markers (Fig. 5), which was dependent on both the marker combination used and lesion classification (type V versus VI). As important limitations, Vimentin may also mark activated endothelial cells as well as fibroblasts and transitioned fibroblast-like cells^50^, while vWF may overestimate the proportion of endothelial cells^51^. Furthermore, this variability we observed almost certainly reflects the fact that there are various fibroblast sub-types (that are likely to be marked differently by our various antibodies) and that there is no single ‘universal' fibroblast marker. As a final consideration, in contrast to humans, in mice plaques typically do not rupture and the identification of vulnerable and unstable plaques is rarely performed because it is arbitrary and of uncertain relevance. Therefore, while it appears from our human data that unstable and ruptured plaques exhibit more EndMT, this must be distinguished from our murine data, where we found that plaques from mice with early versus advanced atherosclerosis (8, 18 or 30 weeks of HFD) had a similar extent of EndMT. Overall, taking these many factors into account, we estimate that the true extent of endothelial-derived fibroblast-like cells in atherosclerotic intimal lesions is in the order of 5–15%, which in humans is further influenced by the morphological nature of the plaque itself (stable versus unstable, ruptured versus non-ruptured and so on).

Our data inform our understanding of the context- and milieu-specific nature of EndMT. In our *in vitro* models, we observed a differing degree of cell transitioning and final mesenchymal phenotype depending on the specific EndMT-inducing conditions (that is, TGF-β±H_2_O_2_±hypoxia) indicating that, while different factors may trigger EndMT, the resulting mesenchymal phenotype is variable and at least partially dependent on the nature and strength of the inciting stimulus. Our *in vivo* data similarly suggest that the mesenchymal cell phenotype that arises via EndMT is milieu- and/or context-dependent, such that VSMCs rarely arise from EndMT in plaques from *ApoE*^*−/−*^ mice, but that the differing milieu of a human plaque is conducive to EndMT giving rise to both fibroblast-like cells and a smaller number of VSMCs. This is consistent with the broader cardiovascular EndMT literature, with some investigators showing that EndMT gives rise to fibroblasts^15^, while in different disease models we and others have identified that EndMT may give rise to VSMCs^9^,^17^ or even other mesenchymal cell types such as osteoprogenitor cells^13^,^14^. Furthermore, our finding that EndMT gives rise to fibroblast-like cells, but not to fully mature fibroblasts, is consistent with recent data indicating that renal epithelial cells contribute to kidney fibrosis by undergoing a partial EMT to adopt a myofibroblast-like phenotype^52^,^53^. Further research will be required to fully unravel the molecular cues that govern an EMT/EndMT event, which we speculate may ultimately reveal that cell fate is determined by both cell extrinsic factors (for example, milieu, stimulus) and cell intrinsic factors (for example, epigenetic marks, cell metabolic status). Similarly, it will be essential to explore and characterize the interaction of these molecular cues with plaque development, and conversely the impact of plaque progression and regression on the EndMT process.

Atherosclerosis is one of the most common causes of morbidity and mortality in the world^54^. Our data call for a revision of the current model of the cellular basis of this disease, which previously included only proliferating terminally differentiated local cells, infiltrating inflammatory cells and a putative but poorly defined role for stem/progenitor cells. Our study has expanded this framework and shown that phenotypic cell switching by EndMT is a major additional feature of atherosclerosis (Fig. 7), with EndMT potentially driving this disease by altering collagen-MMP balance. The fact that in humans EndMT is more common in complex lesions and ruptured plaques argues for the clinical importance of this process.

## Methods

### Mice

Endothelial cell fate tracking experiments in atherosclerosis-prone mice were performed using the tamoxifen-inducible end.*SclCreER*^T^;*R26RstopYfp*;*ApoE*^*−/−*^ murine line. With respect to the end.*SclCreER*^T^ mouse line^25^, stem cell leukemia (*Scl*) is a transcription factor expressed in endothelium and in subsets of hematopoietic cells and the brain. Two distinct elements within the *Scl* locus guide expression to endothelial cells. An *Scl* 5′ endothelial enhancer directs expression to endothelium, while a *Scl* 3′ enhancer is responsible for *Scl* expression in early hematopoietic progenitors and embryonic endothelium.^25^ In the end.*SclCreER*^T^ mouse line the creators used a 5′ endothelial enhancer to achieve endothelial-specific expression of the tamoxifen-inducible recombinase *Cre-ER*^*T*^^25^. The final triple-crossed experimental end.*SclCreER*^T^;*R26RstopYfp*;*ApoE*^*−/−*^ mouse line was created by crossing *ApoE*^*−/−*^ mice with both end.*SclCreER*^T^ and *R26RstopYfp* mice to create end.*SclCreER*^T^;*ApoE*^*−/−*^ and *R26RstopYfp*;*ApoE*^*−/−*^ mice. These double-crossed lines were then interbred to create end.*SclCreER*^T^;*R26RstopYfp*;*ApoE*^*−/−*^ mice (Fig. 1). We also created end.*SclCreER*^T^;*R26RstopYfp* mice during preliminary studies and as controls. Mice were sourced as follows: end.*SclCreER*^T^ mice^25^ were obtained from the laboratory of Dr Manfred Boehm with the permission of the Telethon Institute for Child Health Research and the creators Dr Glenn Begley and Dr Joachim Goethert; *Tie2Cre* (stock number 008863), *R26RstopYfp* (stock number 006148), *ApoE*^*−/−*^ (stock number 002052) and WT mice were obtained from the Jackson Laboratory (Bar Harbor, ME). All mouse lines were on a C57BL/6 genetic background. Induction of *Cre* in end.*SclCreER*^T^ mice was achieved by intraperitoneal injection of 1 mg tamoxifen (T5648, Sigma, St Louis, MO) dissolved in sterile peanut oil once daily for 1 week in 4-week-old mice, followed by 1 week off, then a second course of injections daily for 1 week. Control mice received peanut oil only over the same intraperitoneal injection schedule. To induce atherosclerosis, 6-week-old end.*SclCreER*^T^;*R26RstopYfp*;*ApoE*^*−/−*^ mice were commenced on a HFD (#88137; Harlan Laboratories, Indianapolis, IN). Genotyping was performed by PCR amplification using allele-specific primers (Supplementary Table 6). All procedures and animal care were approved by our institutional animal use committee and conformed to the animal care guidelines at the Icahn School of Medicine at Mount Sinai.

While because of its direct relevance to the extent of atherosclerosis we report mice in terms of the period of HFD received (8, 18 or 30 weeks of HFD), because HFD-feeding was always commenced at 6 weeks of age, these three groups of mice were always 14, 24 and 36 weeks of age, respectively. In accord with US National Institutes of Health guidelines, mice were evenly balanced between males and females for every experiment^55^.

### Mouse tissue fixation and immunohistochemistry

The thoracic aorta was harvested from mice following 8, 18 or 30 weeks of HFD (at 14, 24 or 36 weeks of age). After humane euthanasia, the thorax was entered and the heart swiftly exposed. A generous rent was created in the right atrium. The left ventricle was punctured using a 20-gauge needle, and 20 ml of 1 × PBS at 4 °C was infused at ∼2.5 ml min^−1^. Tissues were then fixed *in situ* by perfusing the heart with 20 ml of an ice-cold solution of 0.1% glutaraldehyde and 1.5% paraformaldehyde in PBS. Harvested tissues were placed in 20% sucrose overnight at 4 °C, and then embedded in Tissue Tek OCT embedding medium (#62550-01; Electron Microscopy Sciences, Hatfield, PA). Tissue blocks were kept at −80 °C and were subsequently sectioned at 10-μm thickness on a Leica cryostat (CM3050S, Leica, Allendale, NJ) and placed on glass slides^56^. To avoid inhomogeneity of the experimental substrate, only the thoracic aorta was harvested and analysed (including for FACS analysis—see below).

For immunostaining on frozen sections, thawed slides were washed in PBS and blocked using 20% donkey serum and 5% BSA in PBS with 0.1% Triton-100. Diluted primary antibodies were applied overnight at 4 °C as follows: anti-CD31 (#550274; BD Biosciences, San Jose, CA, 1:100), anti-Ve-Cadherin (sc-6458; Santa Cruz, Dallas, TX, 1:50), anti-Fap (ab53066; Abcam, Cambridge, MA, 1:100), anti-Fsp-1 (ab27957; Abcam, 1:400), anti-αSma (ab5694; Abcam, 1:200), anti-Sm22α (ab14106; Abcam, 1:200), Cy3-conjugated anti-αSma (C6198; Sigma, 1:400), anti-Smmhc (BT-562; Biomedical Technologies, Stoughton, MA, 1:200), FITC-conjugated anti-Gfp/Yfp (ab6662; Abcam, 1:150), anti-Gfp/Yfp (ab13970; Abcam, 1:500), anti-CD68 (MCA1957; Abd Serotec, Raleigh, NC, 1:200), anti-CD45 (#550539, BD Biosciences, 1:200), anti-Tgf-β (ab66043; Abcam, 1:100), anti-Vimentin (ab24525; Abcam, 1:100). After washing with PBS three times, diluted secondary antibodies (donkey Alexa Fluor 488, 546, or 594 (Invitrogen, Grand Island, NY, 1:750) or goat Alexa Fluor 488, 546, 633 (Invitrogen, 1:750)) were applied for 1 h at room temperature. Slides were mounted using DAPI-containing media (Vector Laboratories, Burlingame, CA). Control slides were routinely stained in parallel by substituting IgG, or the specific IgG isotype, from the same species for the primary antibody at the same final concentration. Slides were imaged using a confocal microscope (SP5 DM; Leica, or, LSM 510 Meta; Zeiss, Jena, Germany). Arterial elastic laminae in the medial aortic layer are visible in green and occasionally in red due to auto-fluorescence. For cell counting, images were independently coded and then analysed by an experienced member of our laboratory in a blinded manner. For quantitation of the extent of EndMT, staining was performed at each time-point with at least four images evaluated from each of at least three spatially separated thoracic aortic sections per mouse. Data were averaged per animal then used for statistical analyses.

### Detection of hypoxia in atherosclerotic plaques

Hypoxia was detected using pimonidazole hydrochloride (Hypoxyprobe, Hypoxyprobe Inc., Burlington, MA). Mice were injected intraperitoneally with 60 mg kg^−1^ body weight of the pimonidazole agent. One hour later, mice were killed and perfused with 20 ml of 1 × PBS at 4 °C as described above. Aortas were then harvested and mounted as described above. After thawing, 10-μm sections were fixed in cold acetone for 10 min, washed and incubated overnight at 4 °C with rabbit anti-pimonidazole antisera diluted 1:20 in PBS containing 3% BSA. After further washing, sections were labelled with DAPI and imaged by confocal microscopy as described.

### Detection of oxidative stress and apoptosis in murine atherosclerotic plaques

Oxidative stress in murine atherosclerotic plaques was determined by assessing vascular superoxide production *in situ* using dihydroethidium (DHE) fluorescence microscopy. DHE is blue fluorescent (absorption/emission: 355/420 nm) in cell cytoplasm while the oxidized form ethidium is red fluorescent (absorption/emission: 518/605 nm) on DNA intercalation. Aortas were harvested without fixation, and then mounted and sectioned as described above. Next, 10 μmol l^−1^ DHE (D23107; Invitrogen), was applied to each tissue section and sections were incubated in a dark humidified chamber at 37 °C for 30 min. Due to the staining protocols, specific staining could only be performed using DHE (seen in red) and DAPI (blue). Sections were imaged using a confocal microscope (SP5 DM; Leica).

For detection of apoptosis, mouse thoracic aortas underwent perfusion-fixation as described above and apoptotic cells were detected by immunofluorescence using an *In Situ* Cell Death Detection Kit (TUNEL assay; #12156792910910, Roche, Indianapolis, IN). Slides were scanned using a confocal microscope (LSM 510 Meta; Zeiss).

### Flow cytometry and sorting

For FACS-based characterization of circulating cells, peripheral blood was obtained from end.*SclCreER*^T^;*R26RstopYfp*, end.*SclCreER*^T^;*R26RstopYfp*;*ApoE*^*−/−*^ and *Tie2Cre;R26RstopYfp* mice. Erythrocyte lysis was performed using ACK lysing buffer (Invitrogen). Cells were then washed with RPMI 1640 media (Fischer Scientific, Pittsburgh, PA) containing 0.1% BSA and stained with CD45-APC (#559864; BD Biosciences, 1:100) or isotype control (#553991; BD Biosciences, 1:100) as indicated. After washing with 0.1% BSA in RPMI 1640 media, cells were analysed using a FACS LSRII flow cytometer (BD Biosciences).

For FACS of mouse aortic cells, preparations of digested thoracic aortic tissue were obtained from end.*SclCreER*^T^;*R26RstopYfp*;*ApoE*^*−/−*^ mice and end.*SclCreER*^T^;*R26RstopYfp* mice as indicated. Aortas were digested with 2 mg ml^−1^ collagenase (C5138; Sigma) and 0.4 mg ml^−1^ elastase (E7885; Sigma) in RPMI 1640 media at 37 °C for 1 h. The digestion reaction was stopped by adding 20% fetal bovine serum (FBS) by volume of digestion solution. Cells were then filtered and washed with PBS containing 10% FBS. From this point forward, due to the tendency of the Yfp protein to dissipate after fixation and permeabilization, differing protocols were used to detect cell surface versus intracellular proteins in conjunction with Yfp. For FACS of Yfp with Fap, after resuspending cells in PBS with 10% FBS, 7-AAD (Beckman Coulter, Brea, CA) was added and live, viable cells were sorted into Yfp positive and Yfp negative populations using a BD InfluxTM cell sorter (BD Biosciences). Following sorting, cells were fixed in ice-cold 4% paraformaldehyde for 10 min, washed with PBS and blocked with Mouse BD Fc BlockTM (#553141; BD Biosciences) for 15 min. Cells were then stained with anti-Fap-Alexa Fluor 647 (bs-5760R-A647; Bioss, Woburn, MA) in PBS containing 0.5% Saponin for 2 h on ice. Cells were washed with PBS before flow cytometric analysis was performed on a BD LSR II Flow Cytometer. For FACS of Yfp with cell surface-expressed CD31, live cells were incubated for 30 min on ice with Hoechst 33342 (H21492; Invitrogen) and anti-CD31-PECy7 (#25-0311-82; eBiosciences). After washing, 7-AAD (Beckman Coulter, Brea, CA) was added, and live, viable cells positive for Yfp and CD31 were quantified using a BD InfluxTM cell sorter (BD Biosciences).

### Human tissue immunostaining

Human abdominal aortic atherosclerotic immunostaining was performed using pre-existing tissue blocks originally made from autopsy samples as previously described and used in other studies^57^ and with the approval of the Institutional Review Board of the Icahn School of Medicine at Mount Sinai (determined to be ‘not human subjects research' due to strict sample de-identification). Plaques were classified into AHA type V or VI lesions according to standard criteria using H&E staining (other plaque types were excluded)^40^. Fibrous cap thickness was measured using a pre-calibrated ocular micrometer during microscopy. For immunofluorescence staining on formalin-fixed, paraffin-embedded human samples, slides were deparaffinized by twice washing for 5 min in Xylene, followed by two washes in 100% ethanol for 2 min each, and a series of sequential 2 min washes each in 90, 80, 70 and 50% ethanol, followed by rinsing in PBS. Proteinase K (S3004; Dako, Denmark) was used according to manufacturer's instructions. Antigen retrieval was performed in a solution of 10 mM sodium citrate, pH 6.0 for 40 min at 95 °C. After cooling, samples were blocked for 1 h in 5% BSA and 20% donkey serum in PBS, then primary antibodies were applied overnight at 4 °C in 3% BSA as follows: anti-CD31 (ab9498; Abcam, 1:50), anti-CD31 (M0823; Dako, 1:100), anti-FAP (ab28244; Abcam, 1:100), anti-FSP-1 (ab27957; Abcam, 1:100), anti-vWF (A0082; Dako, 1:200), anti-vWF (ab68545; Abcam, 1:100), anti-αSMA (ab5694; Abcam, 1:100), FITC conjugated anti-αSMA (F3777; Sigma, 1:200), anti-SM22α (ab14106; Abcam, 1:100). Slides were then washed and the secondary antibody applied as indicated for 1 h at room temperature (anti-mouse or anti-rabbit conjugated to Alexa Fluor 594 or anti-rabbit conjugated to Alexa Fluor 488, Invitrogen, 1:750). Slides were imaged using a confocal microscope (SP5 DM; Leica). To permit more detailed analyses including linear regression and increase statistical power, we evaluated more samples with the marker combination of vWF with FAP (see Figure legends for sample numbers). For cell counting, images were coded and analysed by an experienced member of our laboratory in a blinded manner.

### Cell culture and induction of EndMT

HUVECs (Lonza, Basel, Switzerland) and HCAECs (Lonza) were cultured in EGM-2 media (Lonza). To induce EndMT, six-well plates were pre-coated with 31.25 ng ml^−1^ fibronectin (F2006; Sigma) for 1 h. HUVECs or HCAECs were initially seeded at 5,000 or 7,500 cells cm^−2^, respectively. Growth medium was changed for complete growth medium containing 50 ng ml^−1^ TGF-β2 (#100-35; Peprotech, Rocky Hill, NJ) with or without hydrogen peroxide (H_2_O_2_) (H1009; Sigma) at 100 or 200 μM (as indicated)^58^ the following morning and every other day for 5 days.

Endothelial cell culture under hypoxic conditions was performed by incubating the cells in an airtight Plexiglass chamber (Billups-Rothenberg Modular Chamber; Del Mar, CA) with an atmosphere of 5% CO_2_/95% N_2_ at 37 °C, as described^59^. Rather than changing growth media every other day, to minimize cell exposure to oxygen the media was replaced only once during the 5-day experiment. Media was also changed only once during the 5-day experiment for control (comparator) cells grown under non-hypoxic conditions.

### Quantitative real-time PCR

RNA was isolated from human cultured cells using Trizol-based methods (Invitrogen). In brief, 1 ml of Trizol was added to each well of a six-well plate. RNA was extracted by phase separation in chloroform. RNA was precipitated by mixing with isopropyl alcohol, and was purified by incremental ethanol washes. RNA was diluted in RNase- and DNase-free water and stored at −80 °C until use. RNA was quantified using a Nanodrop NP-1000 spectrometer (Wilmington, DE). Reverse transcription was performed using the iScript cDNA Synthesis Kit (Bio-Rad, Hercules, CA). Reaction conditions were: 25 °C for 5 min, 42 °C for 30 min, 85 °C for 5 min and 4 °C forever. Subsequently, qRT-PCR was performed using the PerfeCTa SYBR Green FastMix Reaction Mixes kit (vWR, Radnor, PA) according to manufacturer's protocol. *18S* rRNA served as control. Conditions for qRT-PCR were as follows: 95 °C for 5 min, with 40 cycles consisting of 95 °C for 15 s and 60 °C for 1 min; 95 °C for 15 s and 60 °C for 15 s. RNA expression was analysed using the ΔCt method. Primer sequences are provided in Supplementary Table 6.

### Immunoblotting

HCAECs and HUVECs were lysed using Laemmli Sample Buffer (Bio-Rad) mixed with β-mercaptoethanol. After sonication and heating, samples were analysed by western blot (4–20% Novex Tris-Glycine Gel, Invitrogen). Primary antibodies used were as follows: anti-FAP (ab53066, Abcam; 1:500), anti-CD31 (ab9498, Abcam; 1:1,000), anti-active Caspase 3 (ab13847; Abcam, 1:1,000), anti-SM22α (ab14106, Abcam; 1:1,000), anti-phospho-SMAD3 (ab51451, Abcam; 1:1,000), anti-SMAD3 (#9513, Cell Signaling, Danvers, MA; 1:1,000) and anti-glyceraldehyde 3-phosphate dehydrogenase (GAPDH) (G8795, Sigma; 1:10,000). Original uncropped immunoblots from all Figures in this manuscript (Figs 3e,g and 4m,n; Supplementary Figs 9c and 10g) are presented as Supplementary Figs 16–21.

### Immunocytochemistry

Immunostaining of HCAECs and HUVECs was performed under control conditions and following TGF-β2±H_2_O_2_±hypoxia treatment. After fixing with 4% paraformaldehyde for 10 min and washing with PBS, blocking solution (5% normal donkey serum in PBS with 0.1% Triton-X100) was applied for 1 h. The following primary antibodies were applied overnight at 4 °C: anti-active Caspase 3 (ab13847; Abcam, 1:100), anti-SM22α (ab14106; Abcam; 1:100), anti-FAP (ab53066; Abcam; 1:100), anti-CD31 (ab9498; Abcam; 1:50), anti-Ve-Cadherin (sc-6458; Santa Cruz, 1:50). Cells were washed with PBS, and corresponding secondary antibodies were applied for 1 h at room temperature. Nuclear staining was performed through immersion in DAPI (Invitrogen #D3571; 1:1,000). Apoptosis staining of HUVECs was detected using ApopTag *In Situ* Apoptosis Detection Kit (S7100; Millipore, Billerica, MA) according to the manufacturer's instructions.

For staining of human fibroblasts, cultured cells were fixed in methanol at −20 °C for 10 min, washed, and permeabilized in PBS with 0.1% Tween 20 for 10 min at room temperature. After blocking, the following primary antibodies were applied overnight at 4 °C: anti-FAP (ab53066; Abcam; 1:100), anti-Vimentin (ab20346; Abcam, 1:100), anti-FSP-1 (ab27957; Abcam, 1:100). Cells were washed with PBS, and corresponding secondary antibodies were applied for 1 h at room temperature.

Nuclear staining was performed through immersion in DAPI (Invitrogen #D3571; 1:1,000). A minimum of three replicates was performed for every observation.

### *In vitro* cell viability and counting

We initially assessed the number of apoptotic and dead HUVECs using FITC Annexin V Apoptosis Detection kit (BD Pharmingen) according to the manufacturer's instructions. Briefly, HUVEC treated with TGF-β2±H_2_O_2_ for 48 h (consistent with the proliferation assay) were gently trypsinized, washed with cold PBS and resuspended in binding buffer. We then stained 10^6^ cells ml^−1^ with FITC Annexin V and propidium iodide (PI) at room temperature for 15 min. The number of apoptotic and dead cells was estimated by flow cytometry using a FACS LSRII flow cytometer equipped with Diva software (BD Biosciences). However, because this assay required trypsinization, we were concerned this may cause apoptotic cells to die with a consequent mis-representation of cell apoptosis. Therefore only cell death was quantitated by this method. Apoptosis was assessed by western blot and immunostaining for apoptosis-associated proteins and TUNEL assay (ApopTag *In Situ* Apoptosis Detection Kit—see above). Cell counting was performed after 5 days of treatment with TGF-β2±H_2_O_2_ following gentle trypsinization and using a hemocytometer.

### Proliferation assay

Proliferation was measured using a Cell Proliferation ELISA, BrdU (colorimetric) assay kit (Roche) according to the manufacturer's instructions. Briefly, HUVECs were seeded in a 96-well plate. The following morning, media was changed for complete growth medium containing 50 ng ml TGF-β2±H_2_O_2_. Eight hours later, BrdU was added to each well for a further 16 h. Cells were then fixed and anti-BrdU-POD was added to each well to detect the immune complex by substrate reaction. The reaction product was quantified by measuring absorbance at 370 nm using a SpectraMax M5 (Molecular Devices, Sunnyvale, CA). Three wells per experiment were analysed, and three experiments were performed.

### Migration and invasion assays

Cell migration was assessed using a 24-well QCM Chemotaxis cell migration assay (Millipore) with 8 μm pore size, as per the manufacturer's instructions. HUVECs were cultured in basal media or with 50 ng ml^−1^ TGF-β2 (#100-35; Peprotech) and 200 μM H_2_O_2_ as described for 5 days. Cells were resuspended in EBM-2 serum-free medium, counted and plated onto the chemotaxis insert at 37,500 cells per chamber in triplicate. Cells were allowed to migrate overnight towards EBM-2 media+10% FBS at 37 °C. Cells were stained for 20 min at room temperature, washed in distilled water and non-migrated cells remaining on the upper chamber of the insert were scraped off using a cotton swab. Migrated cells were then dissolved in extraction buffer. Cell solution was transferred to a 96-well plate and the optical density was read at 560 nm. Absorbance values were normalized to the average of the unstimulated samples. Migration was calculated from three independent experiments.

Cell invasion was assessed using 24-well, 8 μM polycarbonate Transwell inserts (Corning Costar Inc., Tewksbury, MA). Inserts were coated with growth factor-reduced Matrigel at a concentration of 1 mg ml^−1^ for 1 h at room temperature. After induction of EndMT for 5 days with TGF-β and 200 μM H_2_O_2_ as described, unstimulated and treated cells were resuspended in EBM-2 serum-free medium, counted and plated onto inserts at 15,000 cells per insert in duplicate. After 24 h, invading cells were washed in PBS and fixed in ice-cold 70% ethanol for 10 min. Non-invaded cells were scraped from the upper chamber of the insert with a cotton swab and inserts were washed again in PBS. Membranes were removed from the insert with a scalpel blade and mounted onto a glass slide using Vectashield containing DAPI (Vector Laboratories). Cells were imaged in five random fields per membrane at × 10 magnification and counted using ImageJ software (National Institutes of Health, Bethesda, MD). Cell invasion was calculated from three independent experiments.

### MMP activity and protein assays

After standard induction of EndMT with TGF-β and 200 μM H_2_O_2_ in HUVECs or culture of human fibroblasts as described above and then careful washing, EBM-2+0.2% FBS was applied to the cells for 16 h. Conditioned media was collected and cells were lysed in RIPA buffer and samples stored at −80 °C pending analysis. MMP activity was measured using a SensoLyte 520 Generic MMP Fluorimetric Assay Kit (AS-71158; Anaspec, Fremont, CA) according to the manufacturer's instructions. Conditioned media was applied undiluted and cell lysates were diluted in assay buffer to a concentration of 200 ng ml^−1^. EBM-2+0.2% FBS was used as negative control. The final reaction was measured using a SpectraMax M5 with an excitation of 490 nm and emission detected at 520 nm (Molecular Devices, Sunnyvale, CA).

Specific MMP and TIMP protein levels were measured using the Quantibody Human MMP Array 1 (QAH-MMP-1; RayBiotech, Norcross, GA) for the detection of MMP1, 2, 3, 8, 9, 10, 13 and TIMP1, 2 and 4. Undiluted conditioned media was applied to the array as per the manufacturer's instruction, and EBM-2+0.2% FBS used as a negative control. The array was scanned using an Axon GenePix laser scanner (Molecular Devices) with a Cy3 wavelength. Densitometry data was acquired using ImageJ software. All samples were normalized to an internal positive control, and MMP/TIMP protein quantification obtained using standards of known concentration.

### Microarray sample preparation

Primary human fibroblasts were obtained from skin biopsy samples from three healthy control subjects enrolled in the CAUSE study (ClinicalTrials.Gov Identifier: NCT01808729). This study was approved by the Institutional Review Board of the Icahn School of Medicine at Mount Sinai, and all subjects provided written informed consent. Freshly obtained skin biopsy samples were washed, disrupted, plated in DMEM/F-12 media (#11330-032; Gibco, Grand Island, NY) with 20% FBS and incubated at 37 °C under a cover slip. After ∼4 weeks, fibroblasts were detached and passaged. All fibroblast lines were then uniformly passaged and used for final experiments at<P7. Prior to harvesting for RNA extraction, fibroblasts were switched to DMEM/F-12 media with 5% FBS for 24 h to replicate HUVEC culture conditions (EGM-2 media contains 5% FBS).

HUVECs were grown as described. RNA was isolated from both HUVECs and fibroblasts using the RNeasy mini Kit (Qiagen, Germantown, MD) according to the manufacturer's instructions. Residual DNA was removed using the RNase-Free DNase Set (Qiagen) on-column DNase treatment during RNeasy preparation. RNA was amplified by Illumina TotalPrep RNA Amplification Kit and hybridized to the HumanHT-12 v4 Expression BeadChip (Illumina, San Diego, CA). This chip was scanned using an Illumina HiScan.

### Statistical and bioinformatics analyses

For cell culture, each individual experiment was repeated at least three times. Where appropriate, mice were randomized into experimental groups by random number generator. Statistical power was determined from prior studies from our laboratories using similar models^17^,^56^. Data were normalized, as appropriate, to means of each experiment with the reference condition set at 1. Experimental data were analysed by unpaired two-tailed *t*-test, one-way analysis of variance with *post hoc* Dunnett's (with baseline as the reference) or Newman–Keuls (if multiple inter-group comparisons were appropriate) multiple comparison tests, or two-way analysis of variance with Bonferroni posttests as indicated. Results are expressed as mean±s.e.m. Linear regression was used to model the relationship between fibrous plaque cap thickness and % FAP^+^vWF^+^ co-positive cells in human atherosclerotic plaques. Differences were deemed significant when *P*<0.05. These analyses were performed using Prism, Version 4.00 (GraphPad Software) or STAT VIEW software package (SAS, Cary, NC).

For microarray data analysis, images generated by the Illumina Hiscan were uploaded into GenomeStudio Software for primary data extraction and analysis. Spurious probes were filtered by translating the detection *P* values into *q* values^60^,^61^ and removing any probe with maximum *q* value across all samples ⩾10%. Differential expression analysis of the resulting log_2_-transformed data was performed with significance analysis of microarrays using its default parameters^33^,^34^, and genes assigned a false discovery rate <5% were considered differentially expressed. Enriched pathways and other gene sets, such as extracellular matrix organization genes, were identified in the resultant lists of differentially expressed genes using the ingenuity pathway analysis (Ingenuity Systems, Redwood City, CA) and molecular signature database^62^ applications, respectively (with a false discovery rate <5% indicating enrichment). Clustering of gene expression data was performed using the group-average hierarchical clustering algorithm with Pearson's correlation as the similarity metric. This computation and visualization of the resultant dendrogram was done using Matlab (Mathworks, Natick, MA), along with additional verification of the robustness of these results using single-linkage algorithm, Euclidean distance and other options for hierarchical clustering. Principal component analysis on the expression profiles across all cell types of the EndMT-related genes (Supplementary Table 2) was also performed and the results visualized in Matlab. Microarray data are deposited in GEO, accession number: GSE56309.

### Data availability

Microarray data that support the findings of this study have been deposited in NCBI's Gene Expression Omnibus (GEO), and are accessible through GEO series accession number GSE56309. Original blots used for protein quantification supporting the findings of this study are available within the article (and its Supplementary Information Files). Other data that support the findings of this study are available from the corresponding author upon request.

## Additional information

**How to cite this article:** Evrard, S.M. *et al*. Endothelial to mesenchymal transition is common in atherosclerotic lesions and is associated with plaque instability. *Nat. Commun.* 7:11853 doi: 10.1038/ncomms11853 (2016).

## Supplementary Material

Supplementary Information Supplementary Figures 1-21, Supplementary Tables 1-6 and Supplementary References

Supplementary Movie 1 Endothelial lineage-derived Yfp^+^ cells undergo EndMT and give rise to Fap^+^ fibroblast-like cells within intimal plaques. Immunofluorescence confocal microscopy with z-stack acquisition of a thoracic aortic section from a tamoxifen-induced end. *SclCreER^T^;R26RstopYfp;ApoE^-/-^* mice fed with 30 weeks of HFD revealed intimal plaque Yfp^+^ cells co-expressing the fibroblast marker Fap. Movie corresponds to the same section as displayed in Fig. 2c.

Supplementary Movie 2 Endothelial lineage-derived Yfp^+^ cells undergo EndMT and give rise to Fsp-1^+^ adventitial fibroblast-like cells. Immunofluorescence confocal microscopy with z-stack acquisition of a thoracic aortic section from a tamoxifen-induced end. *SclCreER^T^;R26RstopYfp;ApoE^-/-^* mice fed with 8 weeks of HFD revealed adventitial Yfp^+^ cells co-expressing the fibroblast marker Fsp-1. Movie corresponds to the same section as displayed in Supplementary Fig. 5h.

## Figures and Tables

**Figure 7:**