#  Corrigendum: Endothelial to mesenchymal transition is common in atherosclerotic lesions and is associated with plaque instability

**DOI:** 10.1038/ncomms14710

**Published:** 2017-02-16

**Authors:** Solene M. Evrard, Laura Lecce, Katherine C. Michelis, Aya Nomura-Kitabayashi, Gaurav Pandey, K-Raman Purushothaman, Valentina d'Escamard, Jennifer R. Li, Lahouaria Hadri, Kenji Fujitani, Pedro R. Moreno, Ludovic Benard, Pauline Rimmele, Ariella Cohain, Brigham Mecham, Gwendalyn J. Randolph, Elizabeth G. Nabel, Roger Hajjar, Valentin Fuster, Manfred Boehm, Jason C. Kovacic

Nature Communications 8: Article number: 11853 ; DOI: 10.1038/ncomms11853 (2016); Published: 06 24 2016; Updated: 02 16 2017

In this Article, the catalogue number for the anti-Fap-Alexa Fluor 647 antibody is listed incorrectly and should have read bs-5758R-A647 instead of bs-5760R-A647.

