# Supplementary material for: Endothelial to mesenchymal transition is common in atherosclerotic lesions and is associated with plaque instability
Source: Nat Commun. 2016 Jun 24;7:11853. doi: 10.1038/ncomms11853 (PMC4931033; doi:10.1038/ncomms11853)
Supplement: Supplementary Information — Supplementary Figures 1-21, Supplementary Tables 1-6 and Supplementary References [file ncomms11853-s3.pdf]

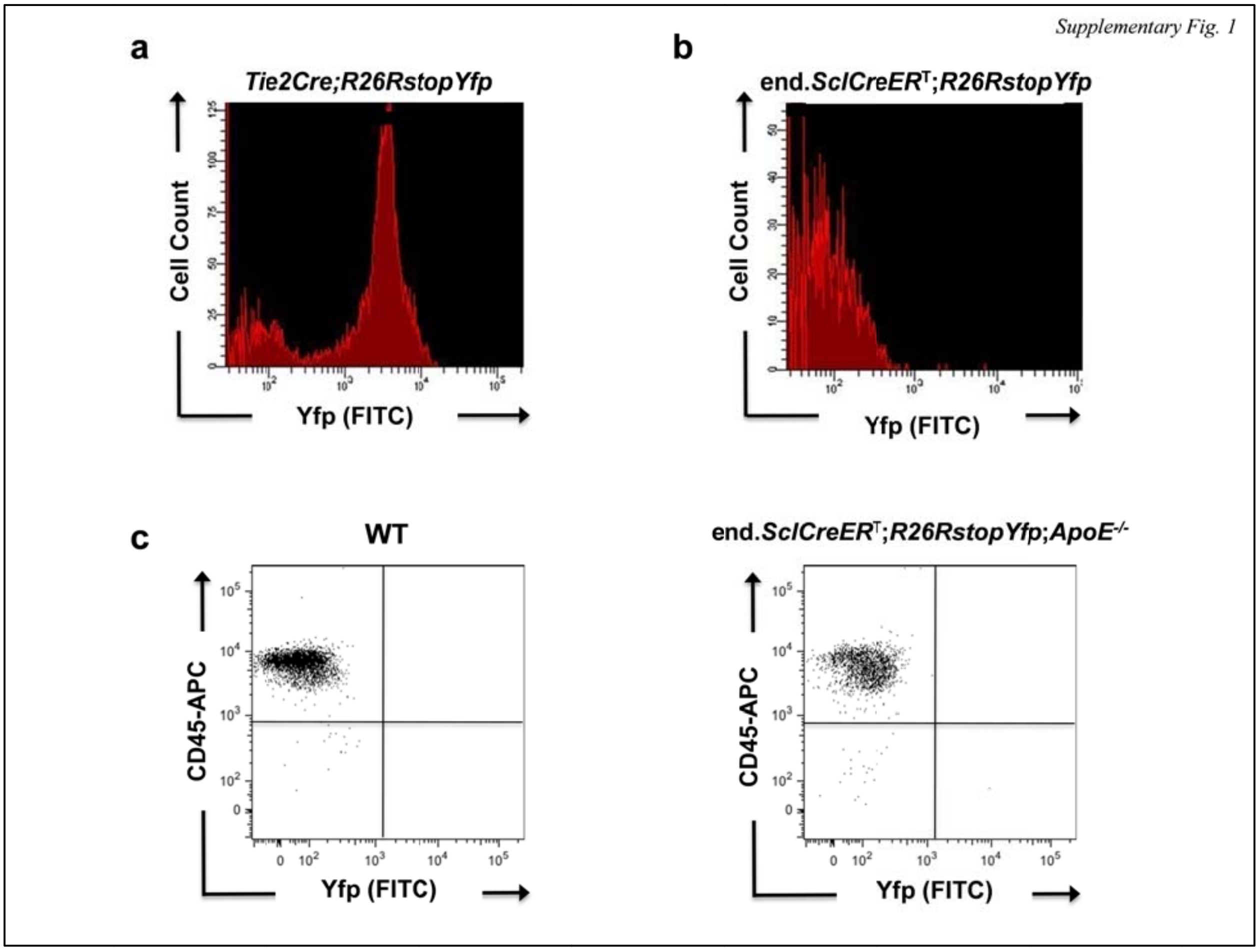

Supplementary Fig. 1. Specificity of endothelial cell fate tracking models by FACS. (a) Representative FACS histogram of peripheral leukocytes from adult *Tie2Cre;R26RstopYfp* mice. Preliminary experiments confirmed that >50% of all peripheral blood leukocytes express Yfp (seen in FITC channel) in this mouse model (not presented). **(b)** Representative FACS histogram of peripheral blood leukocytes from adult tamoxifen-induced end.*SclCreER*^{T};*R26RstopYfp* mice. **(c)** FACS scatter plots of peripheral leukocytes from WT and tamoxifen-induced, HFD-fed end.*SclCreER*^{T};*R26RstopYfp*;*ApoE*$^{-/-}$ mice at 18 weeks of age for CD45 (APC conjugated) and Yfp (detected in FITC channel). Of CD45^{+} cells, the mean percentage of events detected in the FITC channel in end.*SclCreER*^{T};*R26RstopYfp*;*ApoE*$^{-/-}$ mice at 18 weeks of age was 0.09 ± 0.05 % (n = 4), which did not differ from WT control mice (0.07 ± 0.07; n = 3; p = 0.76 for WT versus end.*SclCreER*^{T};*R26RstopYfp*;*ApoE*$^{-/-}$).

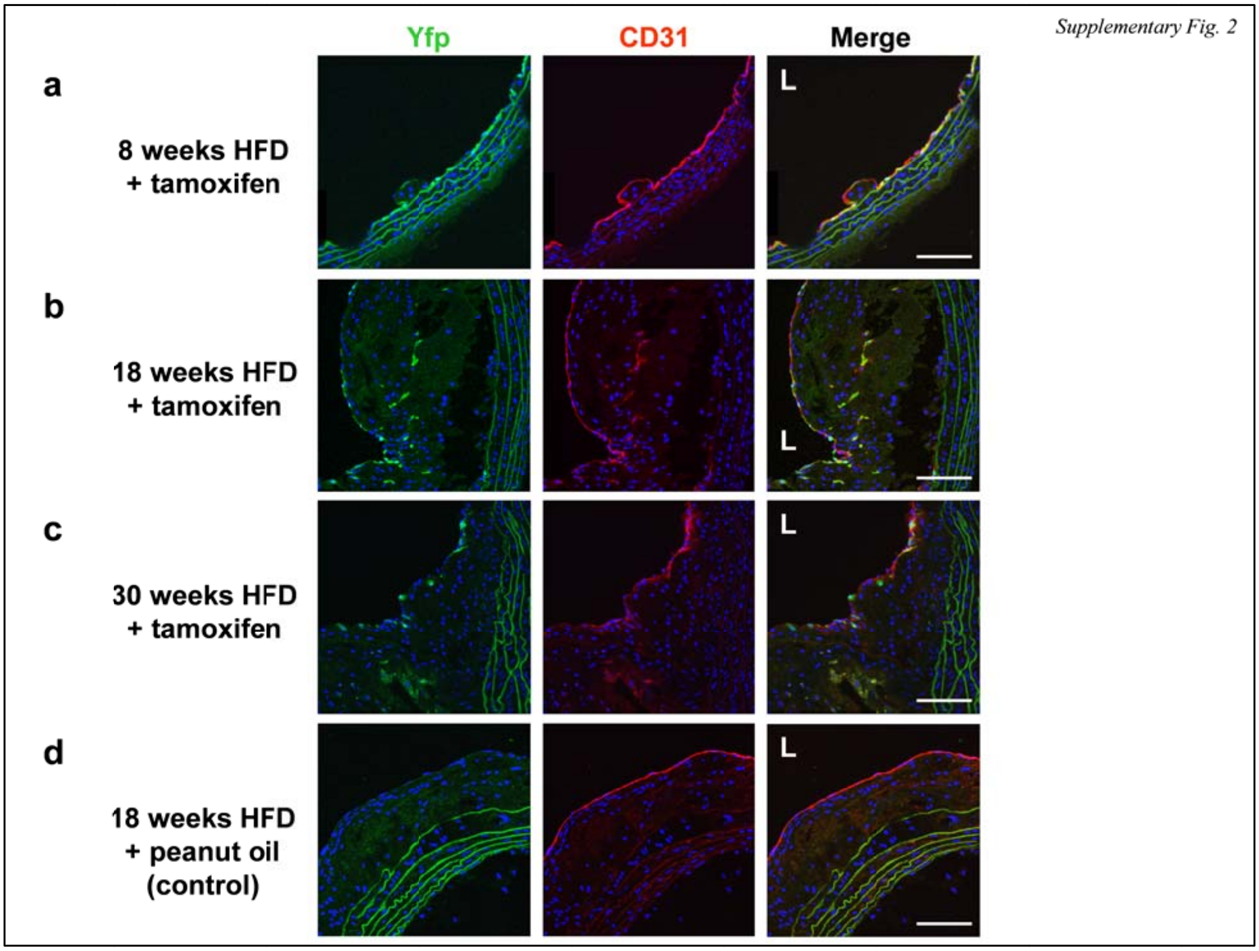

Supplementary Fig. 2. Sensitivity and specificity of end.*SclCreER*^{T};*R26RstopYfp*;*ApoE*$^{-/-}$ endothelial cell fate tracking model. (a - c) Immunofluoresence staining for Yfp and CD31 performed on thoracic aortic sections from tamoxifen-induced end.*SclCreERT;R26RstopYfp;ApoE*$^{-/-}$ mice after 8, 18 or 30 weeks of HFD (corresponding to 14, 24 or 36 weeks of age). L = lumen; scale bars represent 100 µm. **(d)** Immunofluoresence staining for Yfp and CD31, acquired using identical microscope settings and staining protocol, performed on thoracic aortic sections from a control mouse that received peanut oil and after 18 weeks of HFD. L = lumen; scale bars represent 100 µm.

Supplementary Fig. 2

e

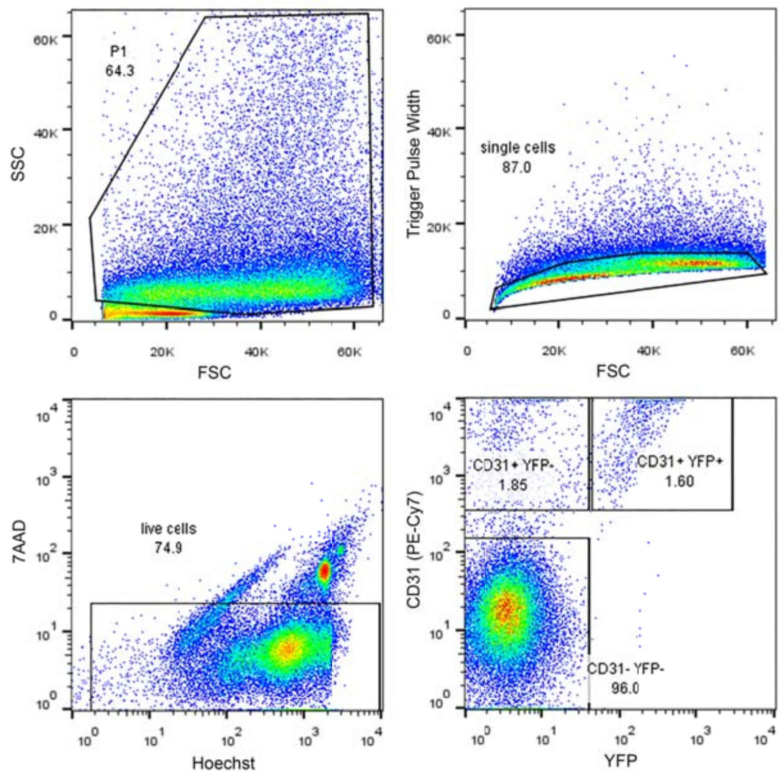

Supplementary Fig. 2 (continued). (e) Representative FACS plots demonstrating the gating scheme and results for the determination of the proportion of $CD31^+$ cells expressing Yfp in end.*SclCreERT;R26RstopYfp;ApoE$^{-/-}$* mice after 8 weeks of HFD. Cell selection was first based on forward (FSC) and side scatter (SSC) (upper left panel), then we selected the single cell fraction (upper right panel), then live cells by gating for Hoechst 33342 positive and 7AAD negative cells (lower left panel). Following this selection, the lower right panel shows the percentage of $CD31^+$ cells labeled with anti-CD31 PE-Cy7 antibody and the proportion of $CD31^+$ cells expressing Yfp.

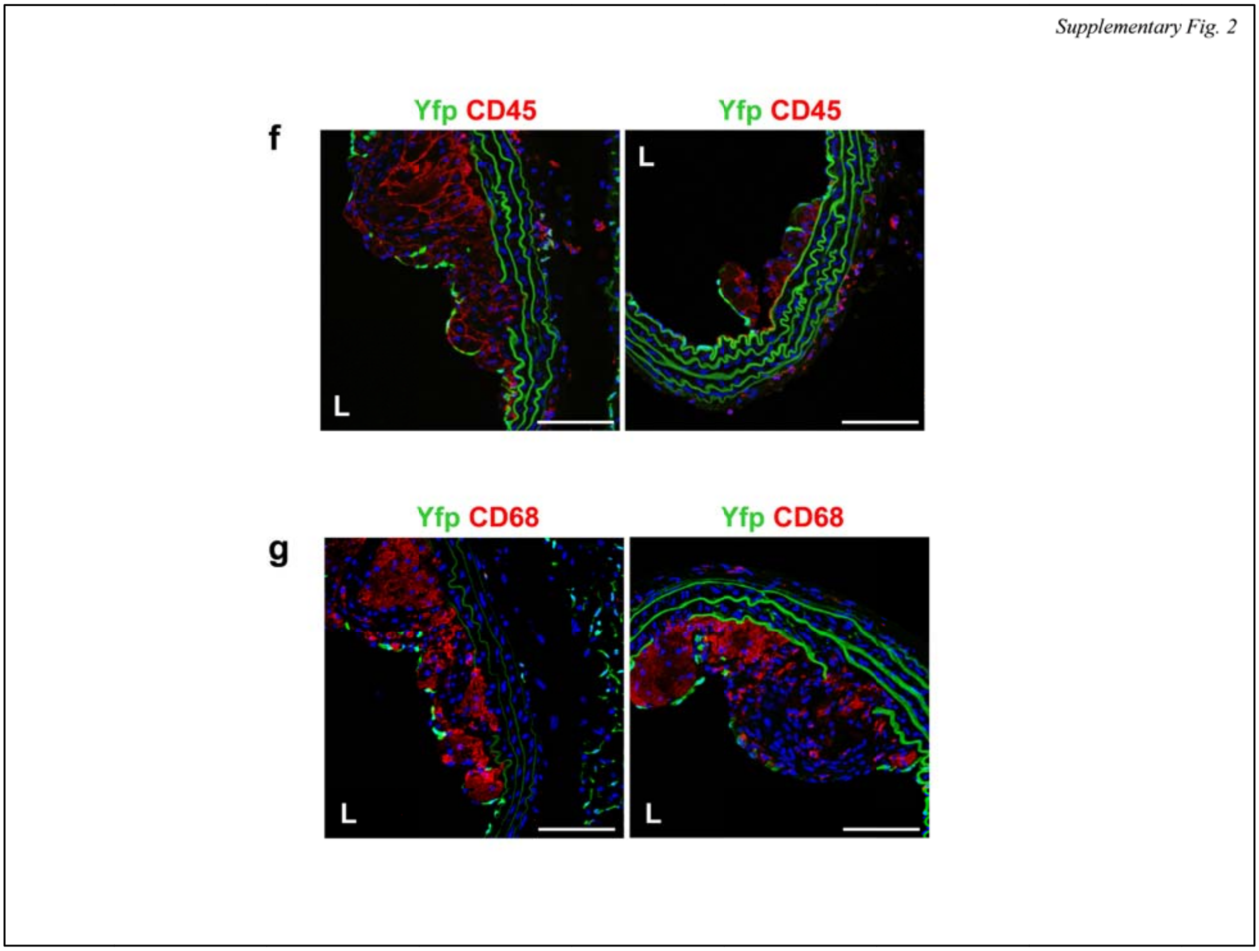

Supplementary Fig. 2 (continued). (f) Immunofluoresence staining for Yfp and CD45, or **(g)** Yfp and CD68, did not reveal any co-positive cells at any time-point. Shown here is staining of mice after 18 weeks of HFD. L = lumen; scale bars represent 100 µm.

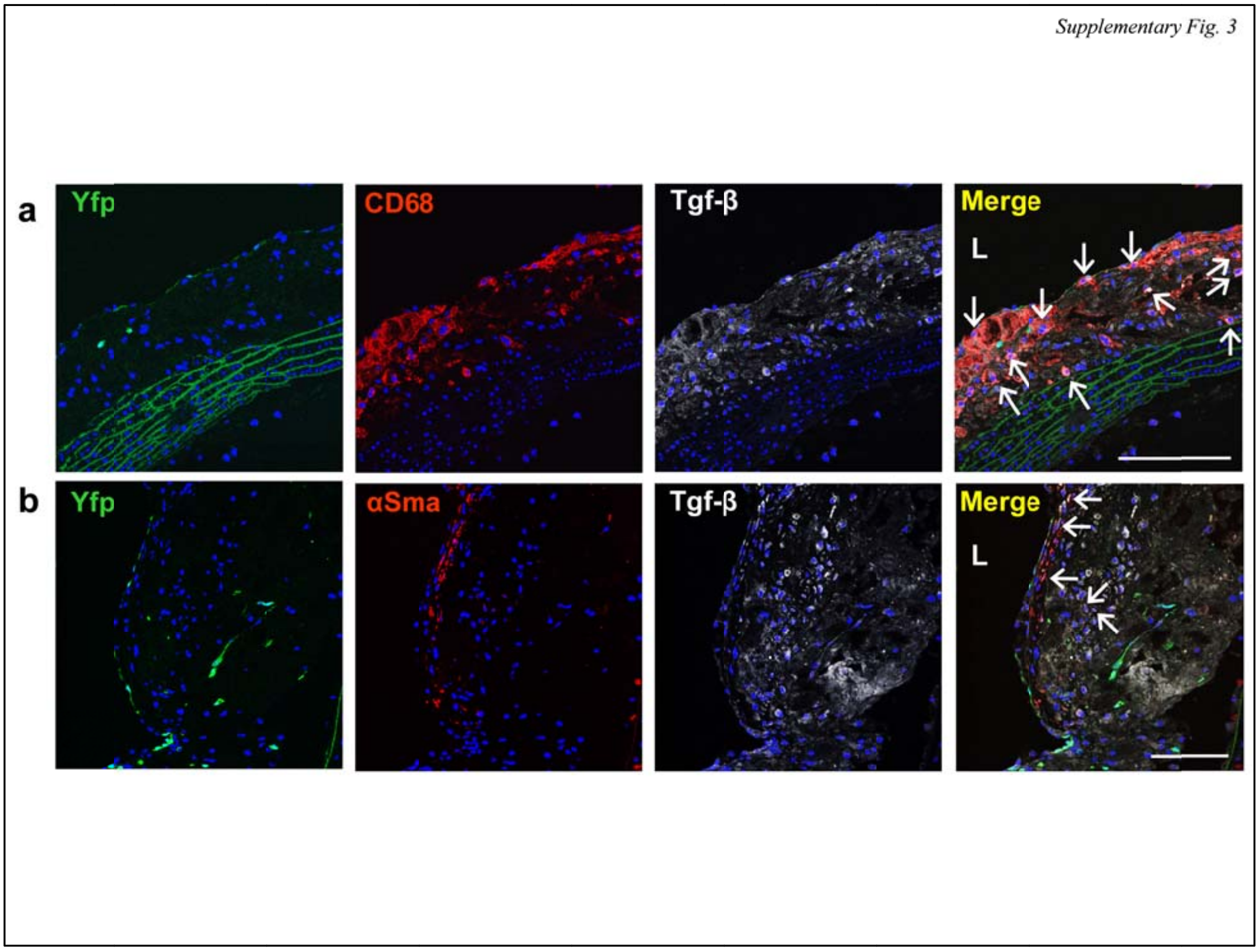

Supplementary Fig. 3. Tgf-β expression in atherosclerotic plaques from end.*SclCreER*^{T};*R26RstopYfp*;*ApoE*$^{-/-}$ mice. (a) Immunofluorescence staining showing Tgf-β expression by $CD68^{+}$ macrophages in atherosclerotic plaque. **(b)** Immunofluorescence staining showing Tgf-β expression by αSma^{+} cells in atherosclerotic plaque. Sections shown are from mice that received 18 weeks of HFD. L = lumen; scale bars represent 100 µm. Arrows indicate co-positive cells.

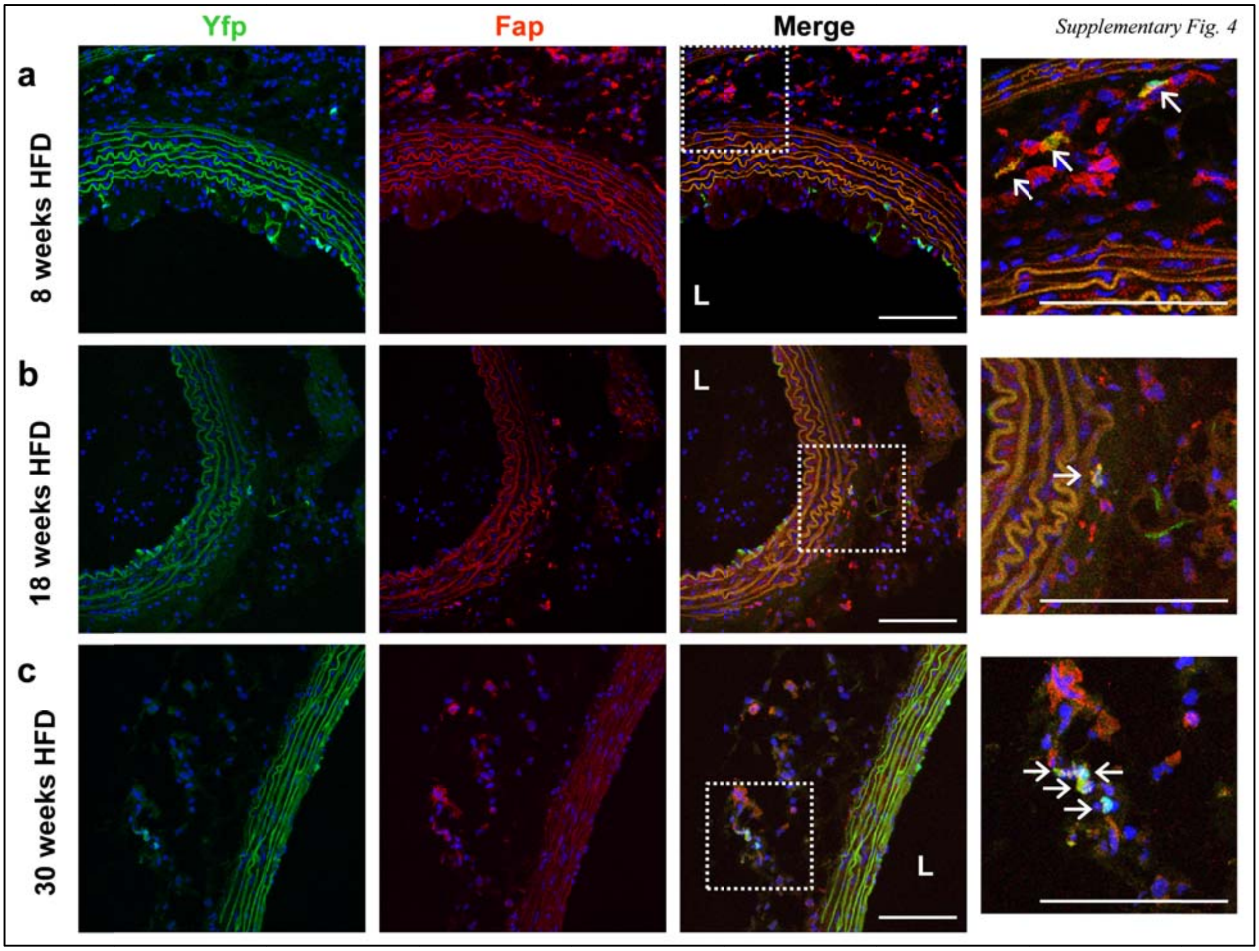

Supplementary Fig. 4. Endothelial lineage-derived cells undergo EndMT and give rise to Fap⁺ fibroblast-like cells within the adventitia. (a – c) Immunofluorescence confocal microscopy of thoracic aortic sections from tamoxifen-induced end.*SclCreER*^{T};*R26RstopYfp*;*ApoE*$^{-/-}$ mice fed with **(a)** 8, **(b)** 18 or **(c)** 30 weeks of HFD revealed adventitial Yfp^{+} cells co-expressing the fibroblast-specific marker Fap. L = lumen; scale bars represent 100 µm. Insets are shown at higher magnification as indicated and arrows indicate co-positive cells.

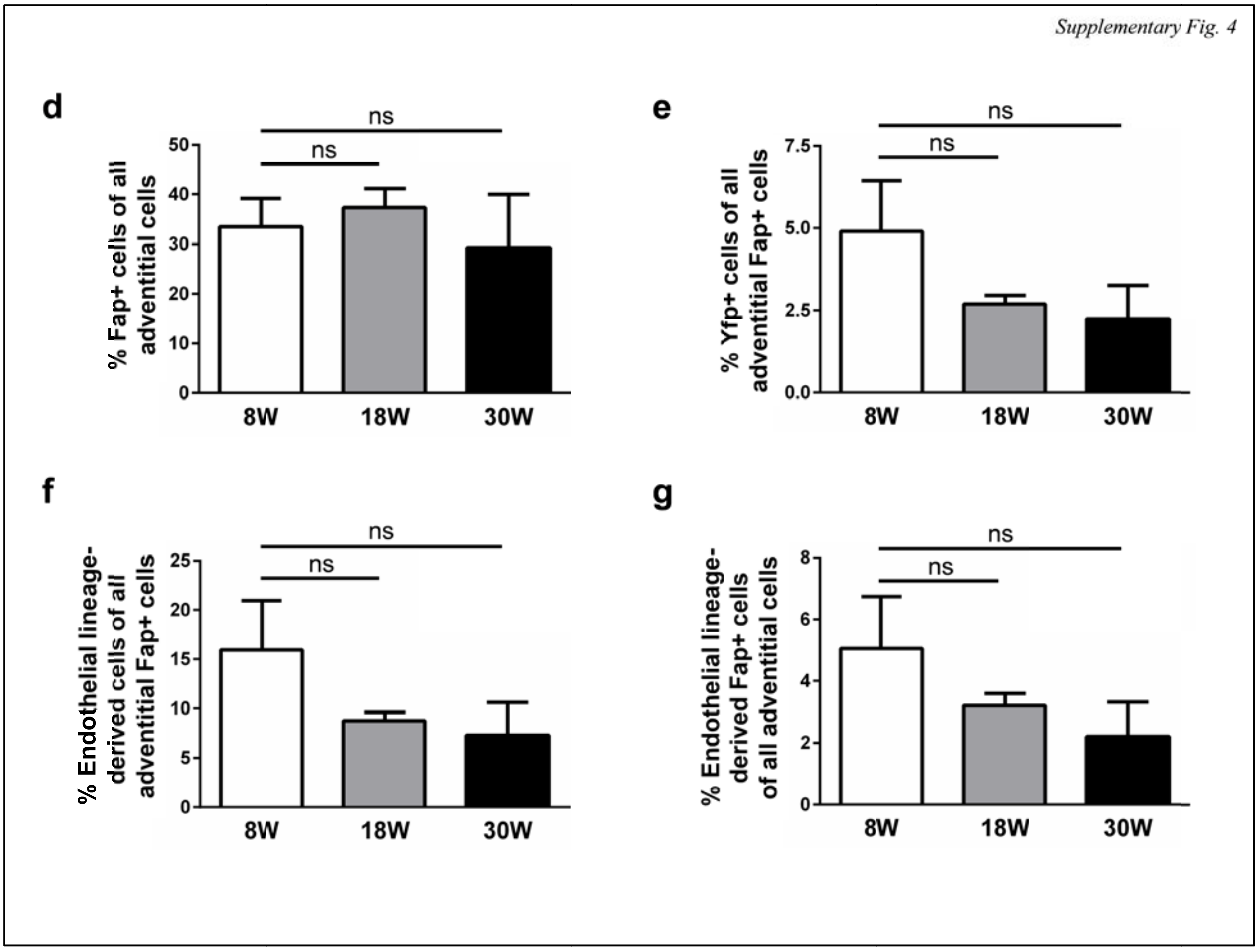

Supplementary Fig. 4 (continued). (d) Quantitation of adventitial cells revealed that after 8, 18 and 30 weeks of HFD 33.4 ± 5.7%, 37.3 ± 3.8% and 29.3 ± 10.7% (respectively) of cells expressed Fap. **(e)** Quantitation (not accounting for the % of endothelial cells expressing Yfp) identified that after the abovementioned periods of HFD 4.9 ± 1.5%, 2.7 ± 0.3% and 2.2 ± 1.0% (respectively) of adventitial Fap^+ cells co-expressed Yfp. **(f)** After taking into account the efficiency of our endothelial lineage tracking system (the % of endothelial cells expressing Yfp), at the same time-points respectively, 15.9 ± 5.0%, 8.8 ± 0.8% and 7.3 ± 3.3% of adventitial Fap^+ cells were derived from endothelial lineage cells. **(g)** Again taking into account the efficiency of our endothelial lineage tracking system, at the same time-points respectively, we determined that 5.1 ± 1.7%, 3.2 ± 0.4% and 2.2 ± 1.1% of all adventitial cells were endothelial lineage-derived Fap^+ cells. Staining was performed at each time-point with at least 4 images evaluated from each of at least 3 spatially separated thoracic aortic sections per mouse (*n* = 5 mice for 8 and 18 weeks; *n* = 4 mice for 30 weeks). Data were averaged per animal then used for statistical analyses. Analysis by 1-way ANOVA.

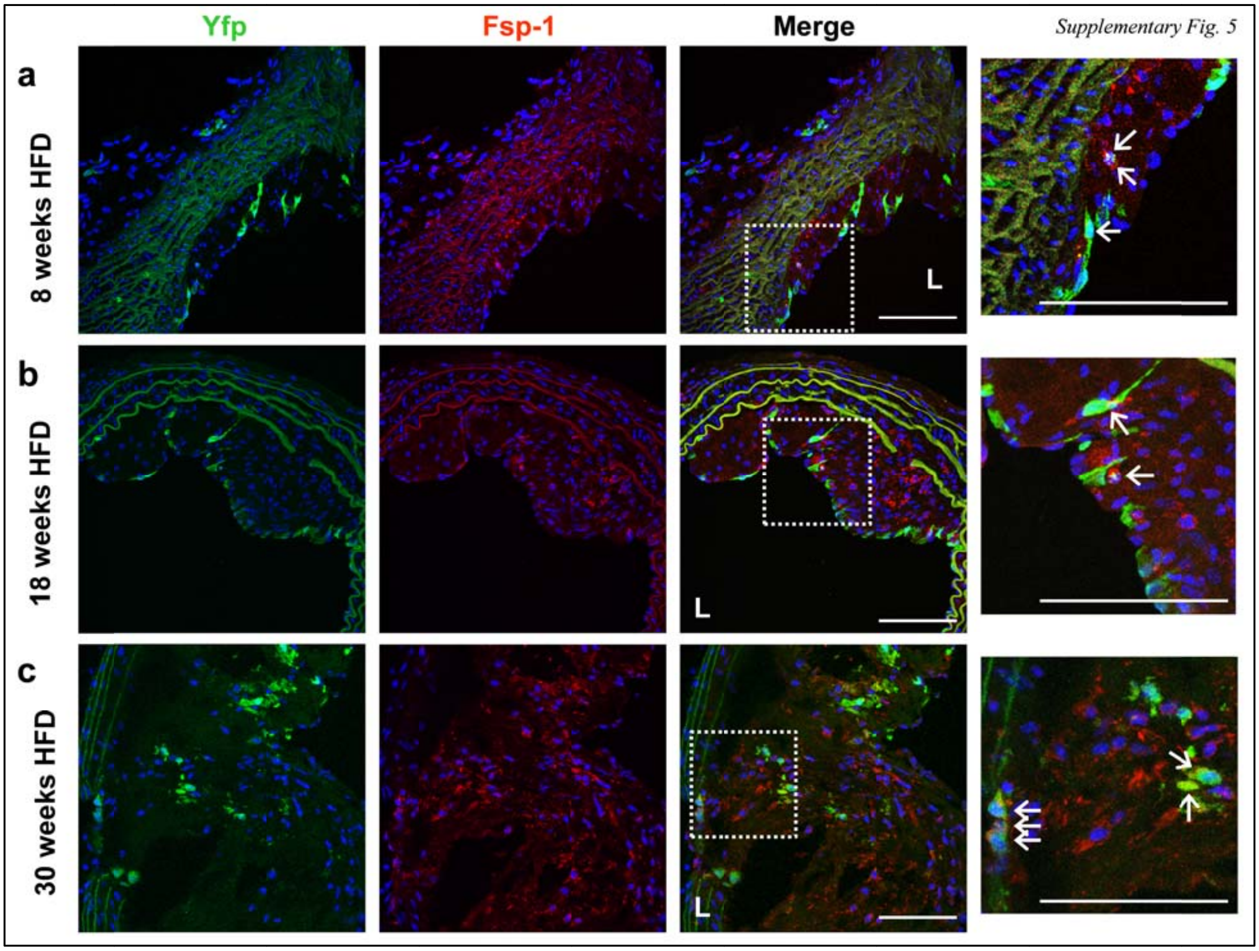

Supplementary Fig. 5. Endothelial lineage-derived cells undergo EndMT and give rise to Fsp-1^{+} fibroblast-like cells within the intima and adventitia. (a – c) Immunofluorescence confocal microscopy of thoracic aortic sections from tamoxifen-induced end.*SclCreER*^{T};*R26RstopYfp*;*ApoE*$^{-/-}$ mice fed with **(a)** 8, **(b)** 18 or **(c)** 30 weeks of HFD revealed Yfp^{+} cells co-expressing the fibroblast marker Fsp-1 within intimal plaques. L = lumen; scale bars represent 100 µm. Insets are shown at higher magnification as indicated and arrows indicate co-positive cells.

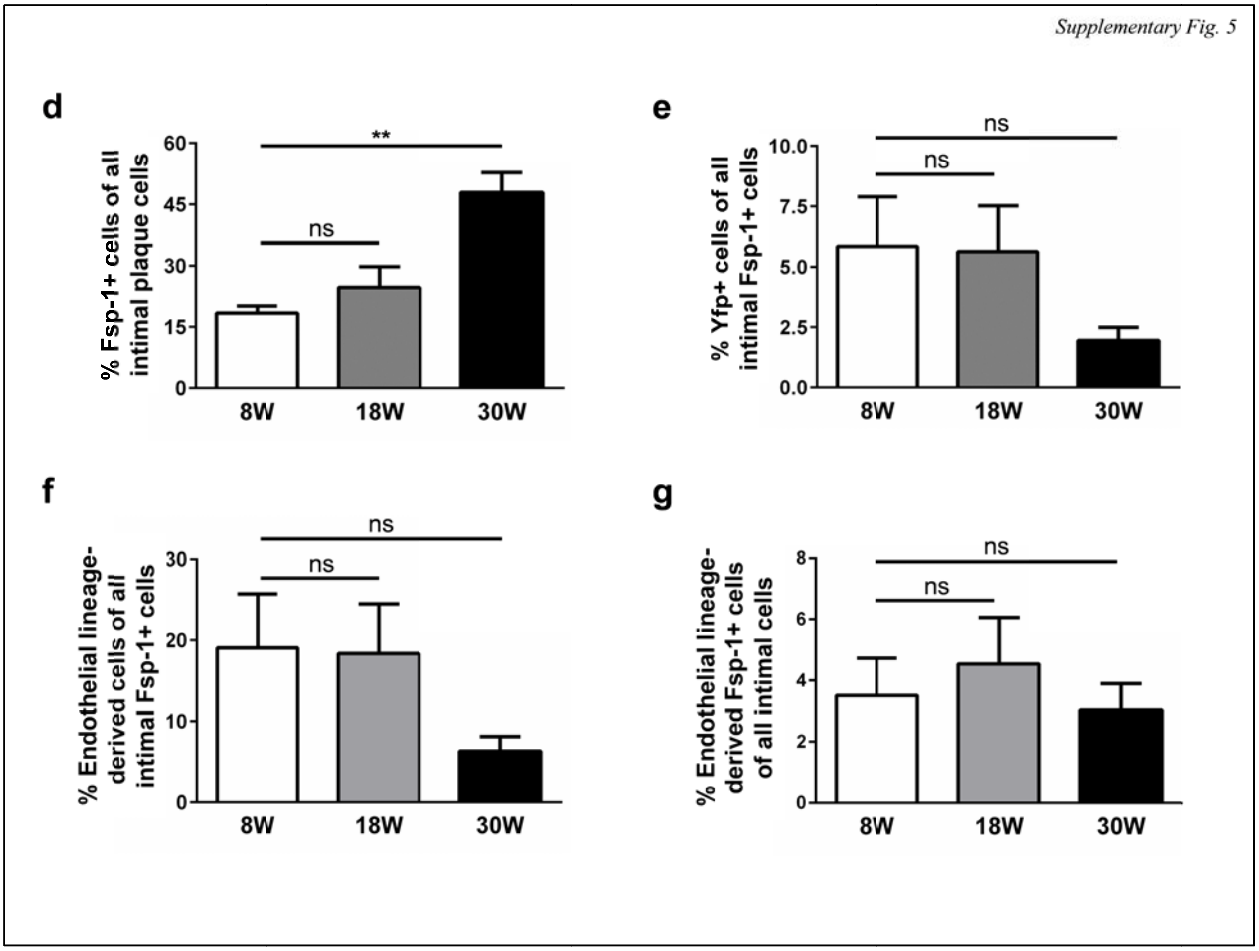

Supplementary Fig. 5 (continued). (d) Quantitation of cells in intimal plaques revealed that after 8, 18 and 30 weeks of HFD 18.4 ± 1.8%, 24.7 ± 5.0% and 47.9 ± 4.9% (respectively) of cells expressed Fsp-1, indicative of a fibroblast phenotype. **(e)** Quantitation (not accounting for the proportion of endothelial cells expressing Yfp) identified that after the abovementioned periods of HFD 5.9 ± 2.0%, 5.6 ± 1.9% and 1.9 ± 0.6% (respectively) of intimal Fsp-1$^+$ cells co-expressed Yfp. **(f)** After taking into account the efficiency of our endothelial lineage tracking system (the % of endothelial cells expressing Yfp), at the same time-points, we determined that 19.1 ± 6.7%, 18.4 ± 6.1% and 6.3 ± 1.8% of intimal Fsp-1$^+$ cells were derived from endothelial lineage cells. **(g)** Again taking into account the efficiency of our endothelial lineage tracking system, at the same time-points respectively, we determined that 3.5 ± 1.2%, 4.5 ± 1.5% and 3.0 ± 0.9% of all intimal cells were endothelial lineage-derived Fsp-1$^+$ cells. Staining was performed at each time-point with at least 4 images evaluated from each of at least 3 spatially separated thoracic aortic sections per mouse (n = 5 mice for 8 and 18 weeks; n = 4 mice for 30 weeks). Data were averaged per animal then used for statistical analyses. Analysis by 1-way ANOVA. $**P < 0.01$.

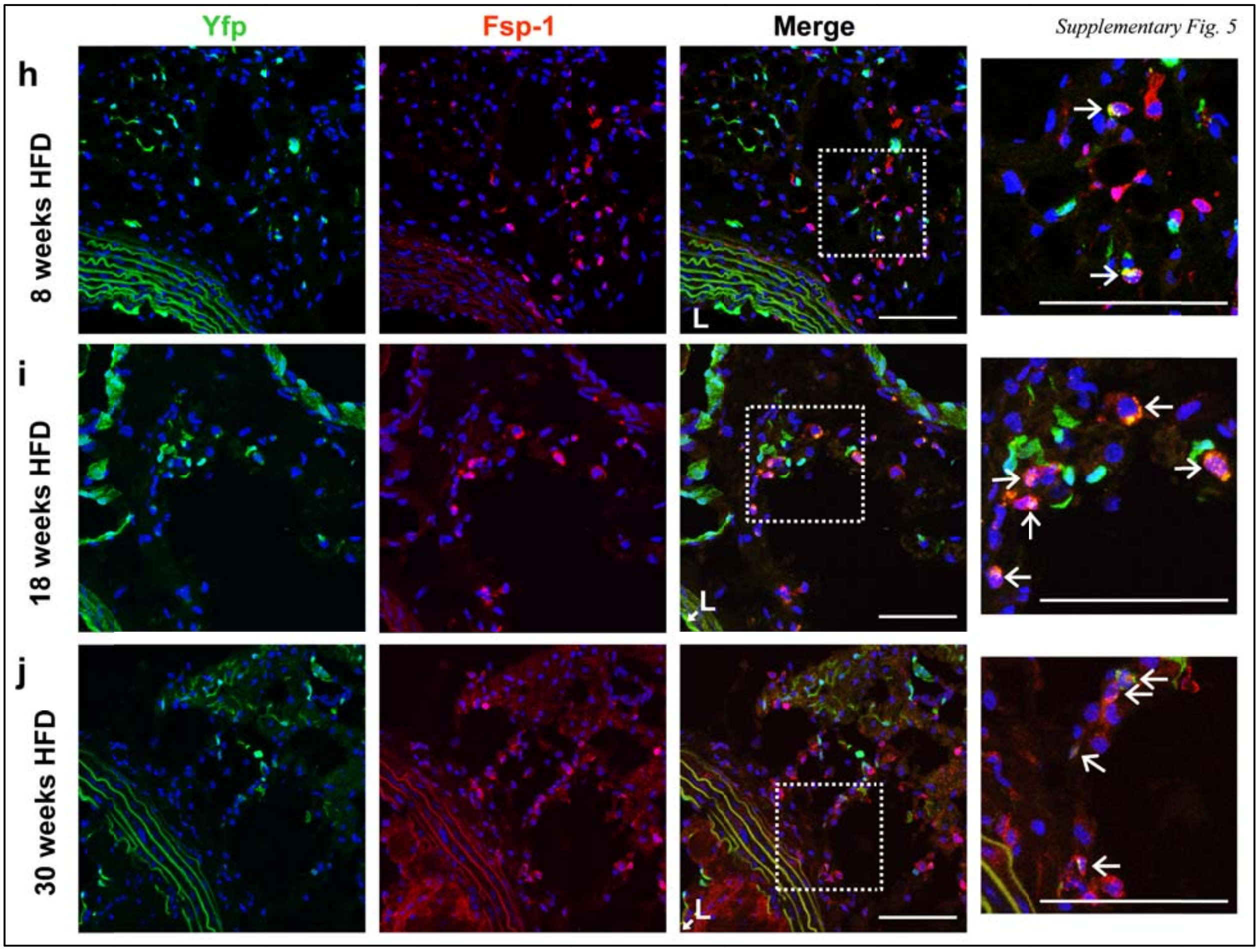

Supplementary Fig. 5 (continued). (h – j) Immunofluorescence confocal microscopy of the adventitia from thoracic aortic sections of tamoxifen-induced end.*SclCreER*^{T};*R26RstopYfp*;*ApoE*$^{-/-}$ mice fed with **(h)** 8, **(i)** 18 or **(j)** 30 weeks of HFD also revealed Yfp^{+} cells co-expressing the fibroblast marker Fsp-1. L = lumen; scale bars represent 100 µm. Insets are shown at higher magnification as indicated and arrows indicate co-positive cells.

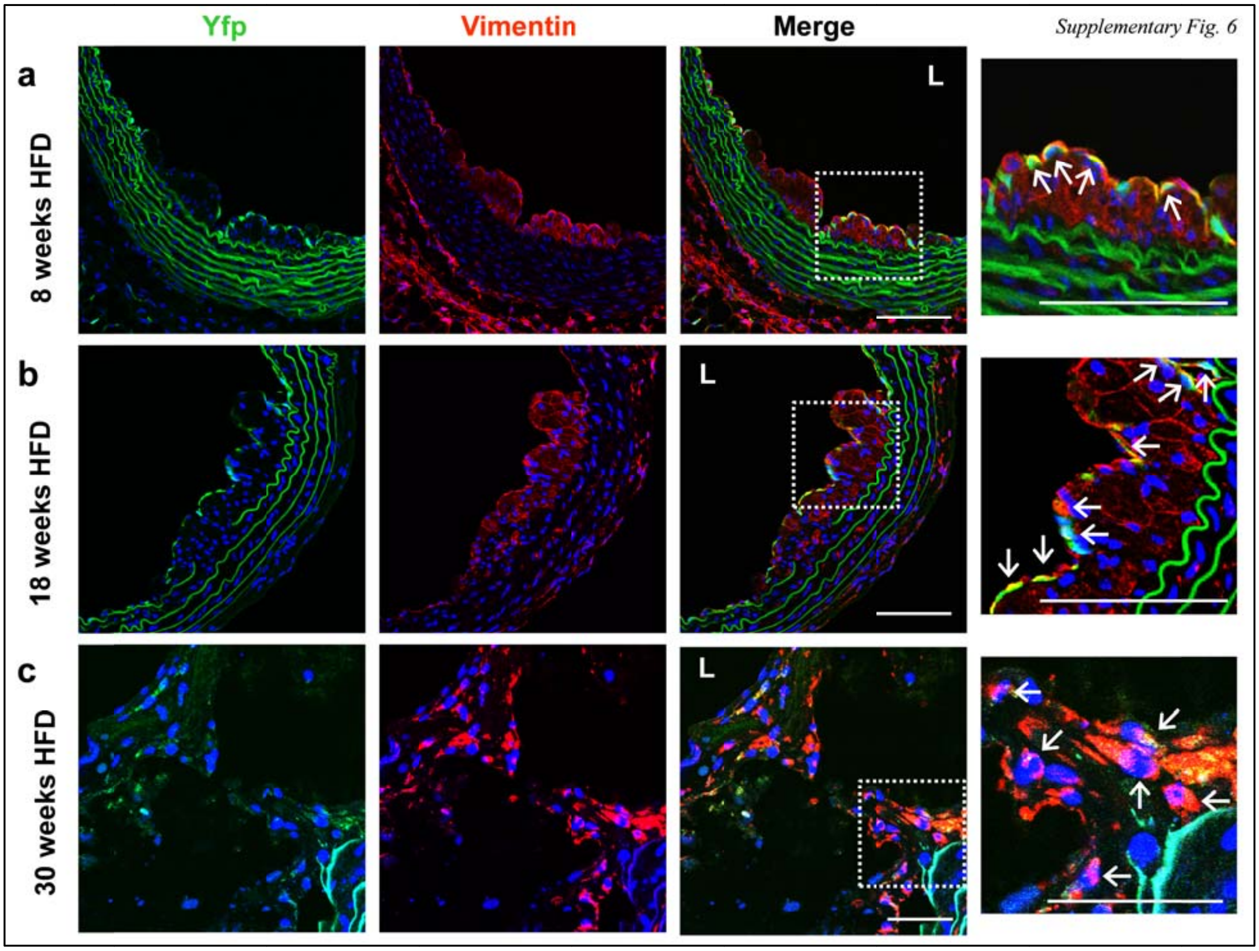

Supplementary Fig. 6. Endothelial lineage-derived cells undergo EndMT and give rise to Vimentin$^+$ fibroblast-like cells within the intima and adventitia. (a – c) Immunofluorescence confocal microscopy of thoracic aortic sections from tamoxifen-induced end.*SclCreER*^{T};*R26RstopYfp*;*ApoE*$^{-/-}$ mice fed with **(a)** 8, **(b)** 18 or **(c)** 30 weeks of HFD revealed Yfp$^+$ cells co-expressing the fibroblast marker Vimentin within intimal plaques. L = lumen; scale bars represent 100 µm for Supplementary Figs. 6a and b, and 50 µm for Supplementary Fig. 6c. Insets are shown at higher magnification as indicated and arrows indicate co-positive cells.

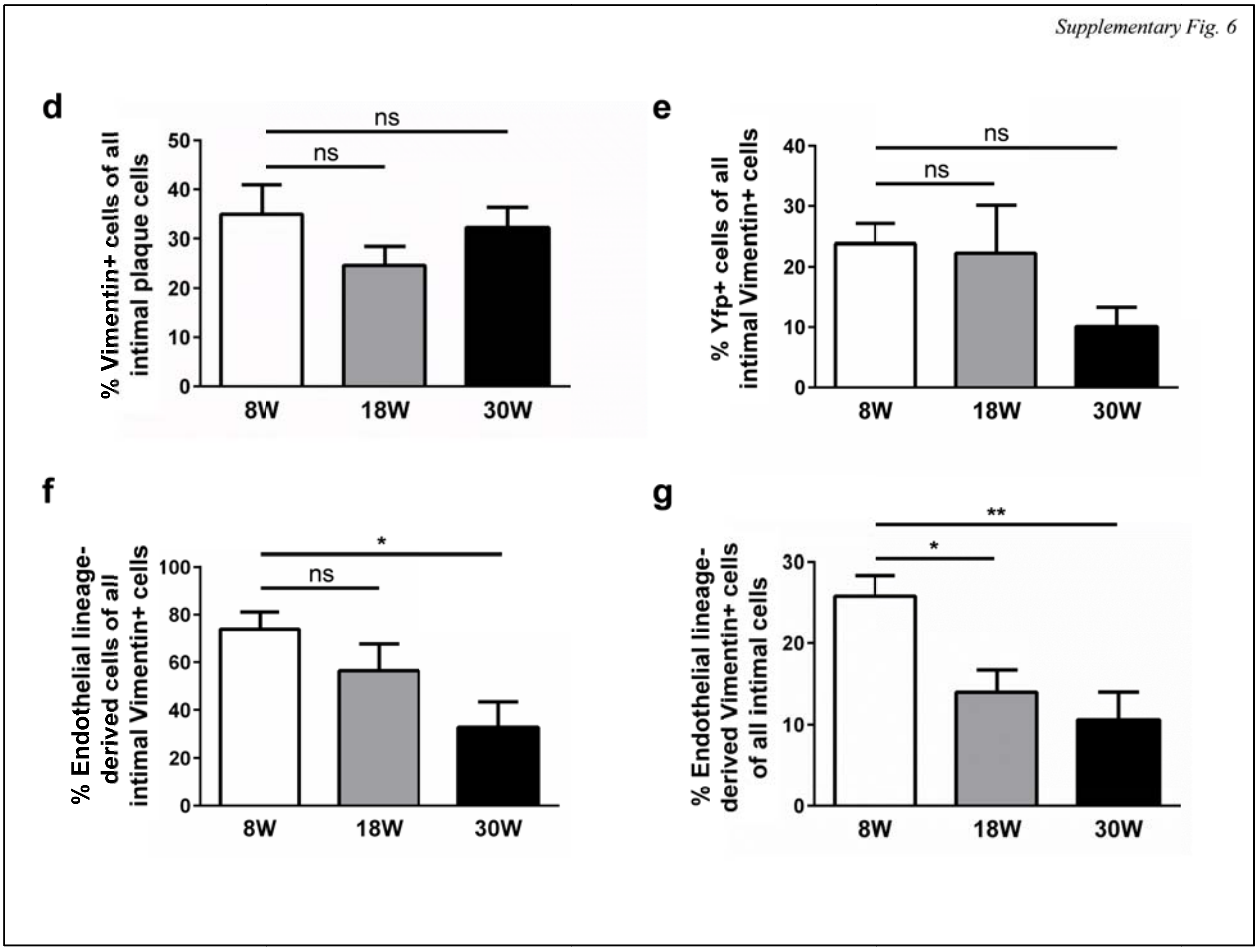

Supplementary Fig. 6 (continued). (d) Quantitation of cells in intimal plaques revealed that after 8, 18 and 30 weeks of HFD 34.9 ± 6.0%, 24.7 ± 3.9% and 32.2 ± 4.1% (respectively) of cells expressed Vimentin, indicative of a fibroblast phenotype. **(e)** Quantitation (not accounting for the proportion of endothelial cells expressing Yfp) identified that after the abovementioned periods of HFD 23.8 ± 3.3%, 22.3 ± 7.9% and 10.1 ± 3.2% (respectively) of intimal Vimentin^{+} cells co-expressed Yfp. **(f)** After taking into account the efficiency of our endothelial lineage tracking system (the % of endothelial cells expressing Yfp), at the same time-points, we determined that 73.8 ± 7.3%, 56.5 ± 11.2% and 32.9 ± 10.4% of intimal Vimentin^{+} cells were derived from endothelial lineage cells. **(g)** Again taking into account the efficiency of our endothelial lineage tracking system, at the same time-points respectively, we determined that 25.8 ± 2.5%, 13.9 ± 2.8% and 10.6 ± 3.4% of all intimal cells were endothelial lineage-derived Vimentin^{+} cells. Staining was performed at each time-point with at least 4 images evaluated from each of at least 3 spatially separated thoracic aortic sections per mouse (n = 5 mice for all time-points). Data were averaged per animal then used for statistical analyses. Analysis by 1-way ANOVA. $^{**}P < 0.01$.

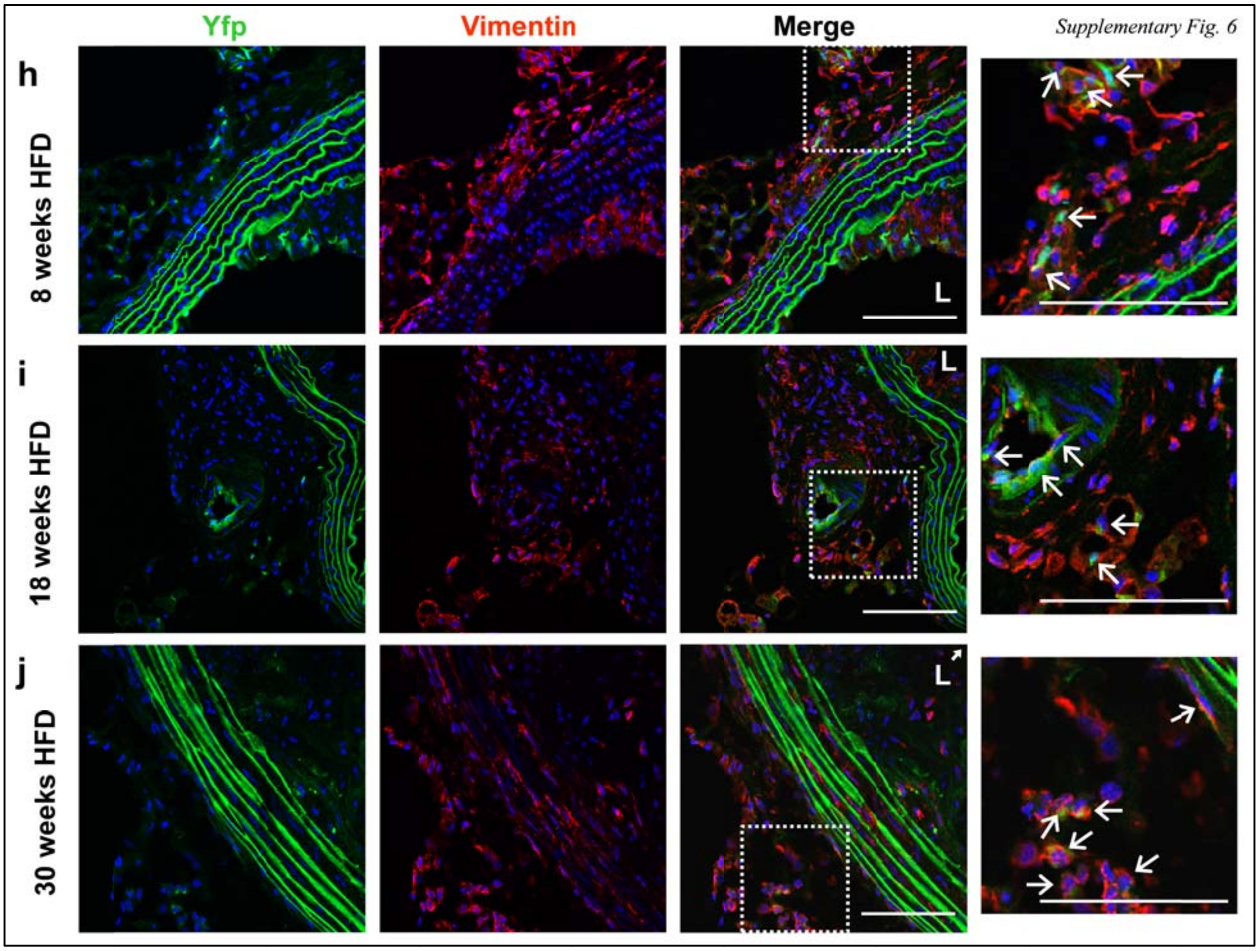

Supplementary Fig. 6 (continued). Immunofluorescence confocal microscopy of the adventitia **(h – j)** from thoracic aortic sections of tamoxifen-induced end.*SclCreER*^{T};*R26RstopYfp*;*ApoE*$^{-/-}$ mice fed with **(h)** 8, **(i)** 18 or **(j)** 30 weeks of HFD also revealed Yfp^{+} cells co-expressing the fibroblast marker Vimentin. L = lumen; scale bars represent 100 μm. Insets are shown at higher magnification as indicated and arrows indicate co-positive cells.

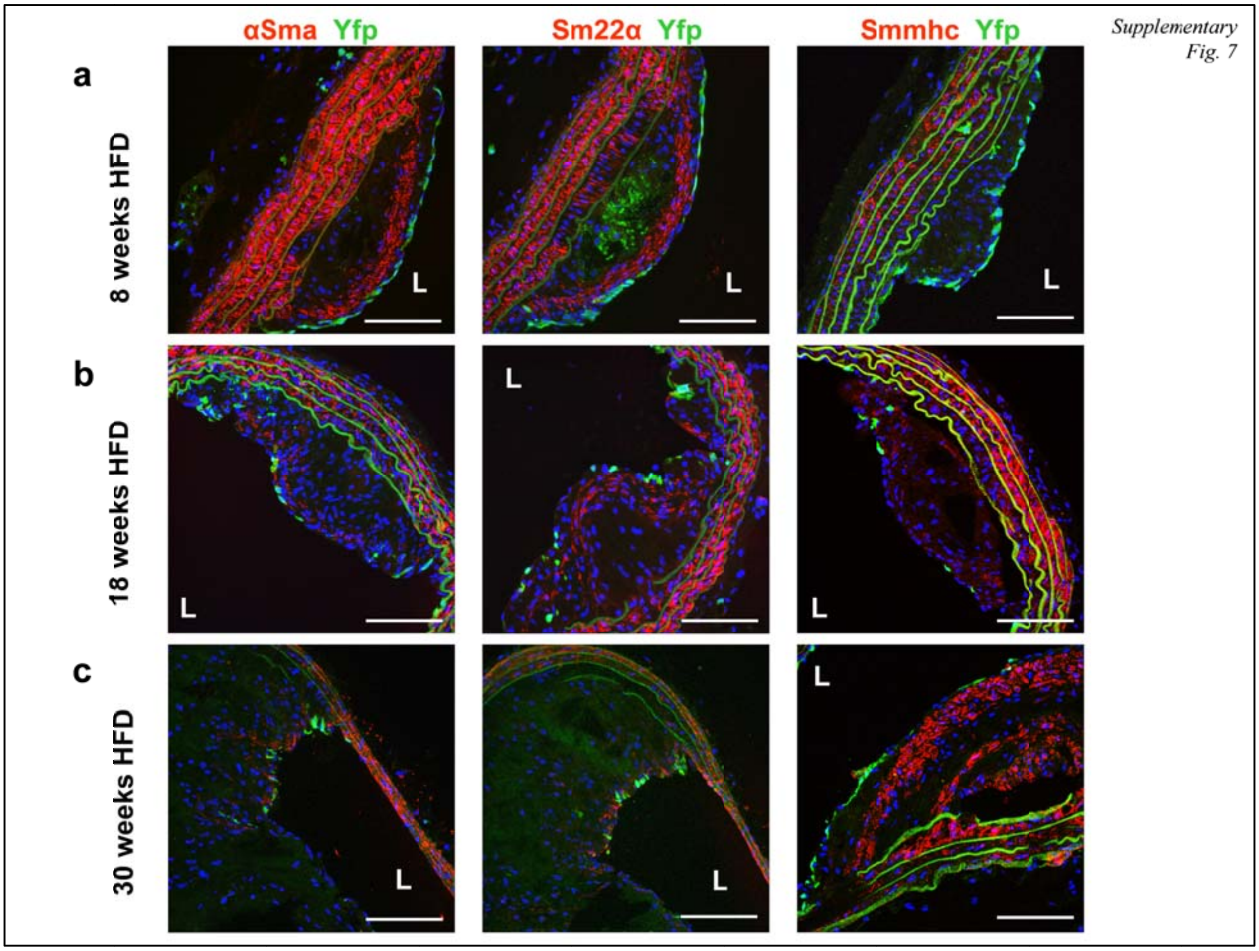

Supplementary Fig. 7. Endothelial lineage-derived cells give rise to a small proportion of αSma$^+$, Sm22α$^+$ or Smmhc$^+$ cells in murine atherosclerotic lesions. (a – c) Immunofluorescence confocal microscopy of thoracic aortic sections from tamoxifen-induced end.*SclCreER*^{T};*R26RstopYfp*;*ApoE*$^{-/-}$ mice fed with **(a)** 8, **(b)** 18 or **(c)** 30 weeks of HFD revealed that Yfp$^+$ cells give rise to a small proportion of αSma$^+$, Sm22α$^+$ or Smmhc$^+$ cells in this murine model. L = lumen; scale bars represent 100 µm.

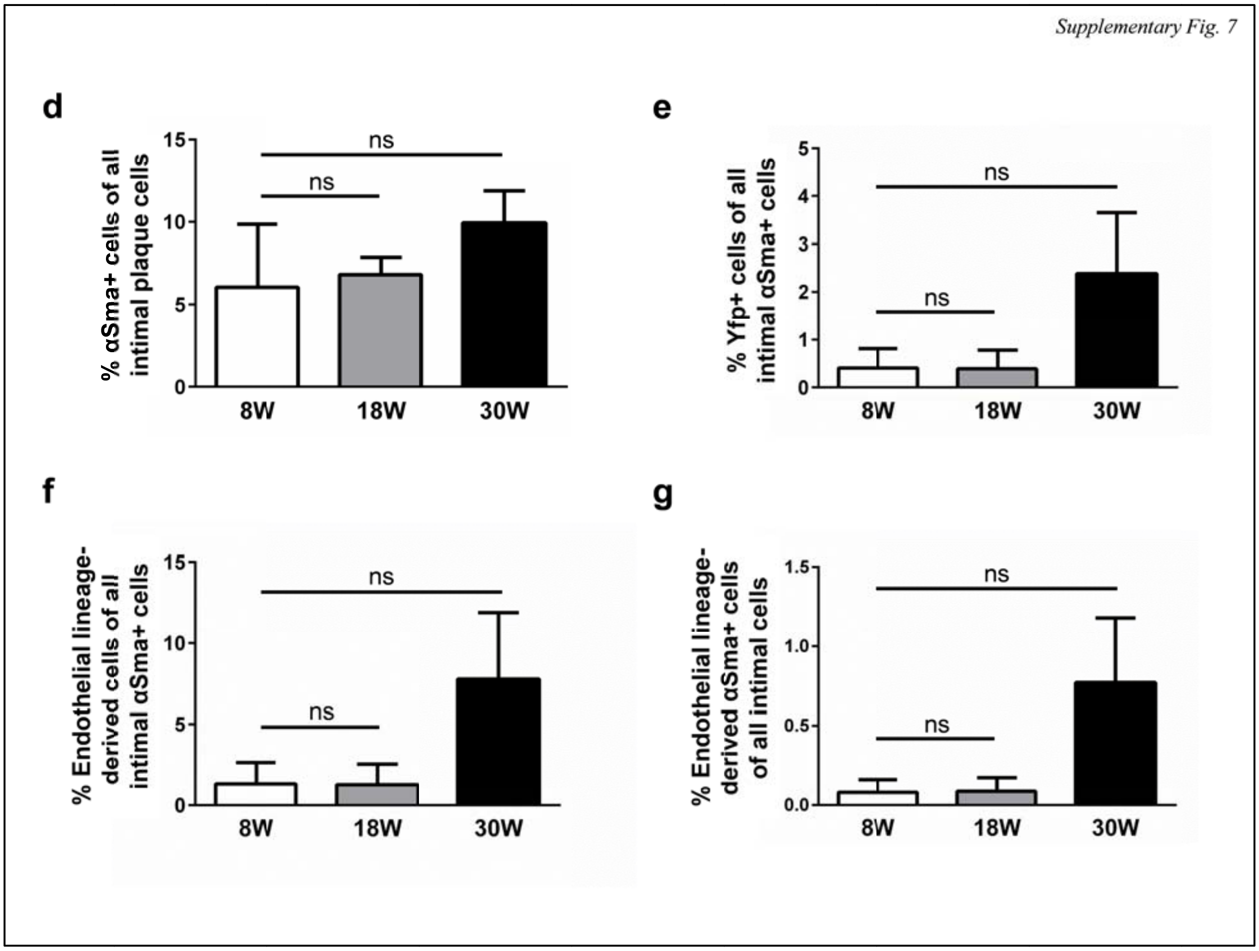

Supplementary Fig. 7 (continued). (d) Quantitation of cells in intimal plaques revealed that after 8, 18 and 30 weeks of HFD 6.1 ± 3.8%, 6.8 ± 0.8% and 9.9 ± 1.7% (respectively) of cells expressed αSma. **(e)** Quantitation (not accounting for the proportion of endothelial cells expressing Yfp) identified that after the abovementioned periods of HFD 0.4 ± 0.4%, 0.4 ± 0.3% and 2.4 ± 1.1% (respectively) of intimal αSma^{+} cells co-expressed Yfp. **(f)** After taking into account the efficiency of our endothelial lineage tracking system (the % of endothelial cells expressing Yfp), at the same time-points, we determined that 1.3 ± 1.4%, 1.3 ± 0.9% and 7.8 ± 3.7% of intimal αSma^{+} cells were derived from endothelial lineage cells. **(g)** Again taking into account the efficiency of our endothelial lineage tracking system, at the same time-points respectively, we determined that 0.08 ± 0.09%, 0.09 ± 0.06% and 0.8 ± 0.4% of all intimal cells were endothelial lineage-derived αSma^{+} cells. Staining was performed at each time-point with at least 4 images evaluated from each of at least 3 spatially separated thoracic aortic sections per mouse (*n* = 5 mice for all time-points). Data were averaged per animal then used for statistical analyses. Analysis by 1-way ANOVA.

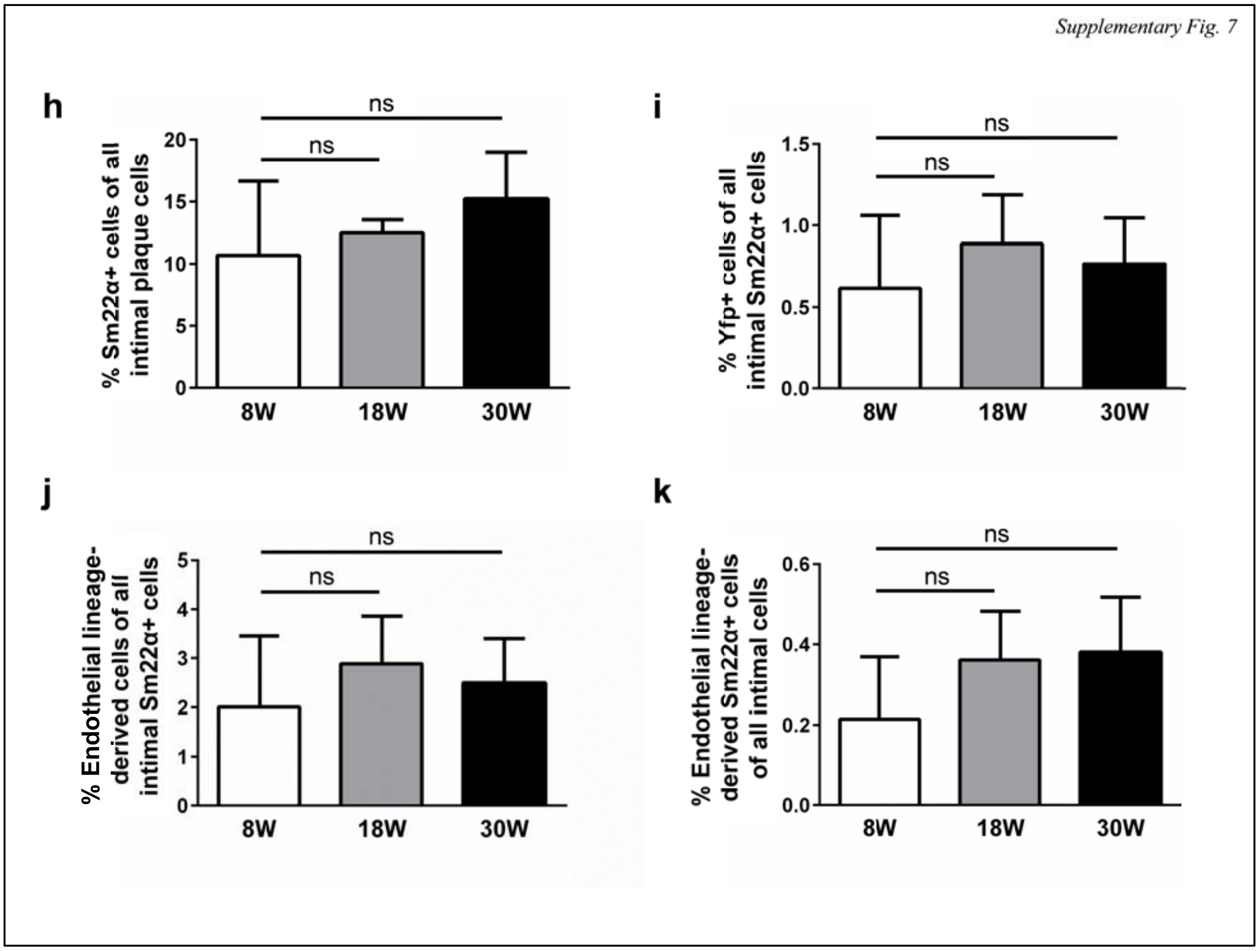

Supplementary Fig. 7 (continued). (h) Quantitation of cells in intimal plaques revealed that after 8, 18 and 30 weeks of HFD 10.7 ± 5.3%, 12.5 ± 0.8% and 15.2 ± 3.4% (respectively) of cells expressed Sm22α. **(i)** Quantitation (not accounting for the proportion of endothelial cells expressing Yfp) identified that after the abovementioned periods of HFD 0.6 ± 0.4%, 0.9 ± 0.2% and 0.8 ± 0.2% (respectively) of intimal Sm22α$^+$ cells co-expressed Yfp. **(j)** After taking into account the efficiency of our endothelial lineage tracking system (the % of endothelial cells expressing Yfp), at the same time-points, we determined that 2.0 ± 1.3%, 2.9 ± 0.7% and 2.5 ± 0.8% of intimal Sm22α$^+$ cells were derived from endothelial lineage cells. **(k)** Again taking into account the efficiency of our endothelial lineage tracking system, at the same time-points respectively, we determined that 0.2 ± 0.1%, 0.4 ± 0.08% and 0.4 ± 0.1% of all intimal cells were endothelial lineage-derived Sm22α$^+$ cells. Staining was performed at each time-point with at least 4 images evaluated from each of at least 3 spatially separated thoracic aortic sections per mouse (*n* = 5 mice for all time-points). Data were averaged per animal then used for statistical analyses. Analysis by 1-way ANOVA.

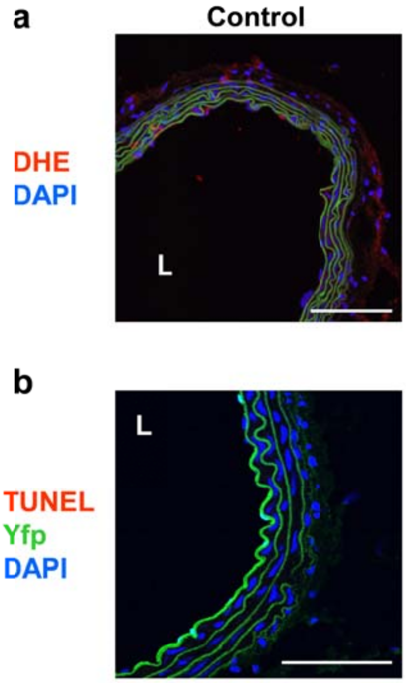

Supplementary Fig. 8. Lack of oxidative stress and cell apoptosis in non-atherosclerotic (control) vessels. Control end.*SclCreER*^{T};*R26RstopYfp*;*ApoE*$^{-/-}$ mice of the same age as those shown in Figs. 3a and 3b, fed a chow diet, were stained with the identical DHE **(a)** and TUNEL **(b)** staining protocols as per Figs. 3a and 3b. No appreciable signal was seen for either DHE or TUNEL staining in these samples. L = lumen; scale bars represent 100 µm.

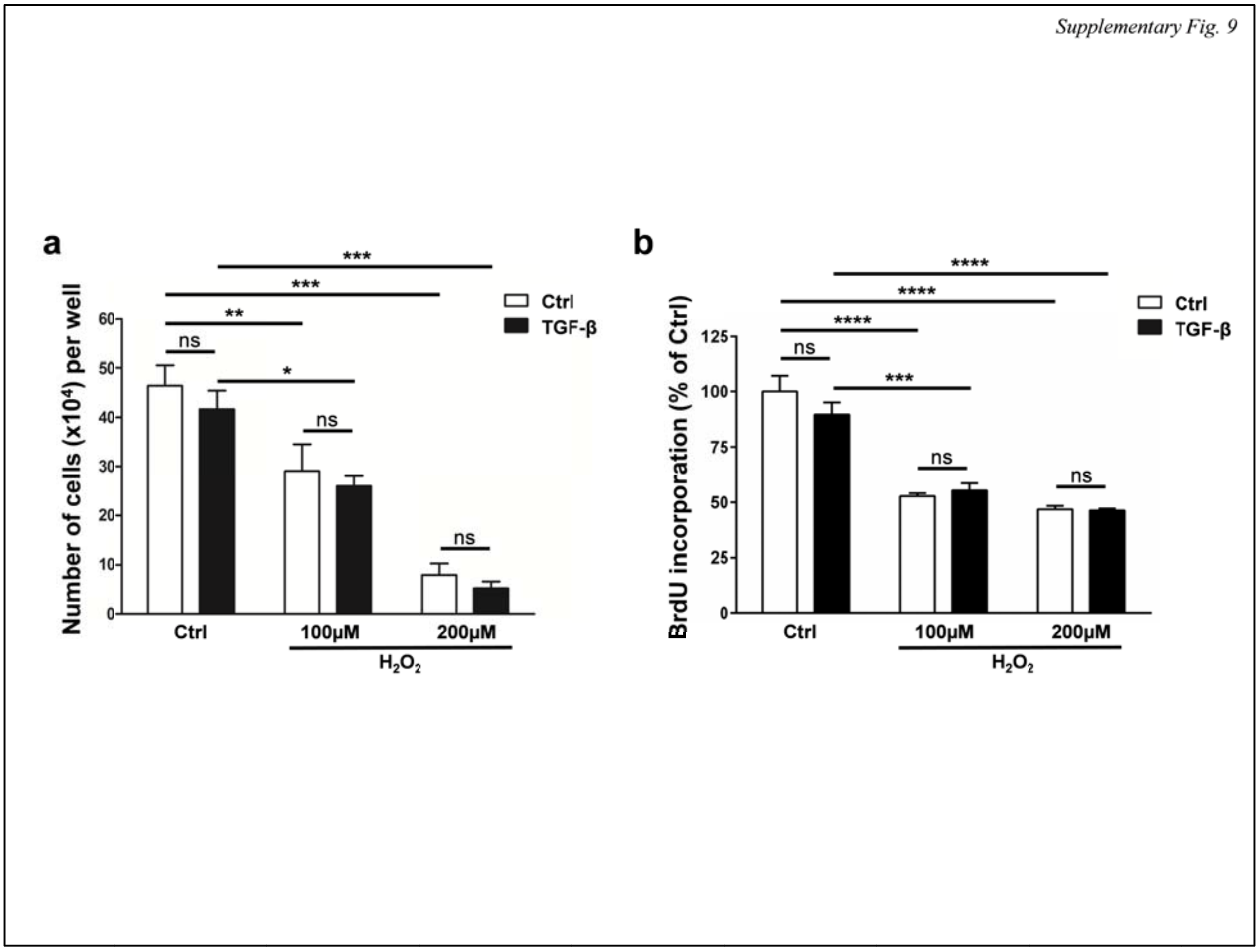

Supplementary Fig. 9. Cellular effects of TGF-β and H_2O_2 on HUVECs. **(a)** At the described doses used to induce EndMT, TGF-β did not affect the total number of cells in culture (per well of 6-well culture plate), while H_2O_2 caused a dose-responsive reduction in cell count during EndMT induction over 5 days. *n* = 3 per condition. **(b)** Cell proliferation assessed by BrdU incorporation during the initial 24 hours of EndMT induction was unchanged by TGF-β, while H_2O_2 caused a dose-responsive reduction in cell proliferation. *n* = 3 per condition. Data in Supplementary Fig. 9 were analyzed by 2-way ANOVA with complete results presented in Supplementary Table 1. ns not significant, $*P < 0.05$, $**P < 0.01$, $***P < 0.001$, $****P < 0.0001$.

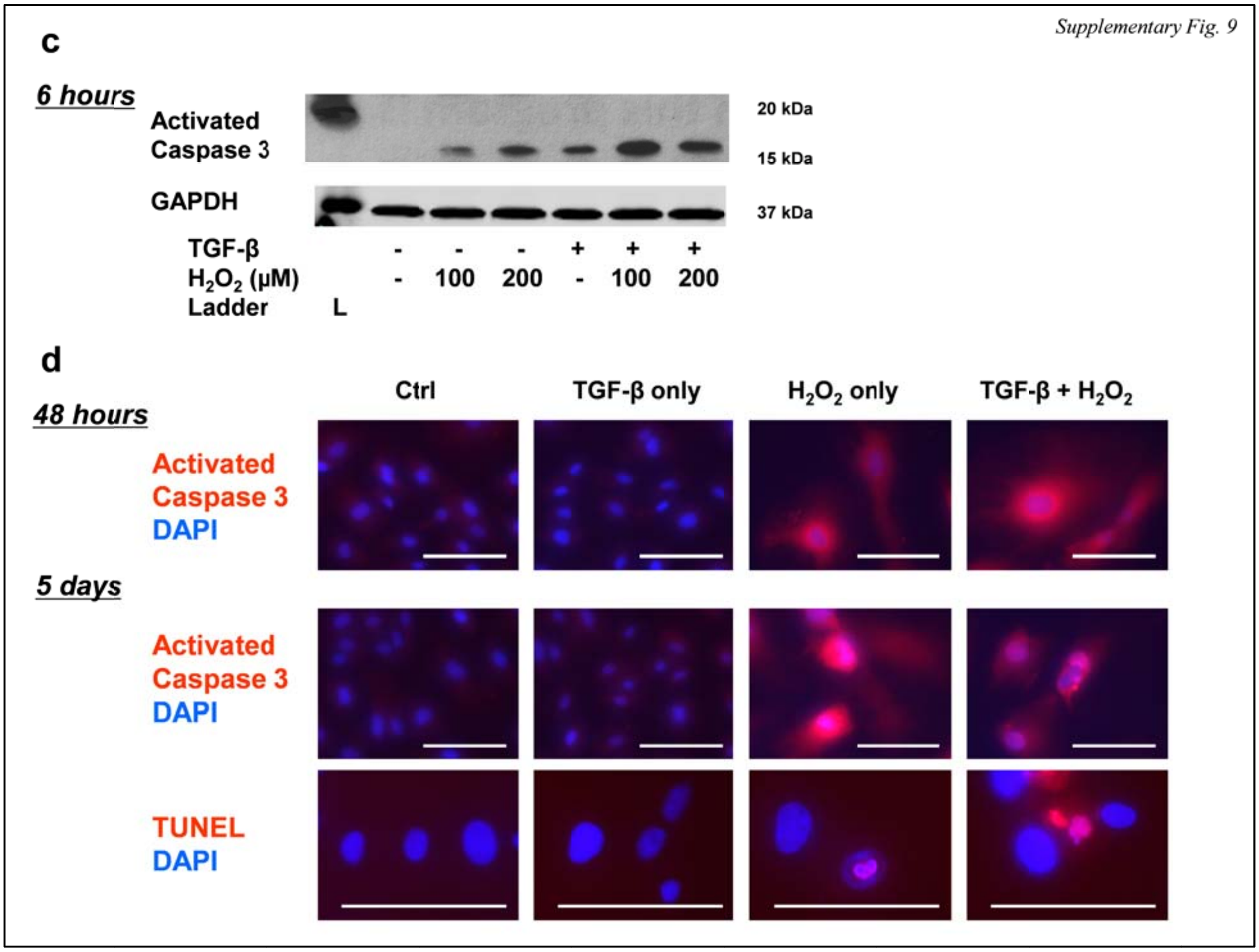

Supplementary Fig. 9 (continued). (c) Endothelial cell apoptosis in response to 6 hours EndMT induction assessed by Western blot for activated Caspase 3 protein expression in HUVECs with H_2O_2 applied at the indicated concentrations. Activated Caspase 3 ladder (L) represents 20kDa. Activated Caspase 3 expected molecular weight/size is 17kDa. GAPDH ladder represents 37kDa. GAPDH expected molecular weight/size is 37kDa. **(d)** Endothelial cell apoptosis in response to EndMT induction in HUVECs assessed by immunostaining for active Caspase 3 (after 48 hours and 5 days EndMT induction) and TUNEL (5 days EndMT induction) following treatment with endothelial growth media alone (Ctrl), TGF-β only, 200μM H_2O_2 only, or TGF-β plus 200μM H_2O_2. Scale bars represent 100 μm.

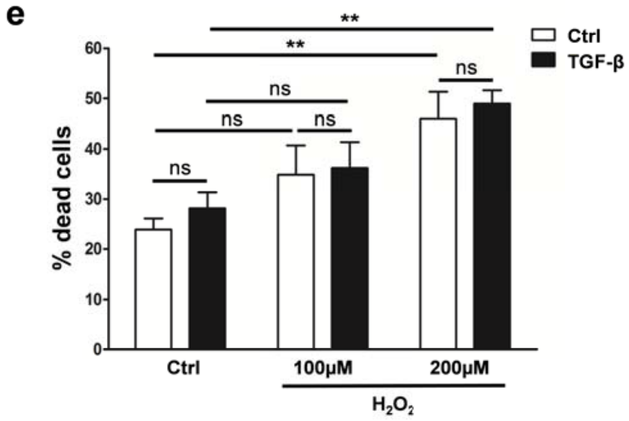

Supplementary Fig. 9 (continued). (e) H_2O_2, but not TGF-β, increased cell death after 48 hours of EndMT induction. *n* = 4 per condition. Data in Supplementary Fig. 9 were analyzed by 2-way ANOVA with complete results presented in Supplementary Table 1. ns not significant, *P < 0.05, **P < 0.01, ***P < 0.001, ****P < 0.0001. *Note that while in other EndMT experiments endothelial cells were treated with TGF-β and/or H_2O_2 for 5 days, to evaluate the effects of these agents on the EndMT process (rather than resulting mesenchymal cells), experiments b, c, e, and some of d in Supplementary Fig. 9 were performed at earlier time-points as indicated.*

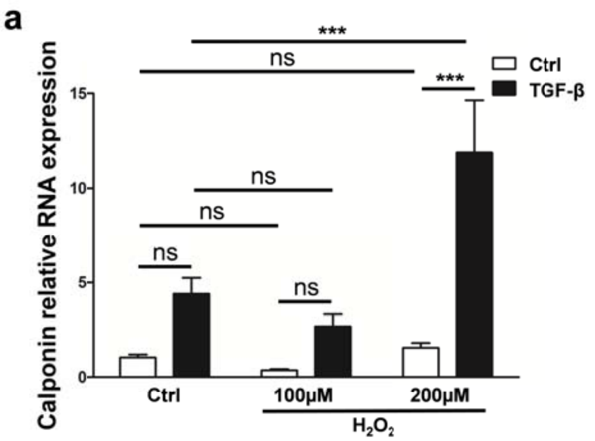

Supplementary Fig. 10. Oxidative stress augments EndMT and induces TGF-β pathway activation in HUVECs. (a, b) Relative RNA expression of Calponin and Versican (respectively) assessed by qRT-PCR was increased by both TGF-β and H_2O_2 in an additive fashion. All experiments in Supplementary Fig. 10 involved induction of EndMT by cell stimulation with TGF-β and/or H_2O_2 over 5 days (see methods). Data in Supplementary Fig. 10 were analyzed by 2-way ANOVA with complete results presented in Supplementary Table 4. ns not significant, *$P < 0.05$, **$P < 0.01$, ***$P < 0.001$, ****$P < 0.0001$. n = 6 - 9 for qRT-PCR.

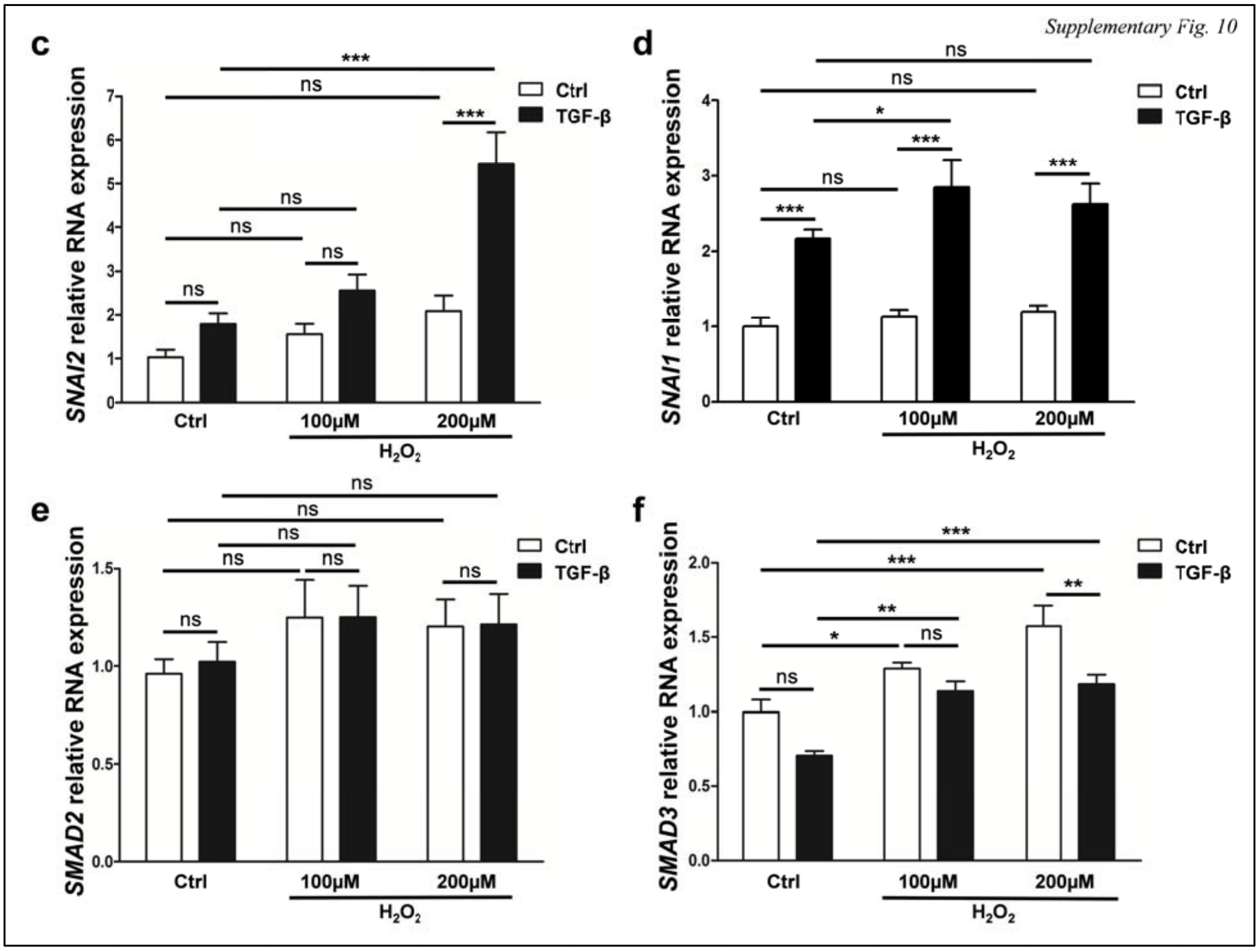

Supplementary Fig. 10 (continued). (c) Relative *SNAI2* RNA expression assessed by qRT-PCR was increased by both TGF-β and H_2O_2 in an additive fashion. **(d)** *SNAI1* RNA expression was increased by TGF-β, while H_2O_2 had a marginal effect. **(e)** *SMAD2* expression was not affected by TGF-β or H_2O_2. **(f)** *SMAD3* RNA expression was increased by H_2O_2, but was decreased by TGF-β. All experiments in Supplementary Fig. 10 involved induction of EndMT by cell stimulation with TGF-β and/or H_2O_2 over 5 days (see methods). Data in Supplementary Fig. 10 were analyzed by 2-way ANOVA with complete results presented in Supplementary Table 4. ns not significant, $*P < 0.05$, $**P < 0.01$, $***P < 0.001$, $****P < 0.0001$. $n = 6 - 9$ for qRT-PCR.

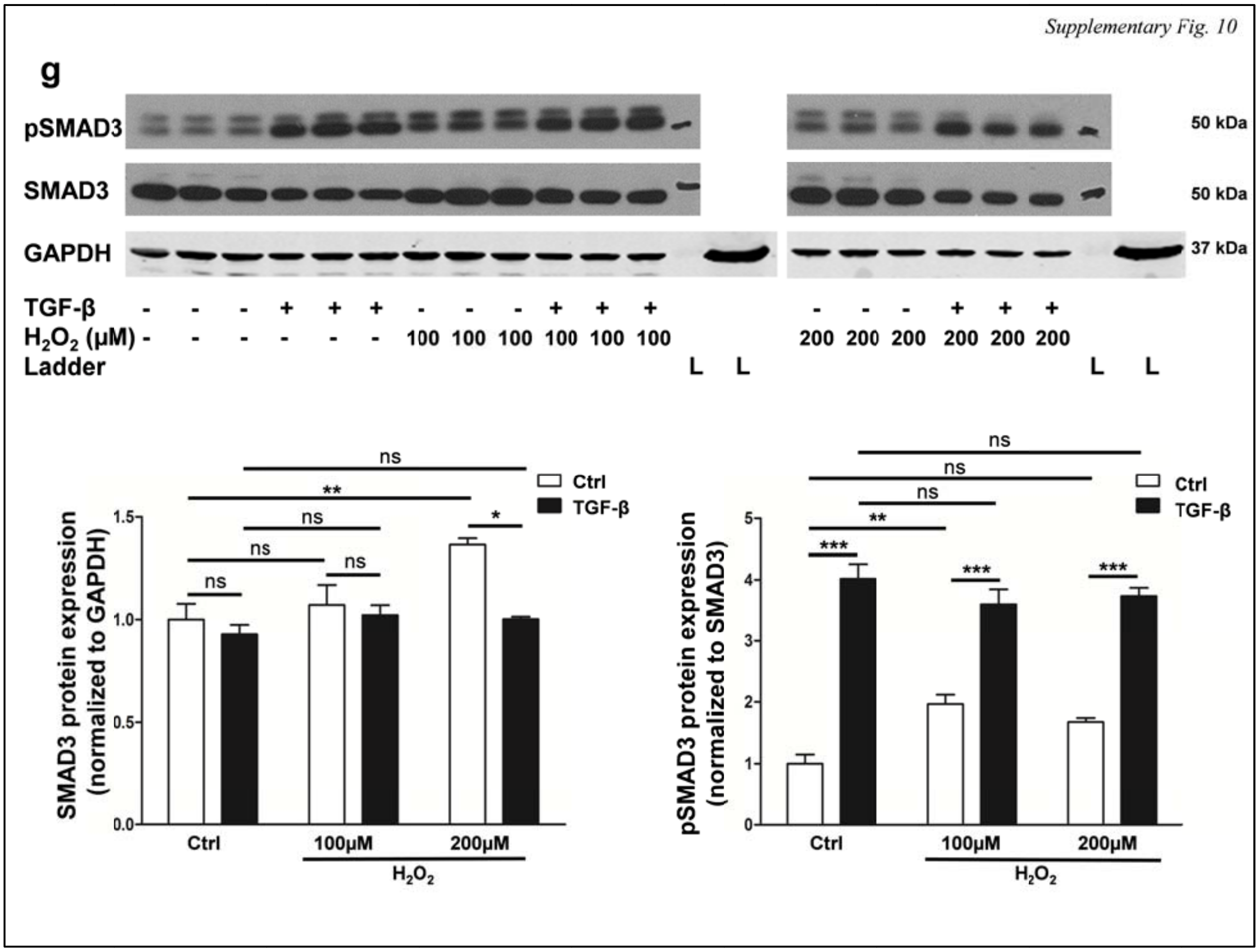

Supplementary Fig. 10 (continued). (g) Western blots (above) showing protein expression levels of pSMAD3, SMAD3 and GAPDH on exposure of HUVECs to TGF-β and/or H_2O_2. Quantitation (below left) of Western blots for SMAD3 protein levels, showing that H_2O_2 but not TGF-β increases SMAD3 in HUVECs. Quantitation (below right) of Western blots showing pSMAD3 protein levels (relative to SMAD3), with both TGF-β and H_2O_2 causing activation of SMAD3. pSMAD3 ladder (L) represents 50kDa. pSMAD3 expected molecular weight/size is 48kDa. SMAD3 ladder (L) represents 50kDa. SMAD3 expected molecular weight/size is 52kDa. GAPDH ladder represents 37kDa. GAPDH expected molecular weight/size is 37kDa. All experiments in Supplementary Fig. 10 involved induction of EndMT by cell stimulation with TGF-β and/or H_2O_2 over 5 days (see methods). Data in Supplementary Fig. 10 were analyzed by 2-way ANOVA with complete results presented in Supplementary Table 4. ns not significant, $*P < 0.05$, $**P < 0.01$, $***P < 0.001$, $****P < 0.0001$. $n = 3 - 6$ for Western blots.

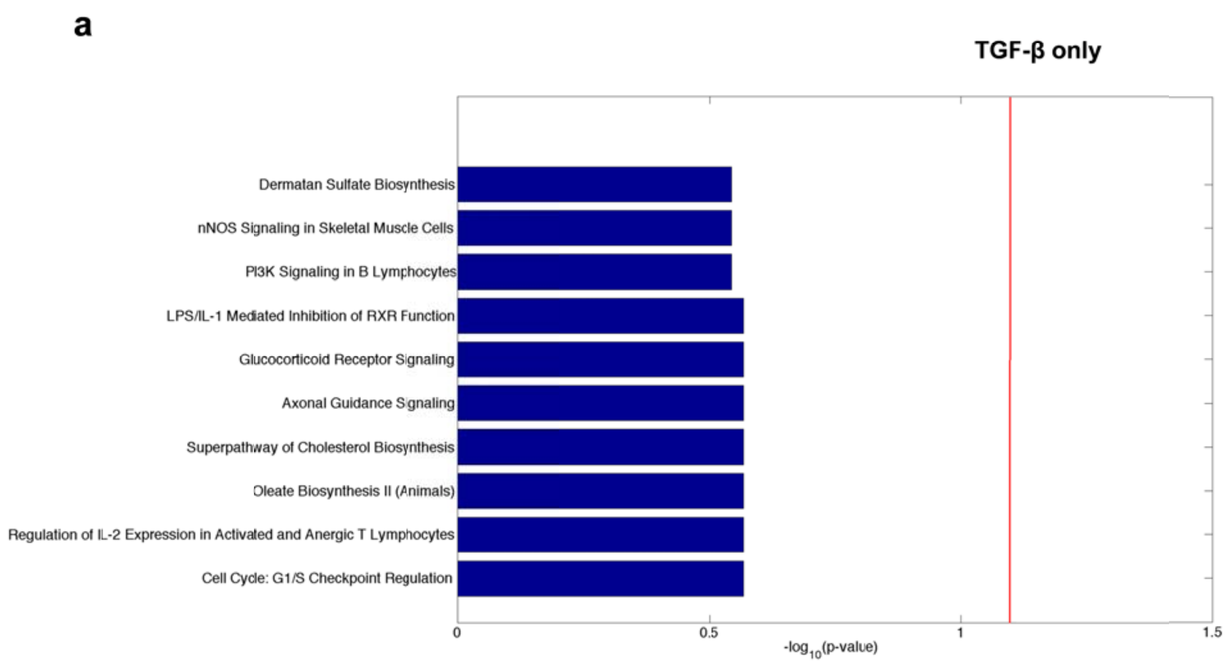

Supplementary Fig. 11. Oxidative stress causes inflammatory and fibrotic pathway upregulation in HUVECs. (a) Pathway analysis performed on upregulated genes identified from microarray data indicated that at the doses used for EndMT induction TGF-β alone did not upregulate any specific pathways in HUVECs. Significant upregulation is indicated if the horizontal (blue) pathway bar crosses to the right of the vertical red line.

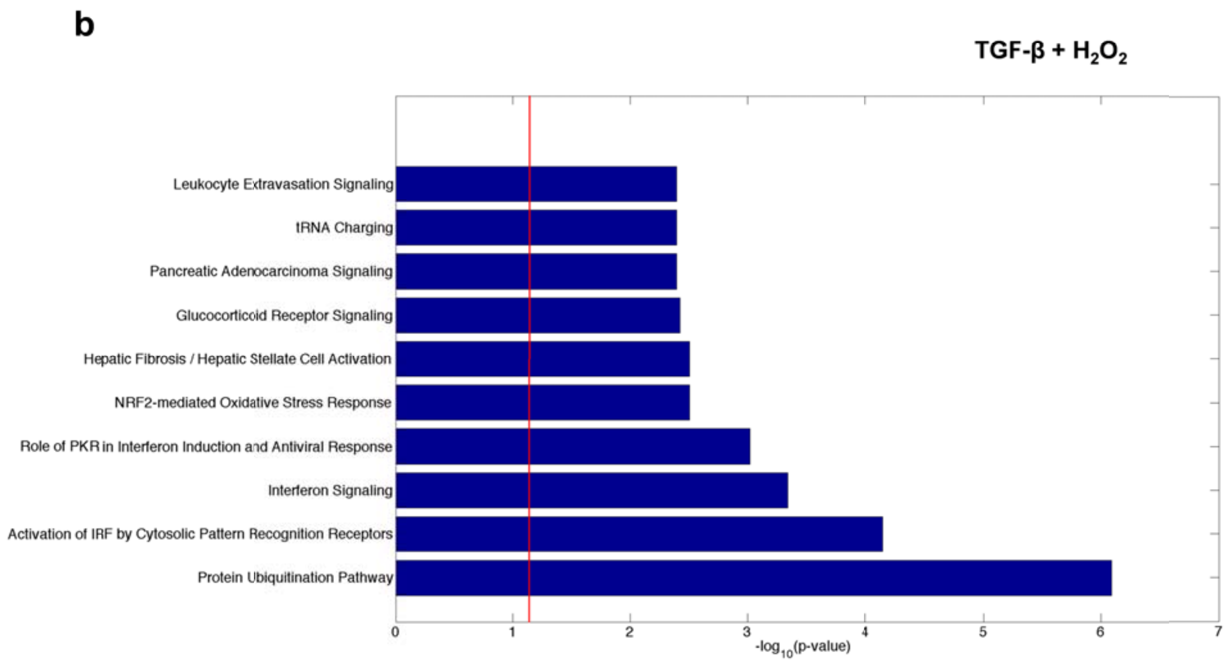

Supplementary Fig. 11 (continued). (b) In contrast, TGF-β+H_2O_2 caused a significant upregulation of a number of pathways, with many of these being related to oxidative stress, inflammation and fibrosis. The 10 most significantly upregulated pathways are presented. Significant upregulation is indicated if the horizontal (blue) pathway bar crosses to the right of the vertical red line.

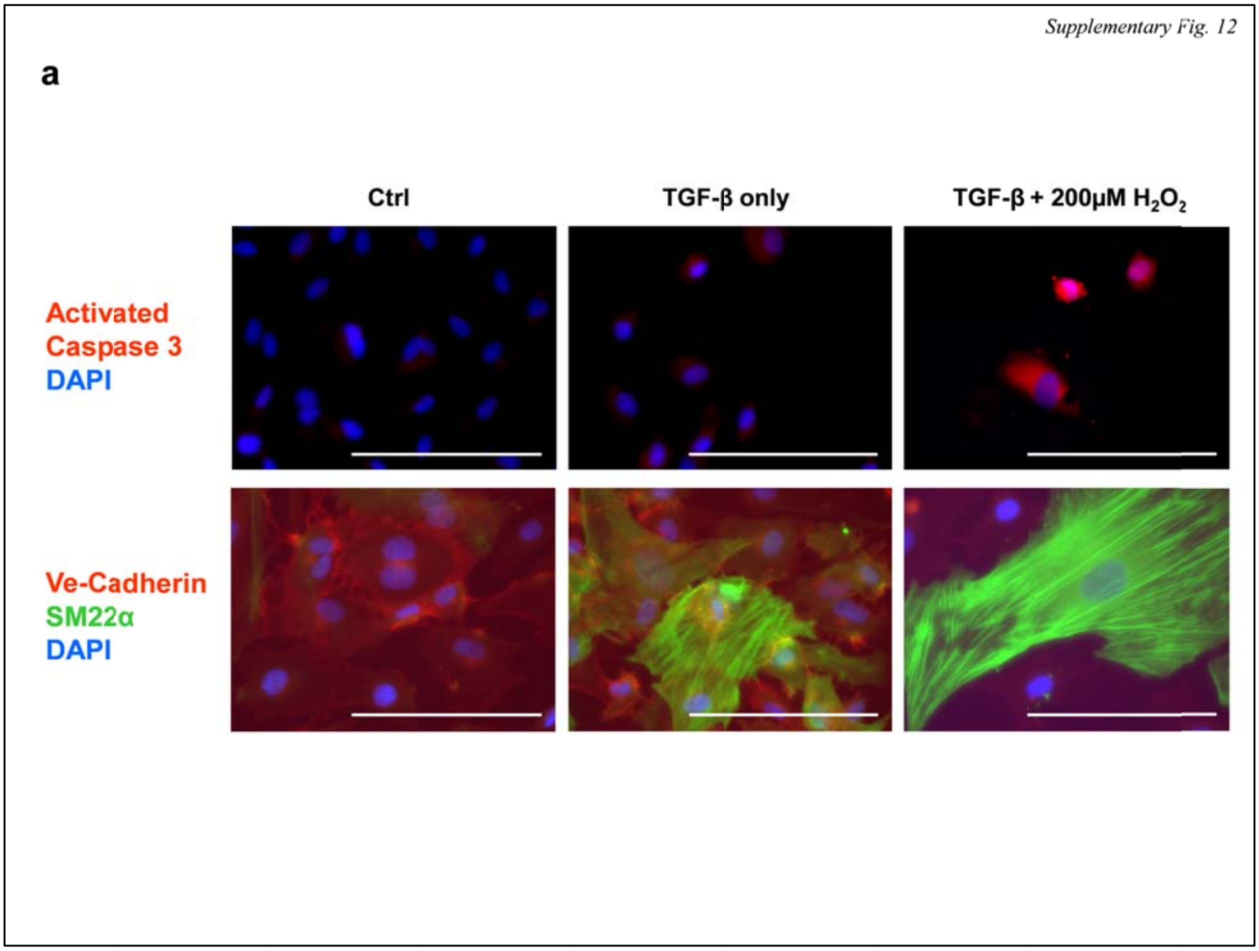

Supplementary Fig. 12. Oxidative stress increases EndMT and is additive to the effect of TGF-β in HCAECs. (a) Immunostaining of HCAECs treated with endothelial growth media alone (Ctrl), TGF-β, or TGF-β plus 200μM H_2O_2, indicating induction of cellular stress with positive staining for active Caspase 3, and a progressive increase in EndMT with loss of Ve-Cadherin staining and gain of mesenchymal protein expression (in this case, SM22α) with TGF-β, and particularly TGF-β plus 200μM H_2O_2. Scale bars represent 100 μm.

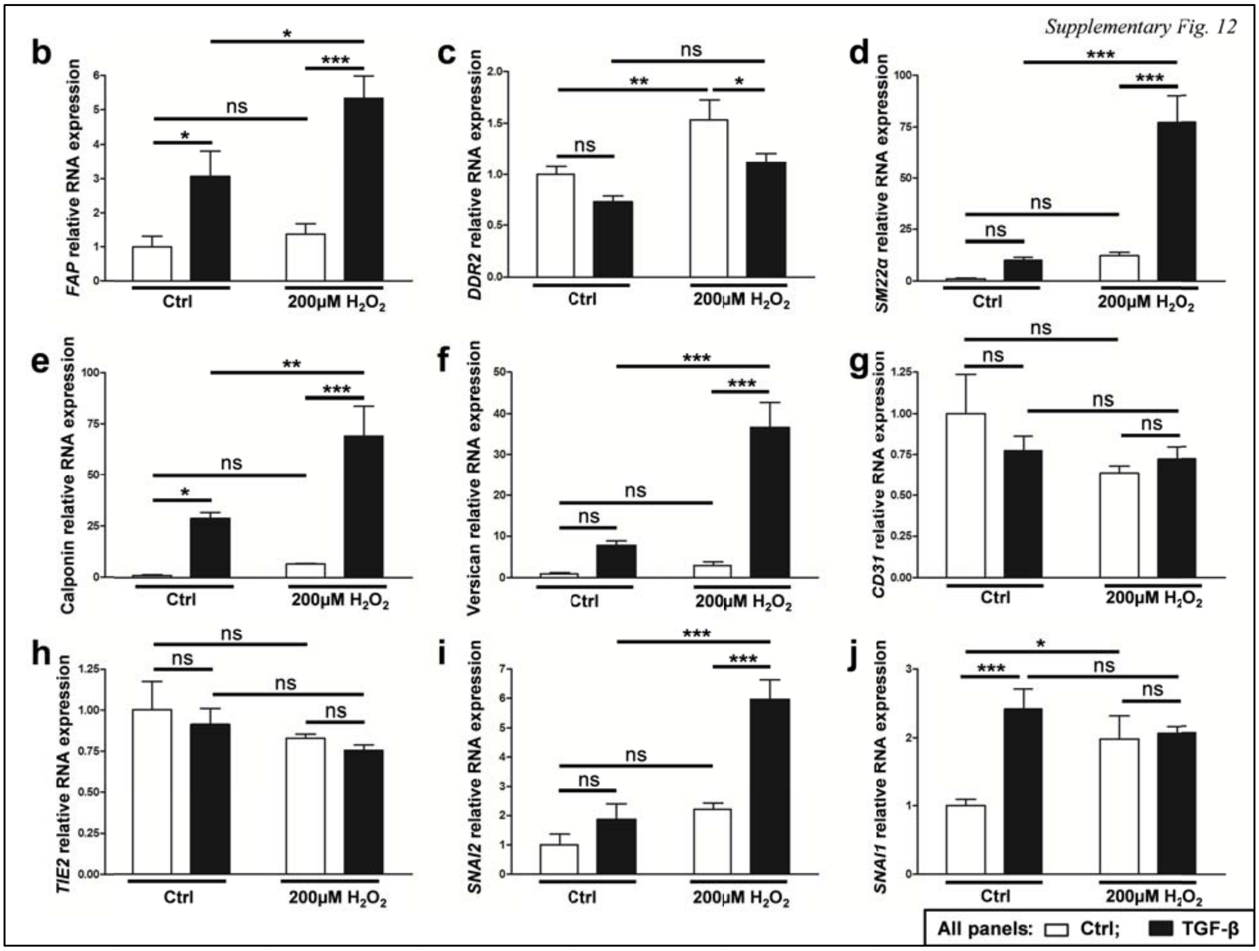

Supplementary Fig. 12 (continued). (b - f) Relative RNA expression of *FAP, DDR2, SM22α,* Calponin and Versican (respectively) in HCAECs assessed by qRT-PCR in response to treatment with TGF-β and H_2O_2. **(g, h)** Relative RNA expression of *CD31* and *TIE2* (respectively) assessed by qRT-PCR was non-significantly decreased by TGF-β and H_2O_2. **(i)** Relative RNA expression of *SNAI2* assessed by qRT-PCR was increased by both TGF-β and H_2O_2 in an additive fashion. **(j)** Relative RNA expression of *SNAI1* assessed by qRT-PCR was increased by both TGF-β and H_2O_2, but unlike *SNAI2* this effect was not additive. All experiments in Supplementary Fig. 12 involved induction of EndMT by cell stimulation with TGF-β and/or H_2O_2 over 5 days (see methods). Data in Supplementary Fig. 12 were analyzed by 2-way ANOVA with complete results presented in Supplementary Table 5. ns not significant, $^{*}P < 0.05$, $^{**}P < 0.01$, $^{***}P < 0.001$, $^{****}P < 0.0001$. $n = 6$ in all experiments.

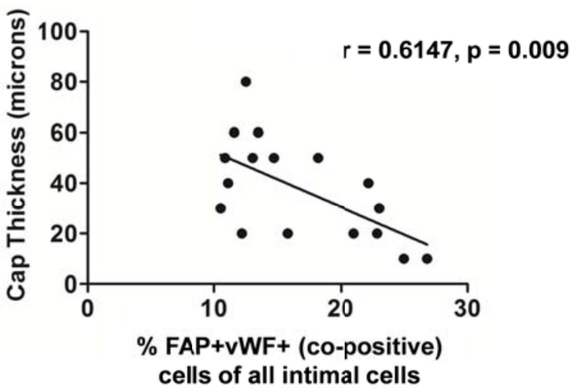

Supplementary Fig. 13. EndMT is more common in plaques with thin fibrous cap. Plaques identified in human samples obtained at autopsy from the abdominal aorta were classified as AHA type V or VI according to standard criteria (see methods). The % of co-positive cells per 20x field was expressed as a function of the total number of $DAPI^+$ cells (per field). Limited scatter plot and regression line for aortic plaque cap thickness versus % FAP^+vWF^+ co-positive cells. Only plaques with cap thickness < 100 µm are presented in this analysis. r = 0.6147, p = 0.009. After exclusion of 7 plaques with cap thickness > 100 µm from our original data, a total of 17 separate plaques from 10 patients are presented.

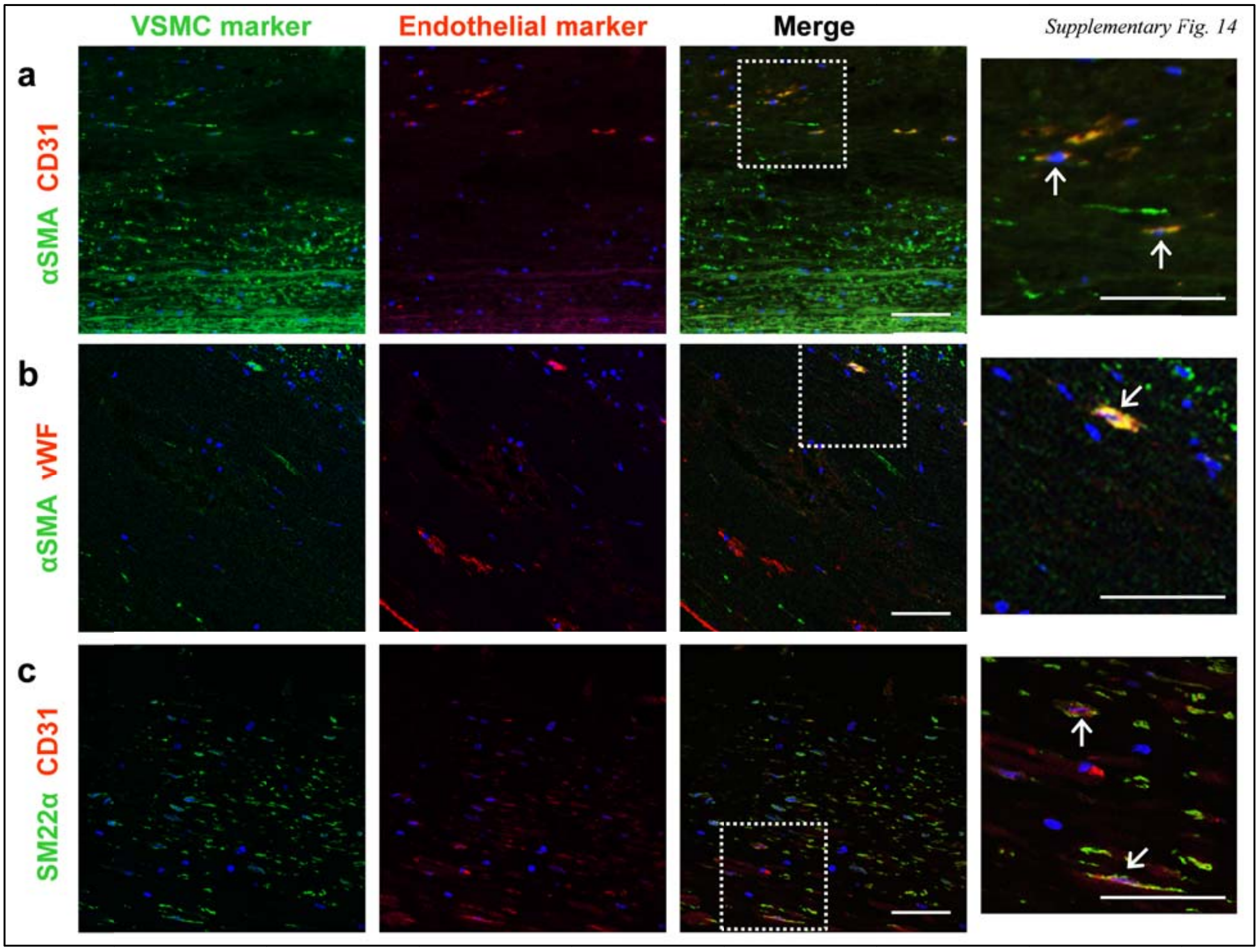

Supplementary Fig. 14. EndMT gives rise to infrequent vascular smooth muscle cells in human atherosclerosis. AHA type V plaques were identified from human aortic samples obtained at autopsy. Plaques were classified as AHA type V according to standard criteria (see methods). Staining was performed using various endothelial-VSMC marker combinations as follows: **(a)** αSMA/CD31, **(b)** αSMA/vWF, **(c)** SM22α/CD31. All scale bars represent 50 µm. Insets are shown at higher magnification as indicated and arrows indicate co-positive cells.

Supplementary Fig. 14

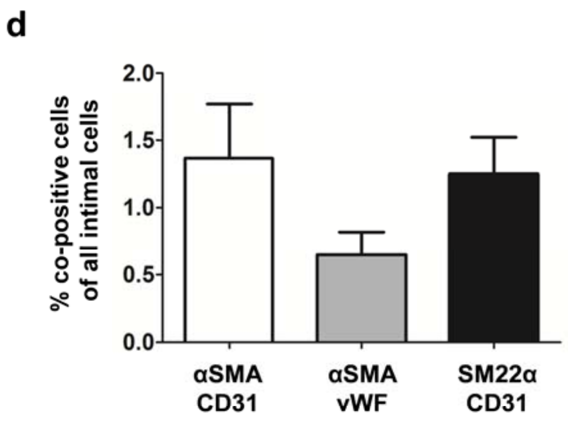

Supplementary Fig. 14 (continued). (d) The % of co-positive cells per 20x field was expressed as a function of the total number of DAPI^{+} cells (per field). Two separate plaques from 5 (αSMA/CD31 and SM22α/CD31) or 6 (αSMA/vWF) different patients were randomly included per analysis.

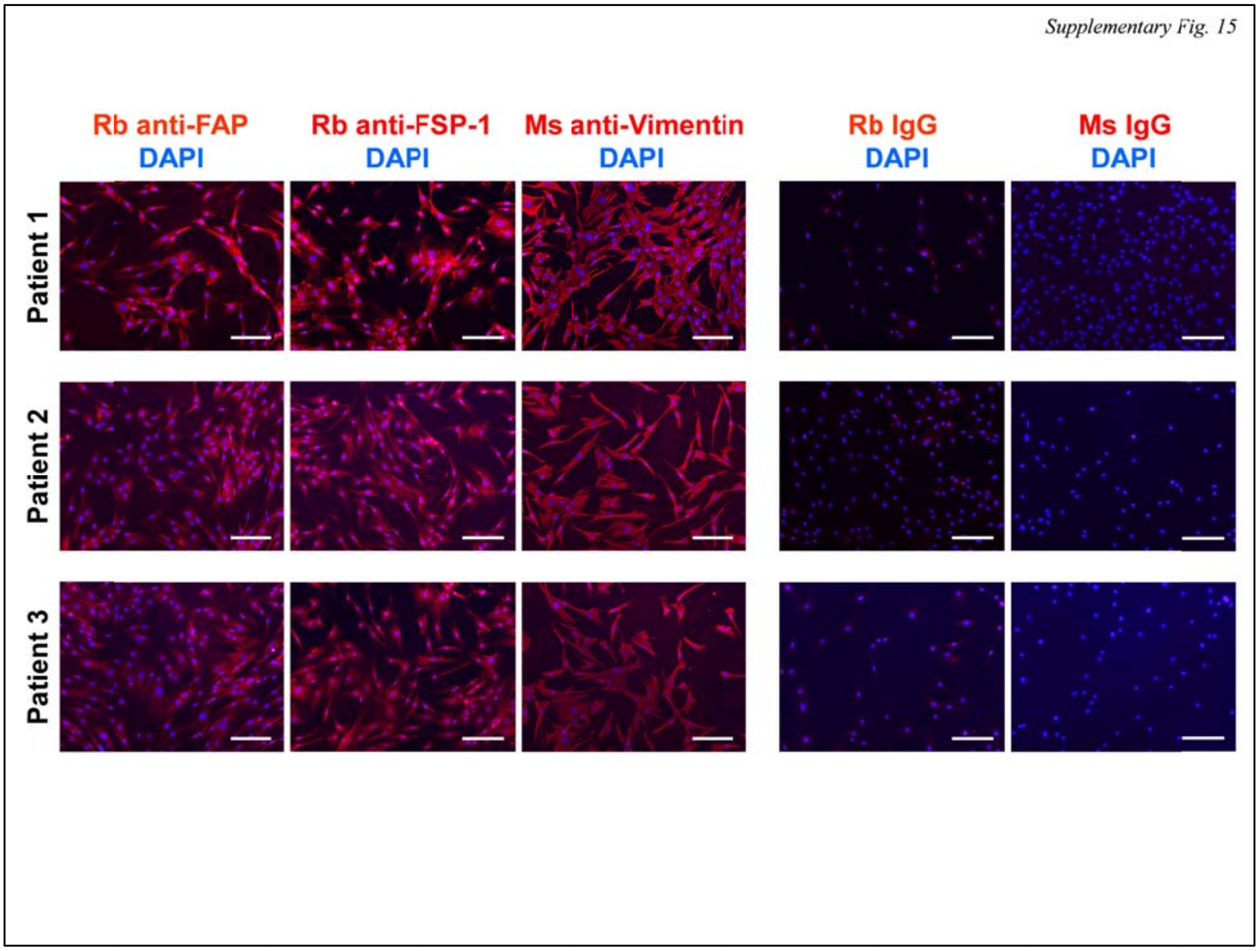

Supplementary Fig. 15. Characterization of human fibroblast cell lines. Immunostaining of fibroblast cell lines from 3 healthy control subjects for FAP, FSP-1 and Vimentin. These fibroblast lines are those used in the microarray and MMP expression experiments shown in Figs. 6a - f. Corresponding IgG control staining is shown. Rb rabbit; Ms mouse. Scale bars represent 100 μm.

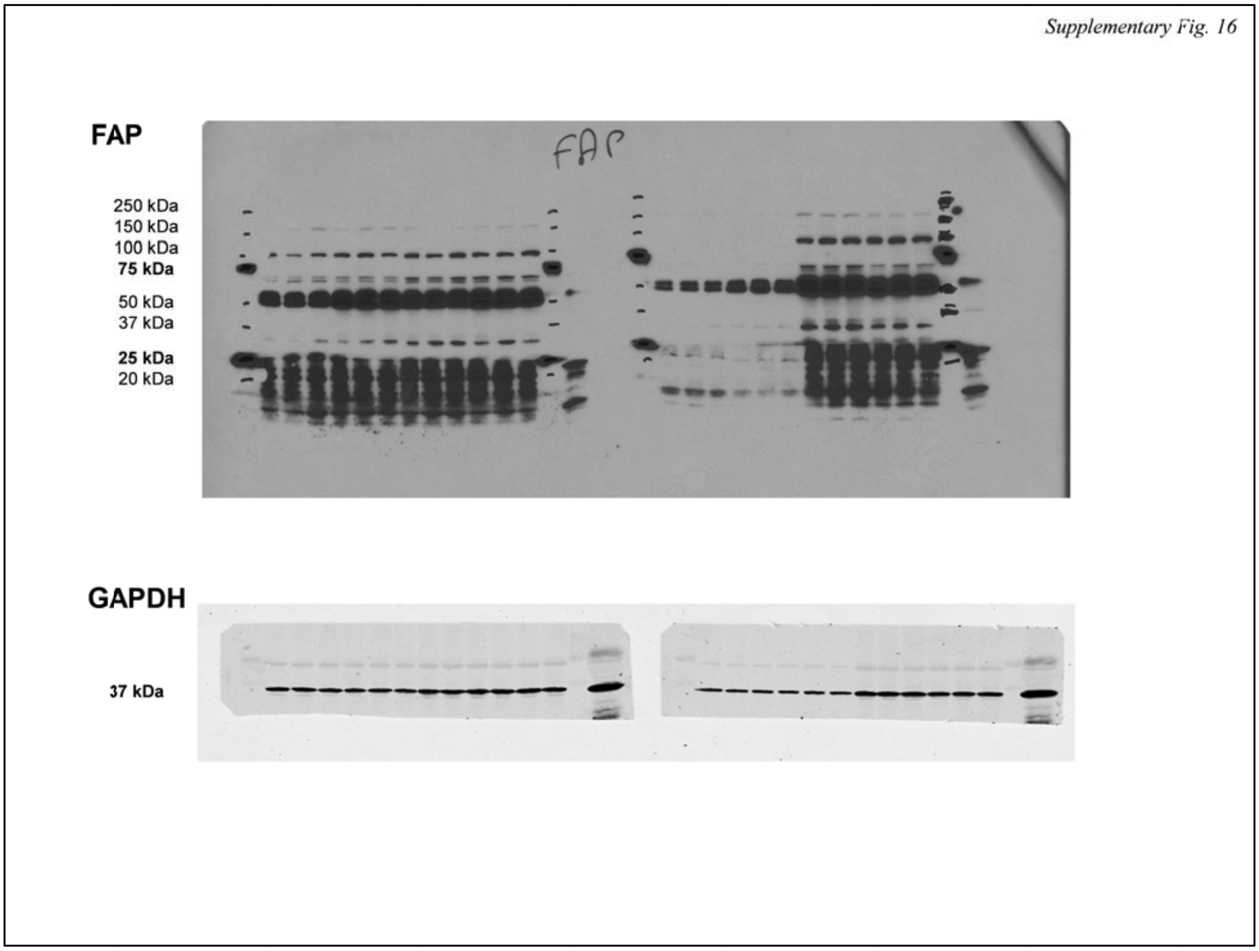

Supplementary Fig. 16. Original immunoblots from Fig. 3e. Ladder marker sizes shown on left. FAP expected molecular weight/size is 90kDa. GAPDH expected molecular weight/size is 37kDa. 2 membranes were imaged adjacent to one another. On the right membranes, the first 6 lanes were control lanes loaded with substantially lower levels of protein and not included in the main Figures.

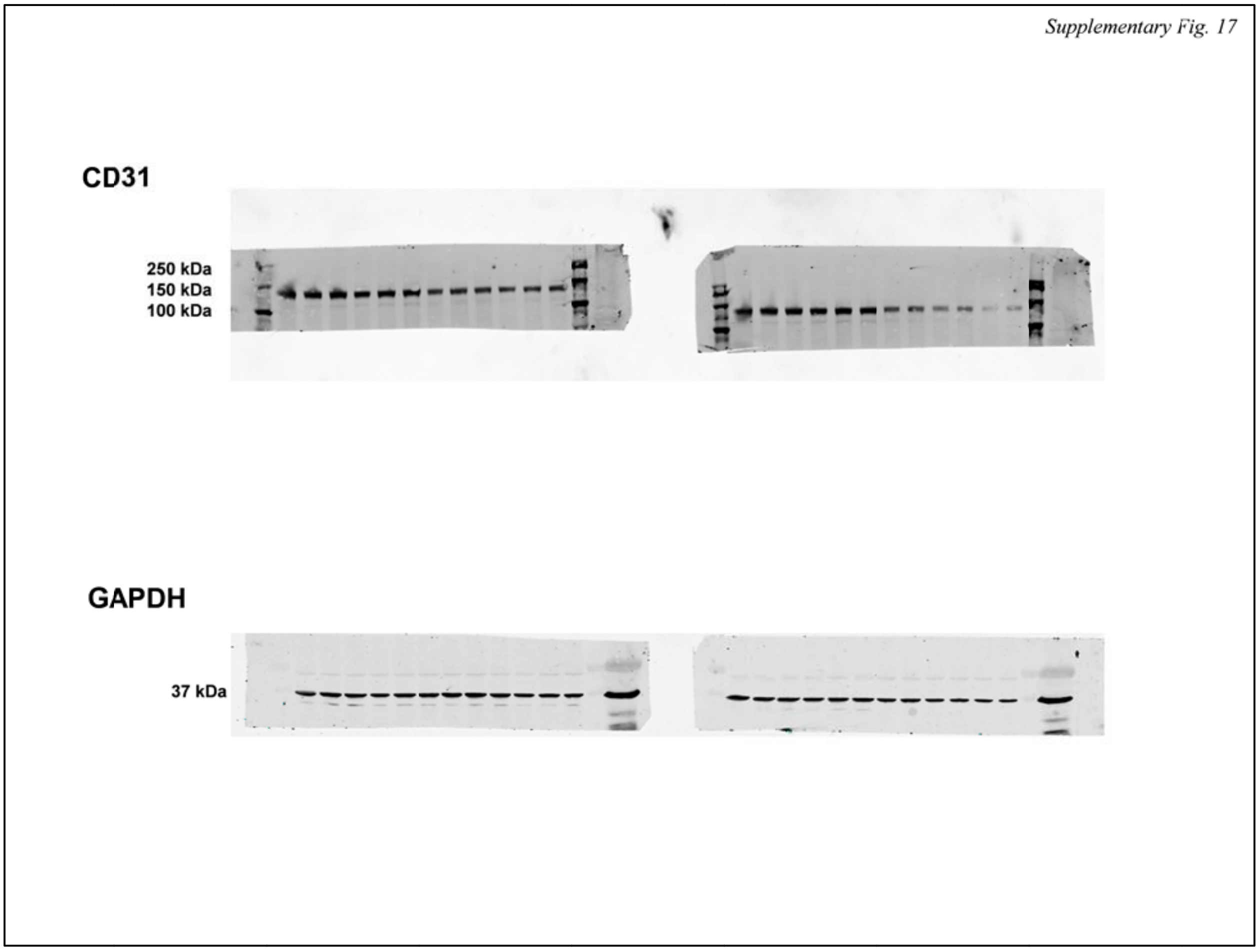

Supplementary Fig. 17. Original immunoblots from Fig. 3g. Ladder marker sizes shown on left. CD31 expected molecular weight/size is 130kDa. GAPDH expected molecular weight/size is 37kDa. 2 membranes were imaged adjacent to one another. On the right membranes, the first 6 lanes were additional control lanes and not included in the main Figures.

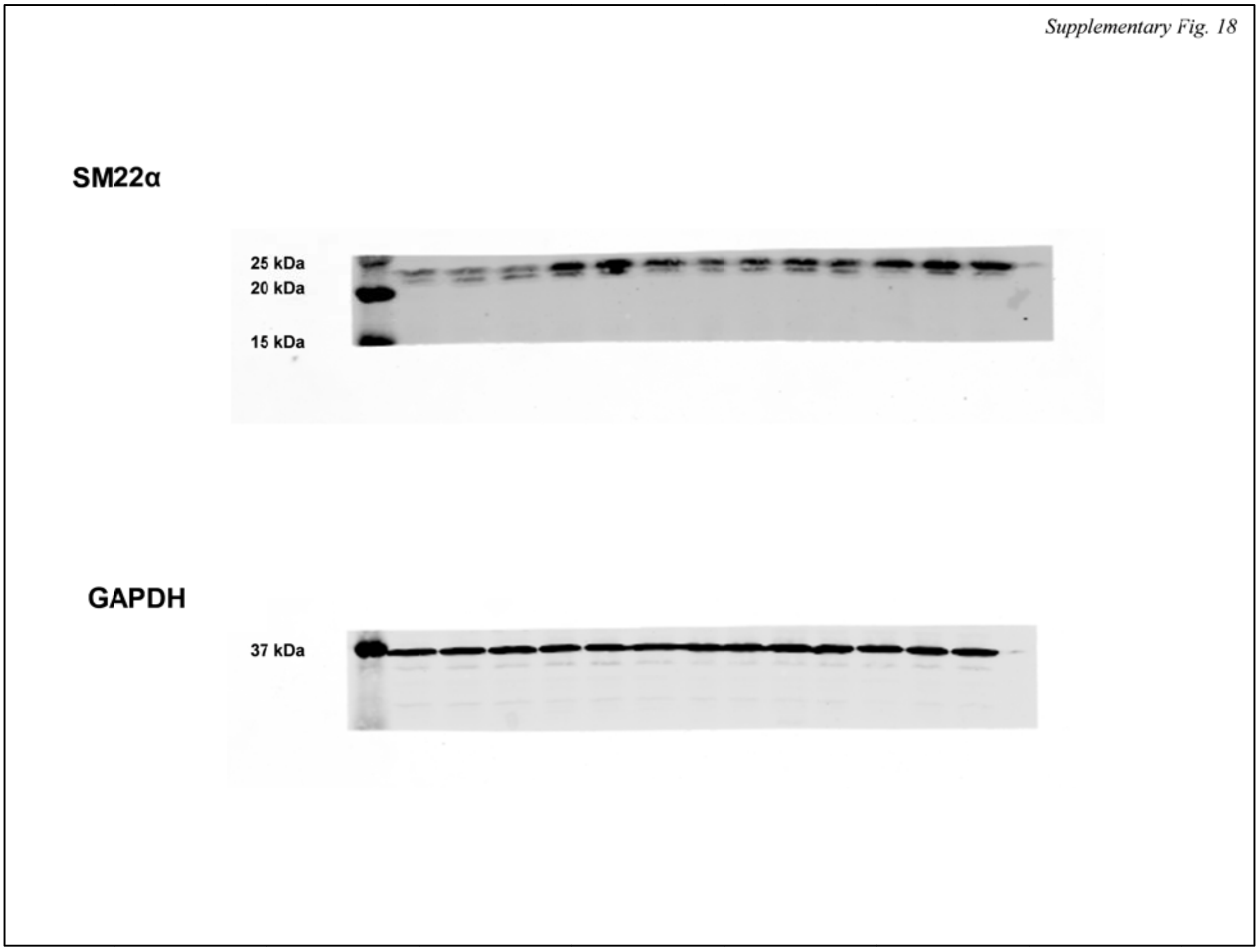

Supplementary Fig. 18. Original immunoblots from Fig. 4m. Ladder marker sizes shown on left. SM22α expected molecular weight/size is 23kDa. GAPDH expected molecular weight/size is 37kDa. Note that a final additional lane is present (most rightward lane), which represented an additional sample (4th) sample under conditions of combined hypoxia and TGF-β treatment.

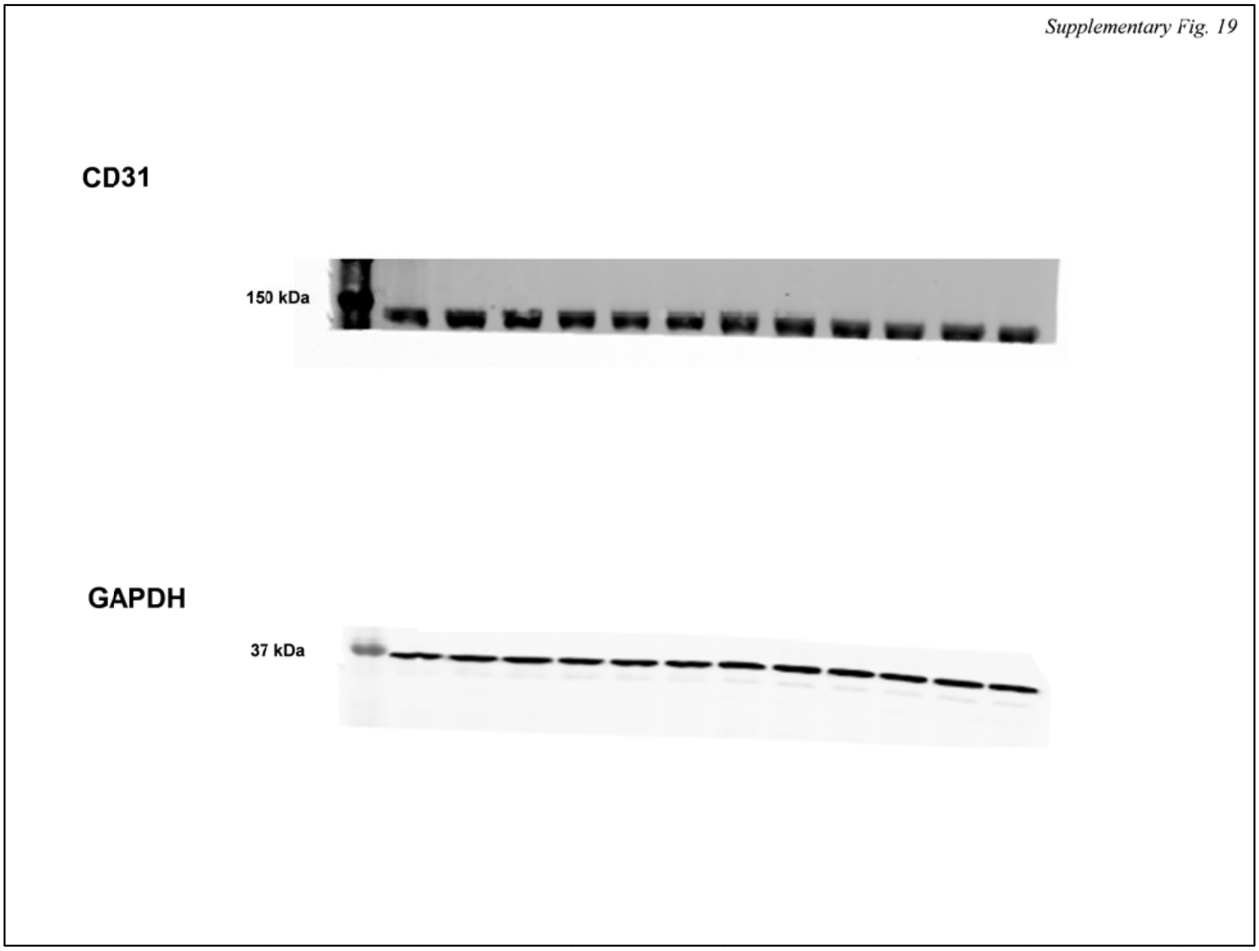

Supplementary Fig. 19. Original immunoblots from Fig. 4n. Ladder marker sizes shown on left. CD31 expected molecular weight/size is 130kDa. GAPDH expected molecular weight/size is 37kDa.

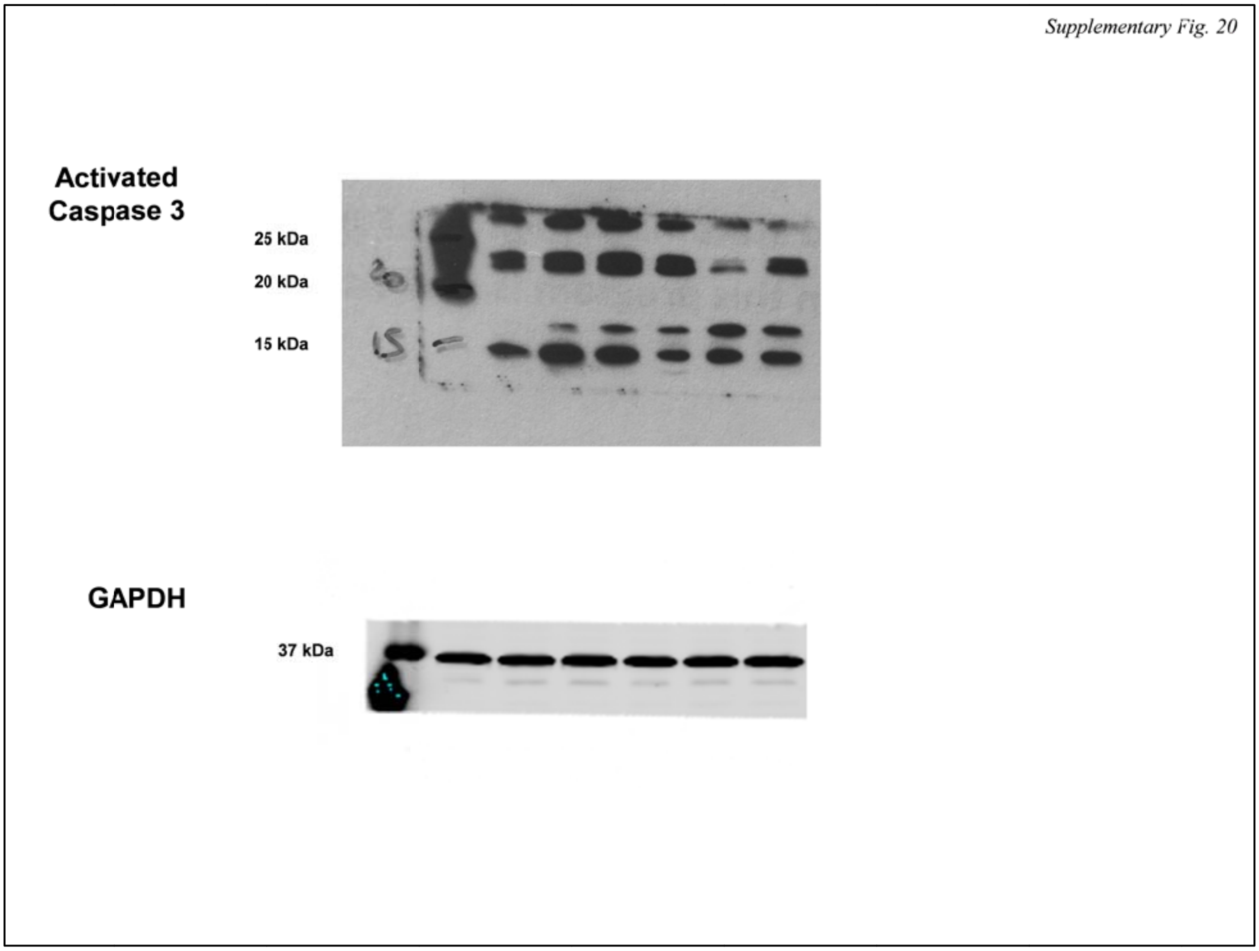

Supplementary Fig. 20. Original immunoblots from Supplementary Fig. 9c. Ladder marker sizes shown on left. Activated Caspase 3 expected molecular weight/size is 17kDa. GAPDH expected molecular weight/size is 37kDa.

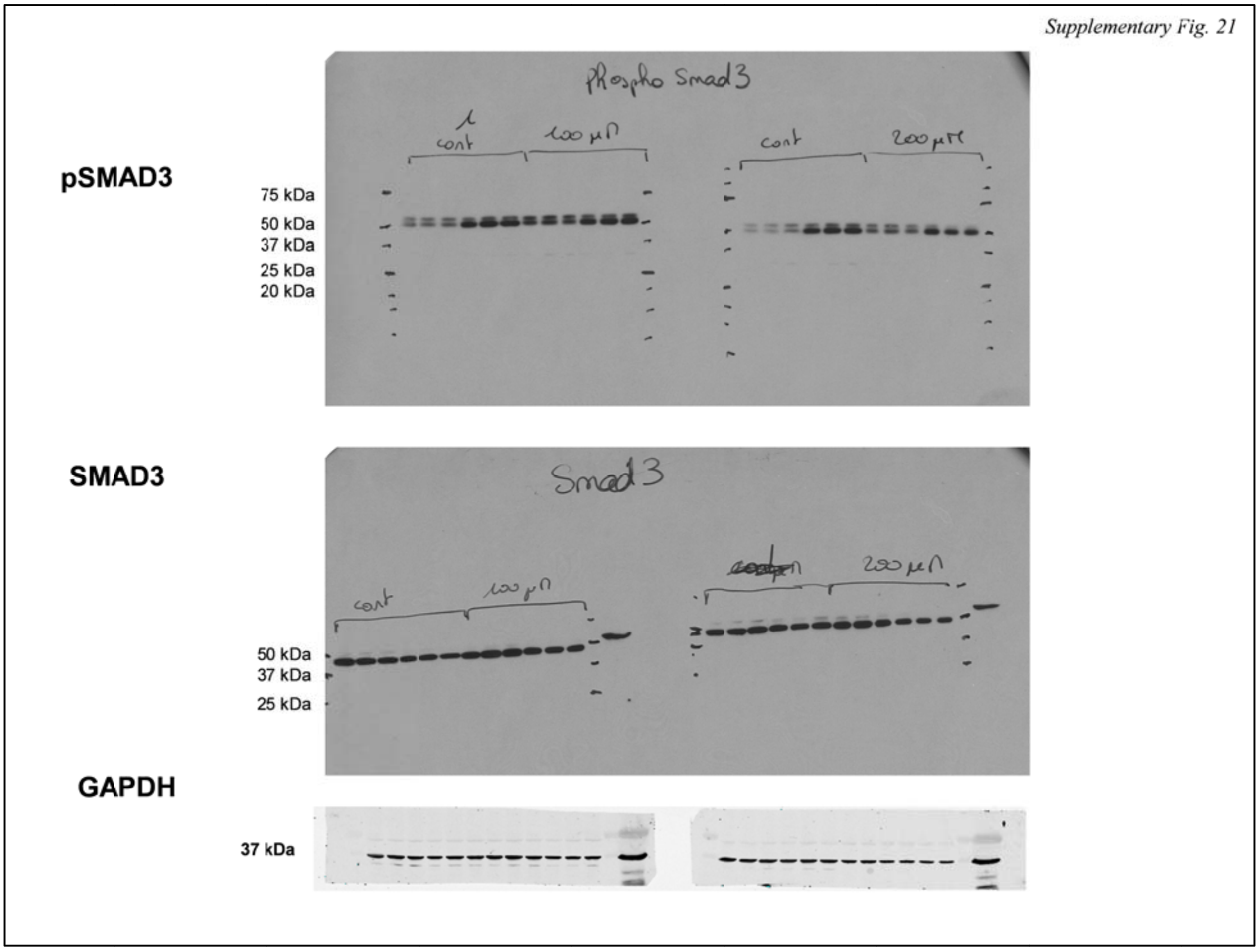

Supplementary Fig. 21. Original immunoblots from Supplementary Fig. 10g. Ladder marker sizes shown on left. pSMAD3 expected molecular weight/size is 48kDa. SMAD3 expected molecular weight/size is 52kDa. GAPDH expected molecular weight/size is 37kDa. On the right membranes, the first 6 lanes were additional control lanes and not included in the main Figures.

Supplementary Table 1. Complete results of 2-way ANOVA analyses for Supplementary Fig. 9, representing comparisons of HUVECs exposed to TGF-β and/or H_2O_2.

Figure	Event studied	Source of variation (% of total variation, p value)			Bonferroni posttest p value						
		Inter-action	H_2O_2	TGF-β	No TGF-β vs. TGF-β			No H_2O_2 vs. 100µM H_2O_2		No H_2O_2 vs. 200µM H_2O_2	
					No H_2O_2	100µM H_2O_2	200µM H_2O_2	No TGF-β	TGF-β	No TGF-β	TGF-β
Suppl 9a	Number of cells	0.07%; P = 0.96	89.49%; P < 0.0001	1.15%; P = 0.25	ns	ns	ns	< 0.01	< 0.05	< 0.001	< 0.001
Suppl 9b	BrdU incorporation	1.50%; P = 0.29	91.55%; P < 0.0001	0.37%; P = 0.43	ns	ns	ns	< 0.0001	< 0.001	< 0.0001	< 0.0001
Suppl 9e	% dead cells	0.26%; P = 0.94	57.35%; P = 0.0004	1.49%; P = 0.43	ns	ns	ns	ns	ns	< 0.01	< 0.01

Supplementary Table 2. List of genes and transcription factors presented in Fig. 3d that are altered in EndMT/EMT and supporting references.

**Abbreviated name**	**Full name**	**Alternative name**	**Reference**
**Endothelial**			
VWF	von Willebrand factor		1
CAV1	Caveolin-1		2
ICAM2	Intercellular adhesion molecule 2		3
PECAM1	Platelet/endothelial cell adhesion molecule 1	CD31	1
CDH5	Cadherin-5	VE-Cadherin; CD144	1
SOX18	SRY-related HMG-box 18		4
ENG	Endoglin	CD105	5
ESAM	Endothelial cell adhesion molecule		6
TEK		TIE2; CD202B	1
GPR116	G Protein-Coupled Receptor 1161		7
CD34	CD34		1
FLI1	Friend leukemia virus integration 1		8
EMCN	Endomucin		9
TIE1	Tyrosine kinase with immunoglobulin-like and EGF-like domains 1		10
LMO2	LIM domain only 2		11
EGFL7	EGF-like domain-containing protein 7	Vascular Endothelial-statin	12
SOX7	SRY (sex determining region Y)-box 7		13
NOS3	Nitric oxide synthase 3	eNOS	1
KDR	Kinase insert domain receptor	vascular endothelial growth factor receptor 2	14
LYVE1	Lymphatic vessel endothelial hyaluronan receptor 1	XLKD	15
ETV2	Ets Variant 2		13
SELP	P-selectin	CD62	16
CLDN3	Claudin-3	Tight junction protein	17
VCAM1	Vascular cell adhesion protein 1	CD106	18
SELE	E-selectin	CD62E	19
**Downregulated in EndMT/EMT**			
CAV2	Caveolin-2		20
KRT19	Keratin, type I cytoskeletal 1	cytokeratin-19	21

TP53	Tumor protein p53	p53	22
MTUS1	Mitochondrial tumor suppressor 1		23
CXADR	Coxsackievirus and adenovirus receptor	CAR	24
JUP	Junction Plakoglobin	Desmoplakin-3; gamma-catenin	25
DSP	Desmoplakin		26
**Upregulated in EndMT/EMT**			
ITGA5	Integrin, alpha 5	CD49e	27
CALD1	Caldesmon		28
MSN	Moesin		29
SPOCK1	Sparc/osteonectin, cwcv and kazal-like domains proteoglycan 1	Testican	30
SPARC	Secreted protein acidic and rich in cysteine	Osteonectin	31
ITGAV	Integrin alpha-V	CD51	32
AHNAK	AHNAK	Desmoyokin	33
NOTCH1			34
JAG1	Jagged 1	CD339	35
SLC22A4	Solute carrier family 22, member 4		36
ZEB2	Zinc finger E-box-binding homeobox 2	Sip1	37
CXCR4	C-X-C chemokine receptor type 4	CD184	38
ZEB1	Zinc finger E-box-binding homeobox 1		39
HEY2	Hairy/enhancer-of-split related with YRPW motif protein 2	CHF1	39, 40
SNAI1		SNAIL	41
SNAI2		SLUG	41
HEY1	Hairy/enhancer-of-split related with YRPW motif protein 1		42
TWIST1	Twist-related protein 1	TWIST	41
WNT5B	Wingless-type MMTV integration site family, member 5B		43
WNT11	Wingless-type MMTV integration site family, member 11		44
HEYL	Hairy/enhancer-of-split related with YRPW motif-like protein		45
VPS13A	Vacuolar protein sorting-associated protein 13A		46, 47
**Mesenchymal**			
VIM	Vimentin		48
SERPINE1	Serpin peptidase inhibitor, clade E (nexin, plasminogen activator inhibitor type 1), member 1	Plasminogen activator inhibitor-1	49

CTGF	Connective tissue growth factor	CCN2	50<br>36
SRGN	Serglycin	PPG; PRG1	36
TUBA1A	Tubulin alpha-1A chain		36
MYH9	Myosin, heavy chain 9	NMHC-IIA	51
TPM1	Tropomyosin alpha-1 chain		52
TAGLN	Transgelin	SM22; SM22α	41
CDH2	Cadherin-2	neural cadherin (NCAD)	53
COL5A1	Collagen, type V, alpha 1		54
NT5E	5'-nucleotidase	CD73	55
COL5A2	Collagen, type V, alpha 2		56
SRF	Serum response factor		57
ACTA2	Alpha-actin-2	alpha smooth muscle actin (αSMA)	41
PLAT	Tissue plasminogen activator	tPA	58
PLAU	Urokinase-type plasminogen activator		49
CDH11	Cadherin-11	CAD11	56
P4HA1	Prolyl 4-hydroxylase subunit alpha-1	P4HA	59
SERPINE 2	Serpin peptidase inhibitor, clade E (nexin, plasminogen activator inhibitor type 1), member 2		60
PTX3	Pentraxin-related protein PTX3		56
RECK	Reversion-inducing-cysteine-rich protein with kazal motifs		36
FBLN5	Fibulin-5		56<br>36
MMP2	Matrix metalloproteinase-2		61
PLAUR	Urokinase receptor	CD87	60
PRKCA	Protein kinase C alpha	PKCα	36
NEXN	Nexilin	F-actin binding protein	41
MMP14	Matrix metalloproteinase-14		60
POSTN	Periostin	Osteoblast specific factor	56
DLC1	Deleted in Liver Cancer 1	STARD12	56<br>36
COL1A2	Collagen, type I, alpha 2		56
FN1	Fibronectin		52
PLEK2	Pleckstrin-2		60
COL6A1	Collagen, type VI, alpha 1		62
IGFBP3	Insulin-like growth factor-binding protein 3		36
VCAN	Versican		63
COL3A1	Collagen, type III, alpha 1		56

NOTCH3	Neurogenic locus notch homolog protein 3	CADASIL	64
CD248		Endosialin; TEM1	65<br>66
FAP	Fibroblast activation protein	DPPIV	56
S100A4	S100 calcium-binding protein A4		67
CNN1	CCN family member 1	Cysteine-rich angiogenic inducer 61 (CYR61)	68
COL1A1	Collagen, type I, alpha 1		69<br>36
ADAM12	Disintegrin and metalloproteinase domain-containing protein 12		70<br>36
NID2	Nidogen-2		36
SPP1	Secreted phosphoprotein 1	Osteopontin; OPN	71
CD44			72
FBLN1	Fibulin-1		56<br>36
DDR2	Discoidin domain-containing receptor 2		56
MMP9	Matrix metalloproteinase-9		61

Supplementary Table 3. Complete results of 2-way ANOVA analyses for Fig. 3e - h, representing comparisons of HUVECs exposed to TGF-β and/or H_2O_2.

Figure	Event studied	Source of variation (% of total variation, p value)			Bonferroni posttest p value						
		Inter-action	H_2O_2	TGF-β	No TGF-β vs. TGF-β			No H_2O_2 vs. 100µM H_2O_2		No H_2O_2 vs. 200µM H_2O_2	
					No H_2O_2	100µM H_2O_2	200µM H_2O_2	No TGF-β	TGF-β	No TGF-β	TGF-β
Figure 3e	FAP protein levels	3.4%; P = 0.059	83.50%; P < 0.0001	7.46%; P = 0.0018	< 0.05	ns	< 0.05	< 0.05	ns	< 0.001	< 0.001
Figure 3f	*FAP* RNA levels	11.65%; P = 0.0012	27.16%; P < 0.0001	33.49%; P < 0.0001	ns	< 0.05	< 0.001	ns	ns	ns	< 0.001
Figure 3g	CD31 protein levels	8.34%; P = 0.017	73.53%; P < 0.0001	3.99%; P = 0.039	ns	ns	< 0.01	< 0.001	< 0.01	< 0.001	< 0.001
Figure 3h	*CD31* RNA levels	9.58%; P = 0.057	35.03%; P = 0.001	0.32%; P = 0.65	ns	ns	ns	< 0.001	ns	< 0.001	< 0.05

Supplementary Table 4. Complete results of 2-way ANOVA analyses for Supplementary Fig. 10, representing comparisons of HUVECs exposed to TGF-β and/or H_2O_2.

Figure	Event studied	Source of variation (% of total variation, p value)			Bonferroni posttest p value						
		Inter-action	H_2O_2	TGF-β	No TGF-β vs. TGF-β			No H_2O_2 vs. 100µM H_2O_2		No H_2O_2 vs. 200µM H_2O_2	
					No H_2O_2	100µM H_2O_2	200µM H_2O_2	No TGF-β	TGF-β	No TGF-β	TGF-β
Suppl 10a	Calponin RNA levels	13.85%; P = 0.0020	21.37%; P = 0.0001	33.55%; P < 0.0001	ns	ns	< 0.001	ns	ns	ns	< 0.001
Suppl 10b	Versican RNA levels	7.95%; P = 0.017	12.47%; P = 0.0023	52.88%; P < 0.0001	< 0.05	< 0.01	< 0.001	ns	ns	ns	< 0.001
Suppl 10c	*SNAI2* RNA levels	11.86%; P = 0.0016	36.84%; P < 0.0001	26.77%; P < 0.0001	ns	ns	< 0.001	ns	ns	ns	< 0.001
Suppl 10d	*SNAI1* RNA levels	1.64%; P = 0.34	4.25%; P = 0.071	68.17%; P < 0.0001	< 0.001	< 0.001	< 0.001	ns	< 0.05	ns	ns
Suppl 10e	*SMAD2* RNA levels	0.19%; P = 0.97	0.16%; P = 0.82	8.02%; P = 0.28	ns	ns	ns	ns	ns	ns	ns
Suppl 10f	*SMAD3* RNA levels	2.51%; P = 0.31	47.85%; P < 0.0001	19.19%; P = 0.0001	ns	ns	< 0.01	< 0.05	< 0.01	< 0.001	< 0.001
Suppl 10g	SMAD3 protein levels	13.45%; P = 0.091	26.35%; P = 0.015	19.27%; P = 0.012	ns	ns	< 0.05	ns	ns	< 0.01	ns
Suppl 10g	pSMAD3 protein levels	5.33%; P = 0.0076	0.88%; P = 0.36	65.74%; P < 0.0001	< 0.001	< 0.001	< 0.001	< 0.01	ns	ns	ns

Supplementary Table 5. Complete results of 2-way ANOVA analyses for Supplementary Fig. 12, representing comparisons of HCAECs exposed to TGF-β and/or H_2O_2.

Figure	Event studied	Source of variation (% of total variation, p value)			Bonferroni posttest p value			
		Inter-action	H_2O_2	TGF-β	No TGF-β vs. TGF-β		No H_2O_2 vs. 200µM H_2O_2	
					No H_2O_2	200µM H_2O_2	No TGF-β	TGF-β
Suppl 12b	*FAP* RNA levels	5.79%; P = 0.080	10.73%; P = 0.021	55.52%; P < 0.0001	< 0.05	< 0.001	ns	< 0.05
Suppl 12c	*DDR2* RNA levels	0.9%; P = 0.54	34.44%; P = 0.0009	19.45%; P = 0.0082	ns	< 0.05	< 0.01	ns
Suppl 12d	*SM22α* RNA levels	17.03%; P = 0.0005	33.44%; P < 0.0001	29.87%; P < 0.0001	ns	< 0.001	ns	< 0.001
Suppl 12e	Calponin RNA levels	7.51%; P = 0.031	13.38%; P = 0.0057	51.12%; P < 0.0001	< 0.05	< 0.001	ns	< 0.01
Suppl 12f	Versican RNA levels	17.49%; P = 0.0004	23.35%; P < 0.0001	40.08%; P < 0.0001	ns	< 0.001	ns	< 0.001
Suppl 12g	*CD31* RNA levels	5.75%; P = 0.25	10.21%; P = 0.13	1.13%; P = 0.61	ns	ns	ns	ns
Suppl 12h	*TIE2* RNA levels	0.02%; P = 0.95	11.43%; P = 0.12	2.64%; P = 0.44	ns	ns	ns	ns
Suppl 12i	*SNAI2* RNA levels	10.97%; P = 0.0064	37.20%; P < 0.0001	28.17%; P < 0.0001	ns	< 0.001	ns	< 0.001
Suppl 12j	*SNAI1* RNA levels	20.10%; P = 0.0099	4.70%; P = 0.18	25.63%; P = 0.0043	< 0.001	ns	< 0.05	ns

Supplementary Table 6. PCR primers.

Gene	Primers		Length of PCR products (base pairs)
	Forward	Reverse	
Mouse Genotyping Primers			
ApoE	GCCTAGCCGAGGGAGAGCCG	(WT) TGTGACTTGGGAGCTCTGCAGC<br>(Mutant) GCCGCCCCGACTGCATCT	Mutant: 245<br>WT: 155
Cre	ATTGCTGTCACTTGGTCGTGG	GAAAATGCTTCTGTCCGTTTGC	200
YFP	AAAGTCGCTCTGAGTTGTTAT	(WT) GGAGCGGGAGAAATGGATATG<br>(Mutant) GCGAAGAGTTTGTCCTCAACC	Mutant: 320<br>WT: 600
Human Primers			
18S rRNA	TTTCGGAACTGAGGCCATGA	GCAAATGCTTTCGCTCTGGTC	110
FAP	CAAGTGGCAAGTGGGAGGCCA	TGGGGATGCCTGGGCCGTAG	246
DDR2	GACTTGCACACCCTCCATTT	GAGTGGTCGGTGACTGGAAT	251
SM22α	CCTGGCTAGGGAAACCCACCCT	TCTGGGGAAAGCTCCTTGGAAGT	300
Calponin	GGCCAGCATGGCGAAGACGAAA	TGTGCCCAGCTTGGGGTCGT	205
CD31	GCGAGTCATGGCCCGAAGGC	GGTGGTGCTGACATCCGCGA	246
SNAI1	GCACGGCCTAGCGAGTGGTT	GGGCTGCTGGAAGGTAAACTCTGG	171
SNAI2	AACTCACACGGGGGAGAAGCCT	CAGTGTGCTACACAGCAGCCAGA	192
SMAD2	AGTGCCCCGACACACCGAGA	TCTGCTGGAGAGCCTGTGTCC	207
SMAD3	TCAAGAAGACGGGGCAGCTGGA	GCACCAACACAGGAGGTAGAACTGG	297
TIE2	GCAATGAAGCATGCCACCCTGG	GGTAGCGGCCAGCCAGAAGC	241
Versican	GGTGCACTTTGTGAGCAAGA	TTCGTGAGACAGGATGCTTG	159
Ve-Cadherin	AACTTCCCCTTCTTCACCC	AAAGGCTGCTGGAAAATG	368
eNOS	GTGGCTGTCTGCATGGACCT	CCACGATGGTGACTTTGGCT	121
KDR	TGCCTACCTCACCTGTTTC	GGCTCTTTCGCTTACTGTTC	114

Supplementary References

1. Pereira, C.F., *et al.* Induction of a hemogenic program in mouse fibroblasts. *Cell Stem Cell* **13**, 205-218 (2013).
2. Bauer, P.M., *et al.* Endothelial-specific expression of caveolin-1 impairs microvascular permeability and angiogenesis. *Proc Natl Acad Sci U S A* **102**, 204-209 (2005).
3. Cowan, P.J., *et al.* The human ICAM-2 promoter is endothelial cell-specific in vitro and in vivo and contains critical Sp1 and GATA binding sites. *J Biol Chem* **273**, 11737-11744 (1998).
4. Hosking, B.M., *et al.* SOX18 directly interacts with MEF2C in endothelial cells. *Biochem Biophys Res Commun* **287**, 493-500 (2001).
5. Cheifetz, S., *et al.* Endoglin is a component of the transforming growth factor-beta receptor system in human endothelial cells. *J Biol Chem* **267**, 19027-19030 (1992).
6. Elcheva, I., *et al.* Direct induction of haematoendothelial programs in human pluripotent stem cells by transcriptional regulators. *Nature communications* **5**, 4372 (2014).
7. Mancuso, P., *et al.* A subpopulation of circulating endothelial cells express CD109 and is enriched in the blood of cancer patients. *PLoS One* **9**, e114713 (2014).
8. Asano, Y., *et al.* Endothelial Fli1 deficiency impairs vascular homeostasis: a role in scleroderma vasculopathy. *Am J Pathol* **176**, 1983-1998 (2010).
9. Liu, C., *et al.* Human endomucin is an endothelial marker. *Biochem Biophys Res Commun* **288**, 129-136 (2001).
10. Partanen, J., *et al.* A novel endothelial cell surface receptor tyrosine kinase with extracellular epidermal growth factor homology domains. *Mol Cell Biol* **12**, 1698-1707 (1992).
11. Gratzinger, D., *et al.* The transcription factor LMO2 is a robust marker of vascular endothelium and vascular neoplasms and selected other entities. *American journal of clinical pathology* **131**, 264-278 (2009).
12. Parker, L.H., *et al.* The endothelial-cell-derived secreted factor Egfl7 regulates vascular tube formation. *Nature* **428**, 754-758 (2004).
13. Behrens, A.N., *et al.* Sox7 is regulated by ETV2 during cardiovascular development. *Stem Cells Dev* **23**, 2004-2013 (2014).
14. Terman, B.I., *et al.* Identification of a new endothelial cell growth factor receptor tyrosine kinase. *Oncogene* **6**, 1677-1683 (1991).
15. Gordon, E.J., Gale, N.W. & Harvey, N.L. Expression of the hyaluronan receptor LYVE-1 is not restricted to the lymphatic vasculature; LYVE-1 is also expressed on embryonic blood vessels. *Dev Dyn* **237**, 1901-1909 (2008).
16. Polley, M.J., *et al.* CD62 and endothelial cell-leukocyte adhesion molecule 1 (ELAM-1) recognize the same carbohydrate ligand, sialyl-Lewis x. *Proc Natl Acad Sci U S A* **88**, 6224-6228 (1991).
17. Vincent, T., *et al.* A SNAIL1-SMAD3/4 transcriptional repressor complex promotes TGF-beta mediated epithelial-mesenchymal transition. *Nat Cell Biol* **11**, 943-950 (2009).
18. Cybulsky, M.I. & Gimbrone, M.A., Jr. Endothelial expression of a mononuclear leukocyte adhesion molecule during atherogenesis. *Science* **251**, 788-791 (1991).
19. Collins, T., *et al.* Structure and chromosomal location of the gene for endothelial-leukocyte adhesion molecule 1. *J Biol Chem* **266**, 2466-2473 (1991).
20. Dragoi, A.M., Swiss, R., Gao, B. & Agaisse, H. Novel strategies to enforce an epithelial phenotype in mesenchymal cells. *Cancer Res* **74**, 3659-3672 (2014).
21. Loriot, C., *et al.* Epithelial to mesenchymal transition is activated in metastatic pheochromocytomas and paragangliomas caused by SDHB gene mutations. *The Journal of clinical endocrinology and metabolism* **97**, E954-962 (2012).

22. Chang, C.J., *et al.* p53 regulates epithelial-mesenchymal transition and stem cell properties through modulating miRNAs. *Nat Cell Biol* **13**, 317-323 (2011).
23. Zhao, T., Ding, X., Chang, B., Zhou, X. & Wang, A. MTUS1/ATIP3a down-regulation is associated with enhanced migration, invasion and poor prognosis in salivary adenoid cystic carcinoma. *BMC Cancer* **15**, 203 (2015).
24. Lacher, M.D., *et al.* Transforming growth factor-beta receptor inhibition enhances adenoviral infectability of carcinoma cells via up-regulation of Coxsackie and Adenovirus Receptor in conjunction with reversal of epithelial-mesenchymal transition. *Cancer Res* **66**, 1648-1657 (2006).
25. Holen, I., *et al.* Loss of plakoglobin promotes decreased cell-cell contact, increased invasion, and breast cancer cell dissemination in vivo. *Breast cancer research : BCR* **14**, R86 (2012).
26. Tsuji, T., *et al.* Epithelial-mesenchymal transition induced by growth suppressor p12CDK2-AP1 promotes tumor cell local invasion but suppresses distant colony growth. *Cancer Res* **68**, 10377-10386 (2008).
27. Qin, L., *et al.* Steroid receptor coactivator-1 upregulates integrin alpha(5) expression to promote breast cancer cell adhesion and migration. *Cancer Res* **71**, 1742-1751 (2011).
28. Morita, T., Mayanagi, T. & Sobue, K. Dual roles of myocardin-related transcription factors in epithelial mesenchymal transition via slug induction and actin remodeling. *J Cell Biol* **179**, 1027-1042 (2007).
29. Haynes, J., Srivastava, J., Madson, N., Wittmann, T. & Barber, D.L. Dynamic actin remodeling during epithelial-mesenchymal transition depends on increased moesin expression. *Mol Biol Cell* **22**, 4750-4764 (2011).
30. Miao, L., *et al.* SPOCK1 is a novel transforming growth factor-beta target gene that regulates lung cancer cell epithelial-mesenchymal transition. *Biochem Biophys Res Commun* **440**, 792-797 (2013).
31. Fenouille, N., *et al.* The epithelial-mesenchymal transition (EMT) regulatory factor SLUG (SNAI2) is a downstream target of SPARC and AKT in promoting melanoma cell invasion. *PLoS One* **7**, e40378 (2012).
32. Wehbe, M., *et al.* Epithelial-mesenchymal-transition-like and TGFbeta pathways associated with autochthonous inflammatory melanoma development in mice. *PLoS One* **7**, e49419 (2012).
33. Shankar, J., *et al.* Pseudopodial actin dynamics control epithelial-mesenchymal transition in metastatic cancer cells. *Cancer Res* **70**, 3780-3790 (2010).
34. Xie, M., *et al.* Activation of Notch-1 enhances epithelial-mesenchymal transition in gefitinib-acquired resistant lung cancer cells. *Journal of cellular biochemistry* **113**, 1501-1513 (2012).
35. Leong, K.G., *et al.* Jagged1-mediated Notch activation induces epithelial-to-mesenchymal transition through Slug-induced repression of E-cadherin. *The Journal of experimental medicine* **204**, 2935-2948 (2007).
36. Groger, C.J., Grubinger, M., Waldhor, T., Vierlinger, K. & Mikulits, W. Meta-analysis of gene expression signatures defining the epithelial to mesenchymal transition during cancer progression. *PLoS One* **7**, e51136 (2012).
37. Vandewalle, C., *et al.* SIP1/ZEB2 induces EMT by repressing genes of different epithelial cell-cell junctions. *Nucleic acids research* **33**, 6566-6578 (2005).
38. Li, X., *et al.* The SDF-1/CXCR4 axis induces epithelial-mesenchymal transition in hepatocellular carcinoma. *Molecular and cellular biochemistry* **392**, 77-84 (2014).
39. Wellner, U., *et al.* The EMT-activator ZEB1 promotes tumorigenicity by repressing stemness-inhibiting microRNAs. *Nature cell biology* **11**, 1487-1495 (2009).
40. Fischer, A., *et al.* Combined loss of Hey1 and HeyL causes congenital heart defects because of impaired epithelial to mesenchymal transition. *Circ Res* **100**, 856-863 (2007).

41. Cooley, B.C., *et al.* TGF-beta Signaling Mediates Endothelial-to-Mesenchymal Transition (EndMT) During Vein Graft Remodeling. *Science translational medicine* **6**, 227ra234 (2014).
42. Zavadil, J., Cermak, L., Soto-Nieves, N. & Bottinger, E.P. Integration of TGF-beta/Smad and Jagged1/Notch signalling in epithelial-to-mesenchymal transition. *EMBO J* **23**, 1155-1165 (2004).
43. Kato, S., Hayakawa, Y., Sakurai, H., Saiki, I. & Yokoyama, S. Mesenchymal-transitioned cancer cells instigate the invasion of epithelial cancer cells through secretion of WNT3 and WNT5B. *Cancer science* **105**, 281-289 (2014).
44. Zhang, P., Cai, Y., Soofi, A. & Dressler, G.R. Activation of Wnt11 by transforming growth factor-beta drives mesenchymal gene expression through non-canonical Wnt protein signaling in renal epithelial cells. *J Biol Chem* **287**, 21290-21302 (2012).
45. Bielesz, B., *et al.* Epithelial Notch signaling regulates interstitial fibrosis development in the kidneys of mice and humans. *J Clin Invest* **120**, 4040-4054 (2010).
46. Alsarraj, J., *et al.* Deletion of the proline-rich region of the murine metastasis susceptibility gene Brd4 promotes epithelial-to-mesenchymal transition- and stem cell-like conversion. *Cancer Res* **71**, 3121-3131 (2011).
47. Loeffler, I., Liebisch, M. & Wolf, G. Collagen VIII influences epithelial phenotypic changes in experimental diabetic nephropathy. *American journal of physiology. Renal physiology* **303**, F733-745 (2012).
48. Mendez, M.G., Kojima, S. & Goldman, R.D. Vimentin induces changes in cell shape, motility, and adhesion during the epithelial to mesenchymal transition. *Faseb J* **24**, 1838-1851 (2010).
49. Risolino, M., *et al.* Transcription factor PREP1 induces EMT and metastasis by controlling the TGF-beta-SMAD3 pathway in non-small cell lung adenocarcinoma. *Proc Natl Acad Sci U S A* **111**, E3775-3784 (2014).
50. Shafieian, M., Chen, S. & Wu, S. Integrin-linked kinase mediates CTGF-induced epithelial to mesenchymal transition in alveolar type II epithelial cells. *Pediatric research* **77**, 520-527 (2015).
51. Beach, J.R., *et al.* Myosin II isoform switching mediates invasiveness after TGF-beta-induced epithelial-mesenchymal transition. *Proc Natl Acad Sci U S A* **108**, 17991-17996 (2011).
52. Gervasi, M., *et al.* JunB contributes to Id2 repression and the epithelial-mesenchymal transition in response to transforming growth factor-beta. *J Cell Biol* **196**, 589-603 (2012).
53. Serova, M., *et al.* Epithelial-to-mesenchymal transition and oncogenic Ras expression in resistance to the protein kinase Cbeta inhibitor enzastaurin in colon cancer cells. *Mol Cancer Ther* **9**, 1308-1317 (2010).
54. Joseph, J.V., *et al.* TGF-beta is an inducer of ZEB1-dependent mesenchymal transdifferentiation in glioblastoma that is associated with tumor invasion. *Cell death & disease* **5**, e1443 (2014).
55. Xiong, L., Wen, Y., Miao, X. & Yang, Z. NT5E and FcGBP as key regulators of TGF-1-induced epithelial-mesenchymal transition (EMT) are associated with tumor progression and survival of patients with gallbladder cancer. *Cell Tissue Res* **355**, 365-374 (2014).
56. Taube, J.H., *et al.* Core epithelial-to-mesenchymal transition interactome gene-expression signature is associated with claudin-low and metaplastic breast cancer subtypes. *Proc Natl Acad Sci U S A* **107**, 15449-15454 (2010).
57. Park, M.Y., *et al.* Expression of the serum response factor in hepatocellular carcinoma: implications for epithelial-mesenchymal transition. *International journal of oncology* **31**, 1309-1315 (2007).
58. Yang, J., *et al.* Disruption of tissue-type plasminogen activator gene in mice reduces renal interstitial fibrosis in obstructive nephropathy. *J Clin Invest* **110**, 1525-1538 (2002).
59. Gilkes, D.M., Bajpai, S., Chaturvedi, P., Wirtz, D. & Semenza, G.L. Hypoxia-inducible factor 1 (HIF-1) promotes extracellular matrix remodeling under hypoxic conditions by inducing P4HA1, P4HA2, and PLOD2 expression in fibroblasts. *J Biol Chem* **288**, 10819-10829 (2013).

60. Sarrio, D., *et al.* Epithelial-mesenchymal transition in breast cancer relates to the basal-like phenotype. *Cancer Res* **68**, 989-997 (2008).
61. Wiercinska, E., *et al.* The TGF-beta/Smad pathway induces breast cancer cell invasion through the up-regulation of matrix metalloproteinase 2 and 9 in a spheroid invasion model system. *Breast cancer research and treatment* **128**, 657-666 (2011).
62. Jechlinger, M., *et al.* Expression profiling of epithelial plasticity in tumor progression. *Oncogene* **22**, 7155-7169 (2003).
63. Inai, K., Burnside, J.L., Hoffman, S., Toole, B.P. & Sugi, Y. BMP-2 induces versican and hyaluronan that contribute to post-EMT AV cushion cell migration. *PLoS One* **8**, e77593 (2013).
64. Liu, L., *et al.* Notch3 is important for TGF-beta-induced epithelial-mesenchymal transition in non-small cell lung cancer bone metastasis by regulating ZEB-1. *Cancer gene therapy* **21**, 364-372 (2014).
65. Nanda, A., *et al.* Tumor endothelial marker 1 (Tem1) functions in the growth and progression of abdominal tumors. *Proc Natl Acad Sci U S A* **103**, 3351-3356 (2006).
66. Lax, S., *et al.* CD248 expression on mesenchymal stromal cells is required for post-natal and infection-dependent thymus remodelling and regeneration. *FEBS open bio* **2**, 187-190 (2012).
67. Mirza, A., *et al.* Investigation of the epithelial to mesenchymal transition markers S100A4, vimentin and Snail1 in gastroesophageal junction tumors. *Diseases of the esophagus : official journal of the International Society for Diseases of the Esophagus / I.S.D.E* **27**, 485-492 (2014).
68. Haque, I., *et al.* Cyr61/CCN1 signaling is critical for epithelial-mesenchymal transition and stemness and promotes pancreatic carcinogenesis. *Molecular cancer* **10**, 8 (2011).
69. Tan, X., Dagher, H., Hutton, C.A. & Bourke, J.E. Effects of PPAR gamma ligands on TGF-beta1-induced epithelial-mesenchymal transition in alveolar epithelial cells. *Respir Res* **11**, 21 (2010).
70. Li, H., Duhachek-Muggy, S., Dubnicka, S. & Zolkiewska, A. Metalloproteinase-disintegrin ADAM12 is associated with a breast tumor-initiating cell phenotype. *Breast cancer research and treatment* **139**, 691-703 (2013).
71. Gujral, T.S., *et al.* A noncanonical Frizzled2 pathway regulates epithelial-mesenchymal transition and metastasis. *Cell* **159**, 844-856 (2014).
72. Cho, S.H., *et al.* CD44 enhances the epithelial-mesenchymal transition in association with colon cancer invasion. *International journal of oncology* **41**, 211-218 (2012).
